# Autocrine VEGF-B signaling maintains lipid synthesis and mitochondrial fitness to support T cell immune responses

**DOI:** 10.1172/JCI176586

**Published:** 2024-08-15

**Authors:** Jianli He, Yalan Chen, Huihua Ding, Jin-An Zhou, Zhengcao Xing, Xinyu Yang, Qiuju Fan, Yong Zuo, Tianshi Wang, Jinke Cheng

**Affiliations:** 1 Shanghai Key Laboratory for Tumor Microenvironment and Inflammation, Department of Biochemistry and Molecular Cell Biology,; 2 Department of Rheumatology, Renji Hospital,; 3 Shanghai Institute of Rheumatology, Renji Hospital, and; 4 State Key Laboratory of Systems Medicine for Cancer, Center for Single-Cell Omics, School of Public Health, Shanghai Jiao Tong University School of Medicine, Shanghai, China.; 5 Hainan Medical University, Hainan Academy of Medical Sciences, Haikou, Hainan, China.

**Keywords:** Immunology, Adaptive immunity, Growth factors, T cells

## Abstract

T cells rewire their metabolic activities to meet the demand of immune responses, but how to coordinate the immune response by metabolic regulators in activated T cells is unknown. Here, we identified autocrine VEGF-B as a metabolic regulator to control lipid synthesis and maintain the integrity of the mitochondrial inner membrane for the survival of activated T cells. Disruption of autocrine VEGF-B signaling in T cells reduced cardiolipin mass, resulting in mitochondrial damage, with increased apoptosis and reduced memory development. The addition of cardiolipin or modulating VEGF-B signaling improved T cell mitochondrial fitness and survival. Autocrine VEGF-B signaling through GA-binding protein α (GABPα) induced sentrin/SUMO-specific protease 2 (SENP2) expression, which further de-SUMOylated PPARγ and enhanced phospholipid synthesis, leading to a cardiolipin increase in activated T cells. These data suggest that autocrine VEGF-B mediates a signal to coordinate lipid synthesis and mitochondrial fitness with T cell activation for survival and immune response. Moreover, autocrine VEGF-B signaling in T cells provides a therapeutic target against infection and tumors as well as an avenue for the treatment of autoimmune diseases.

## Abstract 2

Autocrine VEGF-B mediates a signal to coordinate lipid synthesis and mitochondrial fitness with T cell activation for survival and immune response.

## Graphical abstract

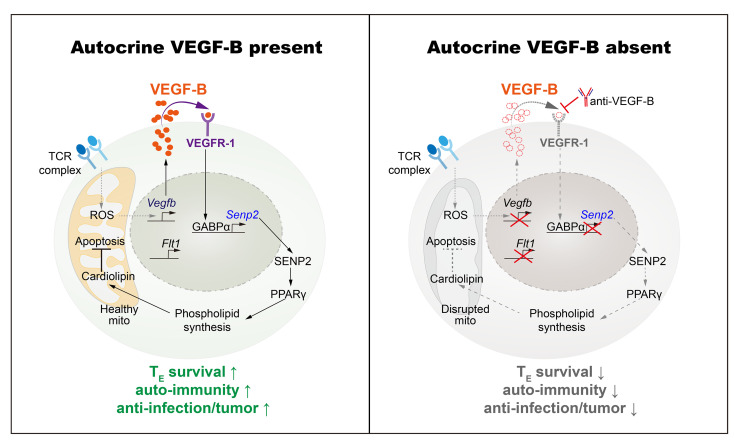

## Introduction

T cells rewire metabolic activity to meet their own activation demand in response to antigens (1–4). Glucose metabolism in T cells, which increases glucose anabolic metabolism and biomass accumulation to support their proliferation (5, 6), is well studied. Lipid metabolism is emerging as a crucial metabolic pathway in T cell responses (7–11). Lipids supply energy sources for T cells, especially for memory T cells or regulatory T cells, via a process of fatty acid oxidation (10–12). Lipids are also used as a nutrient source for biomass generation in the T cell immune response (12–14). In addition, lipid-derived derivates act as regulators of intracellular signaling in T cell activation and differentiation (7, 8, 15), such as in the synthesis of cardiolipin (CL), which is required for the memory development of CD8^+^ T cells (13). Nevertheless, modulation of lipid metabolism in coordination with T cell activation is not well studied.

The function of the main organelle to regulate cellular metabolism, the mitochondria, supports T cell activation and survival (12, 16). It is worth noting that mitochondrial ROS production is required for antigen-specific T cell responses (16). These ROS-mediated responses are alleviated owing to an apoptosis increase (17). Insufficient apoptosis of activated T cells results in autoimmune disorders, while excessive apoptosis causes T cell immunodeficiency (18–21). After the peak of a T cell response, only a small fraction of activated T cells survived and entered the memory pool (22). Therefore, antiapoptosis factors are essential for T cell mitochondrial function and memory development (23). However, it is still not fully understood whether the mechanism underlying the dynamics of apoptosis occurs in the course of the mitochondrial ROS-mediated immune response.

Autocrine in mammals is a crucial for regulation of cell fate (24–28). As activated T cells are effector cells with powerful secretion, they kill or regulate target cells by secreting cytokines and also regulate T cell proliferation and differentiation in an autocrine manner (24–28). One such cytokine, autocrine IL-2, is required for the proliferation, differentiation, and memory development of CD8^+^ T cells (24–26). In Th cells, autocrine IL-21 induced by an IL-6/STAT3-dependent mechanism is essential for the differentiation of inflammatory Th17 cells (27). Moreover, IL-17A limits the pathogenicity of Th17 cells by inducing autocrine IL-24 (28). Recently, it was demonstrated that autocrine vitamin D signaling initiated the transition from proinflammatory IFN-γ^+^ Th1 cells to suppressive IL-10^+^ cells (29). In addition to immune-associated cytokines, autocrine metabolic regulators are identified as a cluster of secretory factors that regulate the state of cell metabolism in an autocrine manner, including myokines, hepatokines, adipokines, and other growth factors (30–32). For example, FGF21 promotes thermogenic gene expression in white adipose tissue through autocrine signaling (33, 34). It has been postulated that an autocrine signaling may modulate the metabolic program to be coordinated with the T cell–mediated immune response (1, 4, 18). However, it is unclear whether T cells modulate their cell metabolism and immune response through autocrine metabolic regulators. In this study, we identified autocrine VEGF-B as an upstream regulator that activates the GABPα/SENP2/PPARγ axis for lipid synthesis during T cell activation and found that autocrine VEGF-B signaling coordinated mitochondrial fitness via CL synthesis is required for T cell survival and immune response.

## Results

### TCR activation induces VEGF-B expression and secretion in T cells.

**Figure 1:**
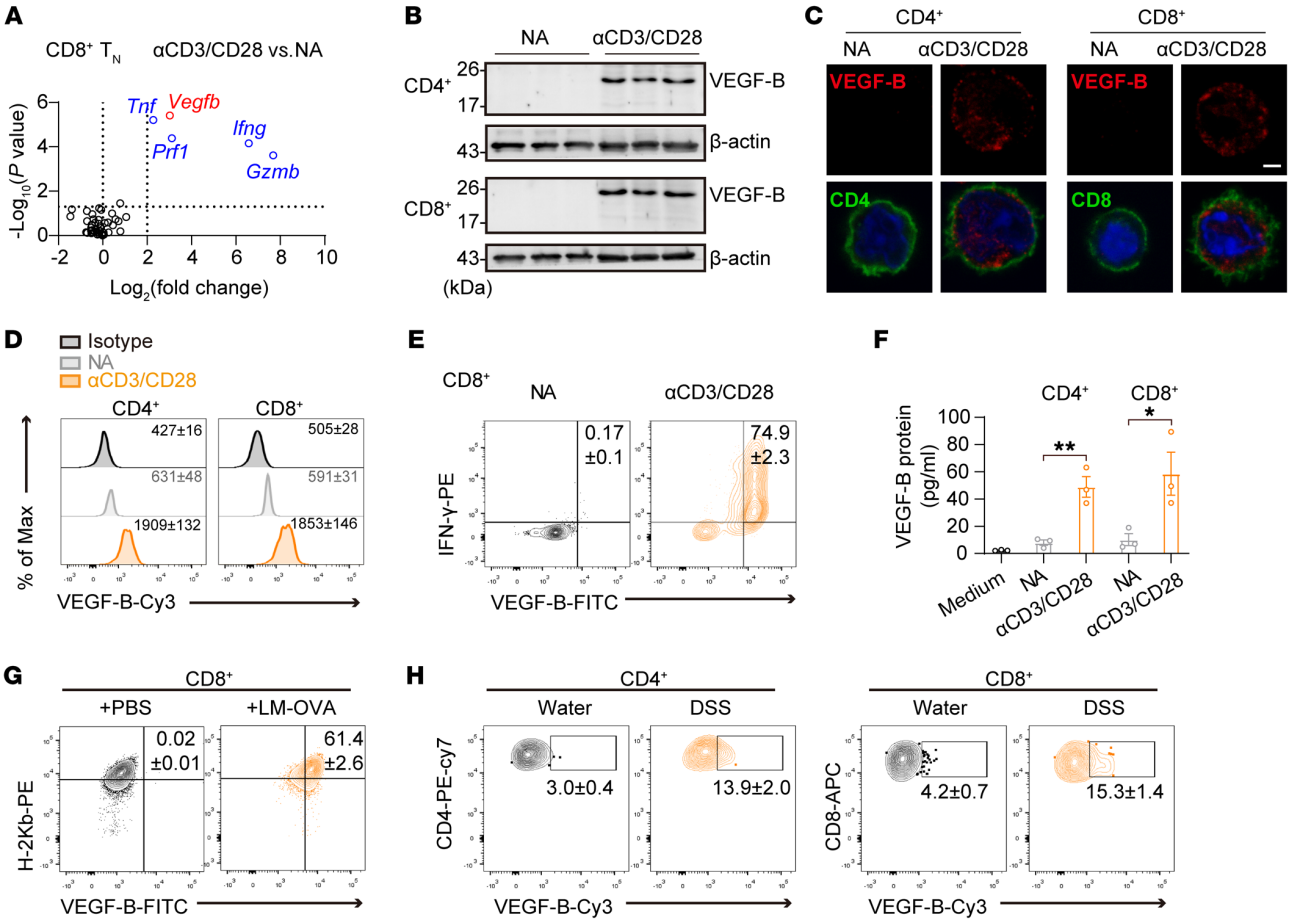
TCR activation induces VEGF-B protein expression in T cells. (**A**) Relative mRNA levels of the secreted factors in CD8^+^ Tn cells treated with or without (NA) αCD3/CD28 for 3 days; *n* = 3. *Prf1*, *Ifng*, *Gzmb*, and *Tnf* were positive indicators for T cell activation. (**B**) VEGF-B protein levels in NA or activated CD4^+^ and CD8^+^ T cells; *n* = 3. (**C**) Immunofluorescence images of VEGF-B in NA or activated CD4^+^ or CD8^+^ T cells. DAPI staining is in blue. Scale bar: 2 μm. (**D**) The mean fluorescent intensity (MFI) of VEGF-B in NA or activated CD4^+^ or CD8^+^ T cells; *n* = 3. (**E**) The population of VEGF-B^+^ and IFN-γ^+^ in NA or activated CD8^+^ T cells; *n* = 3. (**F**) VEGF-B protein levels in culture supernatants of NA or activated CD4^+^ or CD8^+^ T cells; *n* = 3. (**G**) The population of VEGF-B^+^ and H-2Kb^+^ cells in CD8^+^ T cells from the spleen of OT-1 mice infected by LM-OVA for 3 days; *n* = 5. (**H**) The population of VEGF-B^+^ cells in CD4^+^ or CD8^+^ T cells from colon tissue with DSS-induced colitis; *n* = 6. Data are shown as mean ± SEM. *P* values were calculated using 2-tailed unpaired *t* test in **F**. *P < 0.05, **P < 0.01.

To assess the autocrine metabolic regulators for T cell metabolism regulation, we examined potential candidates, including myokines (*OSM, Mstn,* *Fndc5*, etc.), hepatokines (*Angptl6, Ahsg,* *Shbg*, etc.), adipokines (*Nampt, Adipoq, Lep*, etc.), and other secreted factors (30–32). RNA quantification and microarray data showed that *Vegfb* was upregulated in T cell receptor–stimulated (TCR-stimulated) CD8^+^ naive T (Tn) cells (Figure 1A and Supplemental Figure 1, A and B; supplemental material available online with this article; https://doi.org/10.1172/JCI176586DS1) (35). VEGF-B protein expression was further validated in both TCR-activated CD4^+^ and CD8^+^ T cells (Figure 1, B–E). Importantly, VEGF-B protein was detected in the medium from TCR-activated T cells (Figure 1F), suggesting that VEGF-B was a secreted factor by activated T cells. In addition, the time-course analysis showed that VEGF-B upregulation appeared at 48–96 hours after TCR stimulation (Supplemental Figure 1C), indicating that VEGF-B induction mainly occurred in the expansion state of activated T cells. Moreover, VEGF-B expression in different subsets of mouse T cells was determined, in which the effector memory T cells exhibited a higher VEGF-B^+^ cell population than the central memory T cells or Tn cells in the spleen (Supplemental Figure 1D). We then examined the protein levels of VEGF-B in antigen-specific activated T cells in vivo. Ovalbumin-expressing *Listeria monocytogenes*–infected (LM-OVA–infected) OT-1 mice showed a much higher VEGF-B^+^ cell population in antigen-specific tetramer H-2Kb^+^ CD8^+^ effector T cells than the OT-1 mice without infection (Figure 1G). Similarly, the intestines of DSS-induced acute colitis mice had a higher population of VEGF-B^+^ cells in both CD4^+^ and CD8^+^ T cells as compared with the intestines of control mice (Figure 1H and Supplemental Figure 1E). These data indicate that VEGF-B is specifically expressed in the activation status of both CD4^+^ and CD8^+^ T cells.

### Mitochondrial ROS triggers VEGF-B expression in TCR-activated T cells.

**Figure 2:**
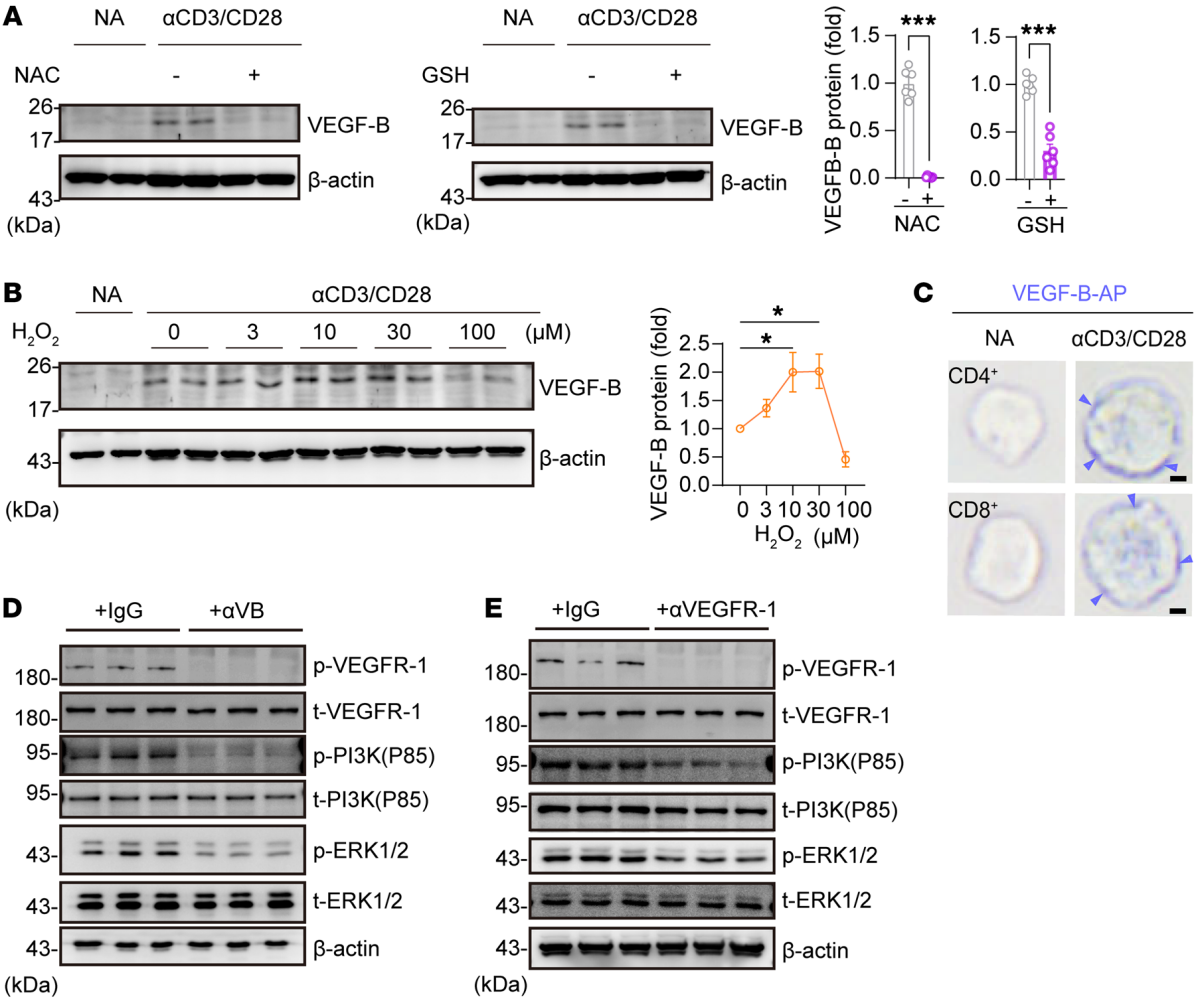
ROS-triggered VEGF-B protein acts on the VEGFR-1 expressed on activated T cells. (**A**) VEGF-B protein levels in activated CD8^+^ T cells treated with N-acetyl-L-cysteine (NAC) (5 μM) or glutathione (GSH) (5 μM); *n* = 6. (**B**) VEGF-B protein levels in activated CD8^+^ T cells treated with H_2_O_2_ at a gradient concentration; *n* = 3. (**C**) Alkaline phosphatase–labeled VEGF-B protein (VEGF-B-AP) binding in NA or activated CD4^+^ or CD8^+^ T cells. Scale bar: 2 μm. (**D** and **E**) The phosphorylation levels of VEGFR-1 (phospho-tyrosine), PI3K (P85), or ERK1/2 in activated CD8^+^ T cells treated by anti–VEGF-B (αVB) or anti–VEGFR-1 (αVEGFR-1) antibodies; *n* = 3. Data are shown as mean ± SEM. *P* values were calculated using 2-tailed unpaired *t* test in **A** and 1-way ANOVA with Holm-Šídák’s post hoc test in **B**. *P < 0.05, ***P < 0.001.

VEGF-B has been shown to express in endothelial cells and is associated with mitochondrial biogenesis (36). We showed that the VEGF-B level was positively correlated with the mitochondrial mass in TCR-activated T cells (Supplemental Figure 2A). Treatment with PGC-1α activator ZLN005, mitochondrial uncoupler CCCP, mitochondrial complex I inhibitor rotenone, or mitochondrial complex III inhibitor antimycin altered VEGF-B expression in TCR-activated T cells (Supplemental Figure 2, B–E), suggesting that VEGF-B expression was related to mitochondrial respiration during T cell activation. Since increased mitochondrial ROS is indispensable in T cell activation (16, 37), we thus reasoned that mitochondrial ROS might be a trigger for VEGF-B expression in activated T cells. Indeed, treatment with antioxidants, such as N-acetyl-L-cysteine, glutathione, or mitochondria-targeted antioxidant mitoTEMPO, blocked TCR-induced VEGF-B expression in CD8^+^ T cells (Figure 2A and Supplemental Figure 2F). As fatty acid oxidation is associated with mitochondrial ROS production, we found that the inhibition of fatty acid oxidation by etomoxir or fatty acid synthesis by C75 also significantly suppressed the expression of VEGF-B in activated T cells (Supplemental Figure 2, G and H). Furthermore, the addition of H_2_O_2_ at a lower dosage increased VEGF-B expression in TCR-activated T cells. However, a higher dosage of H_2_O_2_ (at 100 mm) decreased VEGF-B expression (Figure 2B). It has been reported that a high level of ROS is a factor in causing detrimental effects or severe cell damage in the contraction stage of activated T cells (38–40). The results suggest that the expression of VEGF-B induced by TCR stimulation is triggered by mitochondrial ROS signaling.

### VEGF-B acts on the VEGFR-1 expressed on T cells.

To explore the potential target cells of VEGF-B secreted by activated T cells, we checked the DICE (Database of Immune Cell Expression, Expression quantitative trait loci, and Epigenomics) database (41), which shows that the VEGF-B receptor VEGFR-1 (encoded by the *FLT1* gene) is specifically expressed in both activated CD4^+^ and CD8^+^ T cells (Supplemental Figure 3A). Similar to the expression dynamics of VEGF-B, VEGFR-1 was also significantly elevated at 48–96 hours after TCR stimulation (Supplemental Figure 3B). We then confirmed that VEGF-B could bind to the surface of activated T cells by staining activated T cells with alkaline phosphatase–labeled VEGF-B protein (Figure 2C). Moreover, we confirmed that anti–VEGF-B–neutralizing antibody or anti–VEGFR-1 antibody blocked VEGFR-1 phosphorylation as well as decreased VEGFR downstream signaling PI3K (P85) or ERK1/2 phosphorylation in TCR-activated T cells (Figure 2, D and E). The results suggest that TCR activation–induced VEGF-B may act on T cells themselves through an autocrine loop.

### Autocrine VEGF-B is essential for T cell survival and memory development.

**Figure 3:**
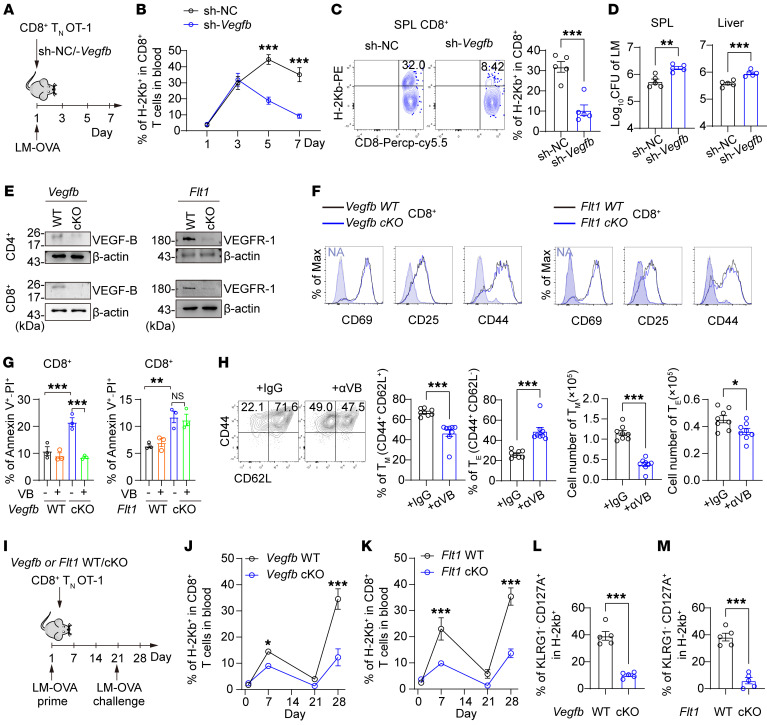
Autocrine VEGF-B is required for the survival of activated T cells. (**A**–**D**) sh-*Vegfb* or negative control (sh-NC) CD8^+^ OT-1 Tn cells were transferred into recipients infected with LM-OVA. The H-2Kb^+^ ratio in CD8^+^ T cells from the blood (**B**) or spleen (**C**) of recipients; *n* = 5. (**D**) The LM CFUs in the spleen and liver of the infected recipients; *n* = 5. (**E**) VEGF-B and VEGFR-1 protein levels in WT and cKO T cells; *n* = 3. (**F**) The MFI of CD69, CD25, and CD44 in *Vegfb* or *Flt1* WT and cKO CD8^+^ Tn cells with TCR activation; *n* = 3. (**G**) The annexin V^+^ and PI^+^ ratio in VEGF-B–treated (VB-treated) *Vegfb* or *Flt1* WT and cKO CD8^+^ T cells; *n* = 3. (**H**) CD8^+^ Tn cells were activated to induce memory T cells (Tm) by IL-15. The numbers and ratios of Tm (CD44^+^CD62L^+^) or T_E_ (CD44^+^CD62L^–^) cells with IgG or αVB treatment were calculated; *n* = 8. (**I**–**M**) *Vegfb* or *Flt1* WT and cKO CD8^+^ OT-1 T cells were transferred to recipients, followed by LM-OVA infection. (**J** and **K**) H-2Kb^+^ ratio in CD8^+^ T cells from the blood of recipients; *n* = 5. (**L** and **M**) The memory precursor (KLRG1^–^CD127A^+^) ratio in H-2Kb^+^ CD8^+^ T cells from the spleen of recipients on day 7 of prime infection; *n* = 5. Data are shown as mean ± SEM. *P* values were calculated using 2-tailed unpaired *t* test in **C**, **D**, **H**, **L**, and **M** and 2-way ANOVA with Holm-Šídák’s post-hoc test in **B**, **G**, **J**, and **K**. *P < 0.05, **P < 0.01, ***P < 0.001.

We further explored whether autocrine VEGF-B signaling modulated T cell responses. To verify the role of autocrine VEGF-B in antigen-specific CD8^+^ T cell responses, we adoptively transferred *Vegfb*-silenced OT-1 CD8^+^ T cells to recipient mice, followed by infection with LM-OVA (Figure 3A and Supplemental Figure 4A). Silencing *Vegfb* resulted in an attenuated antigen-specific response of CD8^+^ T cells and a decrease of antigen-specific (H2-Kb^+^) CD8^+^ T cells in the spleens of recipient mice (Figure 3, B and C, and Supplemental Figure 4, B and C). Importantly, the bacterial burden in the spleen or liver was higher in sh-*Vegfb* OT-1 CD8^+^ T cell–transplanted mice than in the control mice (Figure 3D). We further generated T cell–specific *Vegfb*-knockout (*Cd4*^cre^ × *Vegfb*^fl/fl^, *Vegfb-*cKO) or *Flt1*-knockout (*Cd4*^cre^ × *Flt1*^fl/fl^, *Flt1-*cKO) mice (Figure 3E and Supplemental Figure 5A). Consistently, *Vegfb*-cKO mice infected with LM showed increased CFUs of LM in the spleen or liver as compared with WT mice, and the populations of effector CD8^+^ T cells (IFN-γ^+^, GZMB^+^, or TNF-α^+^) and effector CD4^+^ T cells (IFN-γ^+^ or IL-17A^+^) were lower in the spleens of *Vegfb*-cKO mice infected with LM than in those of WT mice with the infection (Supplemental Figure 5, B–D). Meanwhile, by analyzing the mean fluorescence of cytokines in effector cells, we found that there was no change in the production of cytokines per effector T cell (Supplemental Figure 5, B and C). These results suggest that autocrine VEGF-B regulates the anti-infection ability of CD8^+^ cells by affecting the proportion of effector T cells.

To explore the process of autocrine VEGF-B regulating the strength of the T cell response, we analyzed the effects of *Vegfb* or *Flt1* deficiency on T cell development and activation ex vivo. Our analysis showed that *Vegfb* or *Flt1* deficiency did not affect T cell development in the thymus or the early activation of T cells (Figure 3F and Supplemental Figure 5, E–G). However, we observed that neutralizing VEGF-B significantly reduced the cell viability and increased the apoptosis of both CD4^+^ and CD8^+^ T cells on day 3 after TCR activation (Supplemental Figure 5, H and I). Consistently, the *Vegfb*-deficient or *Flt1*-deficient T cells exhibited increased apoptosis when compared with WT T cells with TCR activation ex vivo or antigen-specific activation in vivo (Supplemental Figure 4C and Supplemental Figure 5, J and K). At the molecular level, we found that the classical apoptotic pathway of mitochondrial cytochrome *c* release and caspase-3 cleavage was activated in TCR-stimulated CD4^+^ or CD8^+^ T cells with *Vegfb* knockout (Supplemental Figure 5L). Importantly, the addition of VEGF-B (1 ng/mL) rescued the viability of *Vegfb*-deficient T cells but not that of *Flt1*-deficient T cells upon activation (Figure 3G), suggesting that autocrine VEGF-B played an essential role in preventing apoptosis of activated CD4^+^ and CD8^+^ T cells.

As the antiapoptotic ability of T cells also determined the development of memory, we further examined the effects of autocrine VEGF-B signaling on memory T cell development. Our results showed that, with IL-15 culture ex vivo, neutralizing VEGF-B signaling reduced the proportion and cell numbers of CD8^+^ memory T cells (CD44^+^CD62L^+^) while decreasing effector cell numbers (Figure 3H). Then, we adoptively transferred *Vegfb*- or *Flt1*-cKO and WT CD8^+^ OT-1 T cells to recipient mice, followed by prime and challenge infection with LM-OVA (Figure 3I). Consistent with the results of *Vegfb* knockdown by lentivirus, *Vegfb* or *Flt1* knockout resulted in a weakened antigen-specific response on day 7 of prime infection, and the survival of antigen-specific T cells decreased, starting at the contraction phase after 21 days (Figure 3, J and K, and Supplemental Figure 5, M and N). Furthermore, *Vegfb* or *Flt1* knockout resulted in a reduced antigen-specific recall of memory T cells after 7 days of challenge infection (Figure 3, J and K). We also demonstrated that *Vegfb* or *Flt1* deletion led to a decrease in the proportion of memory precursor effector cells in antigen-specific T cells on day 7 after prime infection (Figure 3, L and M, and Supplemental Figure 5O). By inducing the differentiation of CD4^+^ Th cells ex vivo, we also found that neutralization of VEGF-B signaling reduced the viability of Th cells, including IFN-γ^+^ Th1 cells, IL-4^+^ Th2 cells, IL-17A^+^ Th17 cells, and Foxp3^+^ Tregs but had no effect on the degree of differentiation (Supplemental Figure 6, A–D). To conclude, autocrine VEGF-B signaling induced by T cell activation regulates the strength of antigen-specific responses of T cells, and *Vegfb* or *Flt1* deficiency promotes apoptosis and reduces long-term survival and memory development in activated T cells.

### Autocrine VEGF-B promotes phospholipid synthesis to improve mitochondrial fitness in activated T cells.

**Figure 4:**
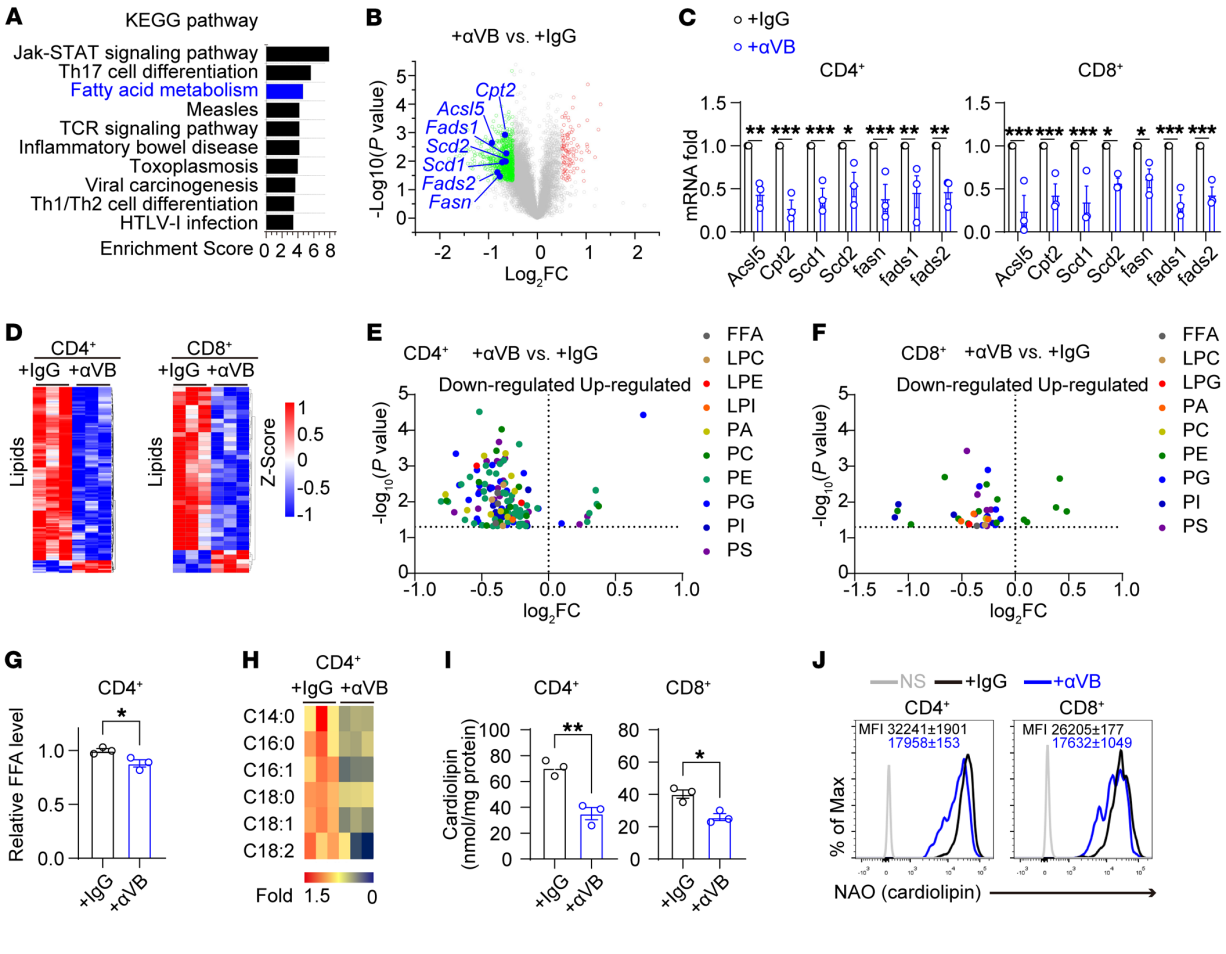
Autocrine VEGF-B is required for lipid synthesis in activated T cells. (**A** and **B**) RNA-Seq of TCR-stimulated CD4^+^ T cells treated with αVB or IgG; *n* = 3. (**A**) The enriched KEGG pathways and (**B**) the fatty acid metabolic genes in volcanic map. (**C**) The mRNA levels of fatty acid metabolic genes in T cells treated with αVB or IgG; *n* = 3. (**D**–**F**) The lipidomic analysis of CD4^+^ or CD8^+^ T cells; *n* = 3. (**D**) Significantly altered lipids in heat map and (**E** and **F**) volcanic diagrams. FC, fold change. (**G**) Relative free fatty acid (FFA) level in CD4^+^ T cells; *n* = 3. (**H**) The relative levels of long-chain acylcarnitine in CD4^+^ T cells; *n* = 3. (**I** and **J**) Cardiolipin levels in CD4^+^ or CD8^+^ T cells measured by (**I**) fluorometric assay kits (*n* = 3) or (**J**) by NAO staining (*n* = 3). Data are shown as mean ± SEM. *P* values were calculated using 2-tailed unpaired *t* test in **C**, **G**, and **I**. *P < 0.05, **P < 0.01, ***P < 0.001.

To determine how autocrine VEGF-B regulates T cell survival, an RNA-Seq analysis was performed to compare the transcriptome between activated T cells treated with and without anti–VEGF-B antibodies. Naive CD4^+^ T cells were isolated from the spleen upon stimulation with αCD3/CD28 antibodies prior to exposure to either an anti–VEGF-B antibody or IgG for 3 days. As a result, neutralizing VEGF-B altered the expression of 668 genes in activated T cells, including 599 downregulated genes and 69 upregulated genes (Supplemental Figure 7, A and B). As expected, the genes associated with the TCR signaling pathway and immune response were enriched in TCR-activated T cells under VEGF-B neutralization (Figure 4A). Interestingly, the downregulated genes associated with fatty acid metabolism were listed as the top 3 of the enriched pathways in activated T cells treated with anti–VEGF-B (Figure 4A), suggesting that VEGF-B might regulate fatty acid metabolism in T cell activation. It was evaluated that the downregulated genes related to fatty acid metabolism include lipid synthesis (*Fasn*, *Scd1*, *Scd2*, *Fads1*, and *Fads2*) and fatty acid transport (*Cpt2* and *Acls5*) in TCR-activated CD4^+^ or CD8^+^ T cells treated with anti–VEGF-B antibodies (Figure 4, B and C, and Supplemental Figure 7C). VEGF-B has been reported to promote endothelial uptake and transport of fatty acids in the heart and skeletal muscle (36, 42, 43). However, autocrine VEGF-B signaling mainly regulated the expression of lipid synthesis genes in TCR-activated T cells (Supplemental Figure 7C). It was true that neutralizing VEGF-B reduced total free fatty acids, long-chain acylcarnitine, and phospholipids, including PA, PE, PC, PS, PI, and PG, in TCR-activated CD4^+^ or CD8^+^ T cells (Figure 4, D–H). Additionally, fluorometric assay and CL fluorescent probe-10-N-Nonyl acridine orange (NAO) staining demonstrated that CL level was reduced by neutralizing VEGF-B in both TCR-activated CD4^+^ and CD8^+^ T cells (Figure 4, I and J). Consistent with our previous findings that inhibition of fatty acid oxidation or synthesis reduced VEGF-B expression in activated T cells, we also observed that inhibition of fatty acid oxidation or synthesis also led to reduced cardiolipin levels in activated T cells by NAO staining (Supplemental Figure 7D). These data suggest that a major role of autocrine VEGF-B signaling is to control phospholipid synthesis in activated T cells.

**Figure 5:**
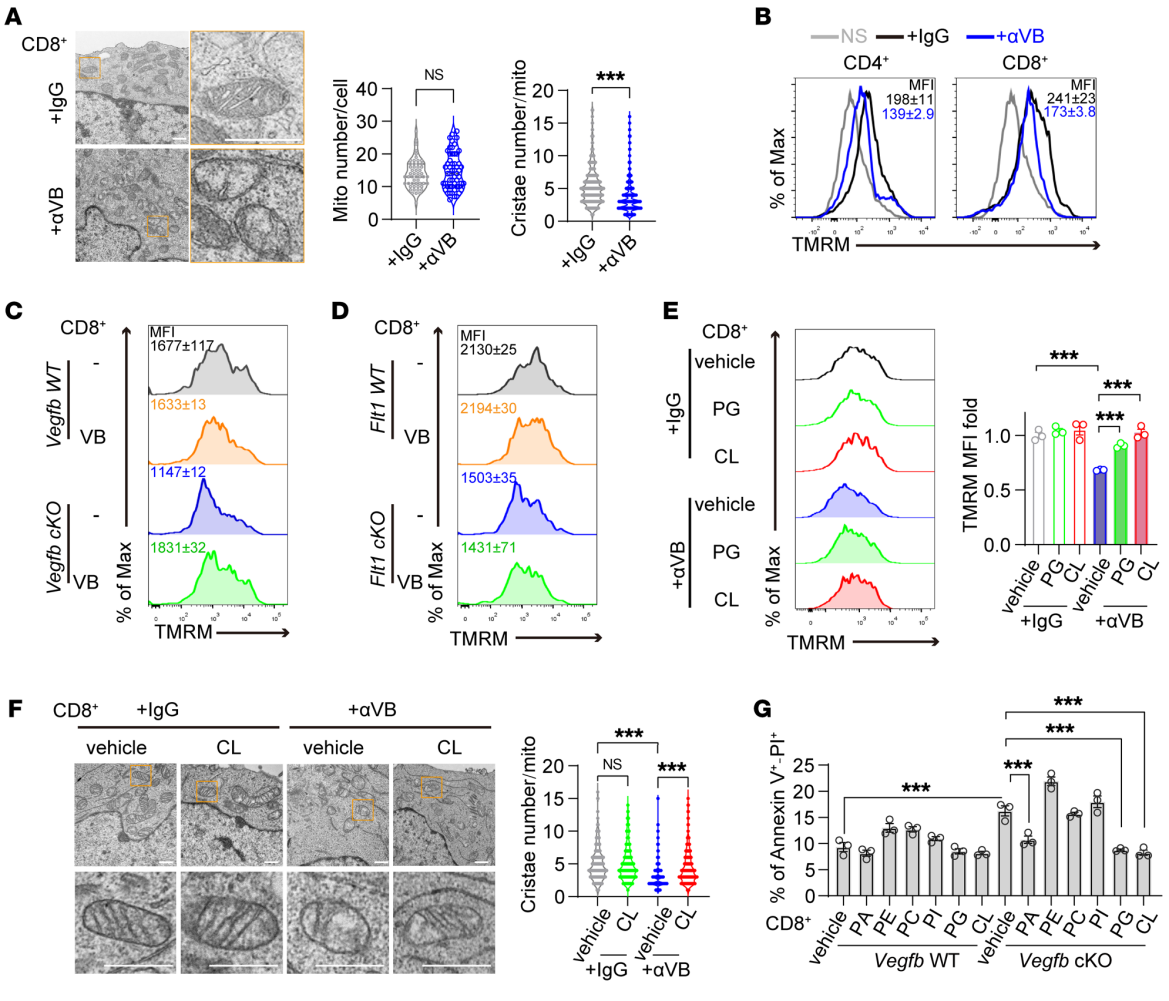
Autocrine VEGF-B maintains mitochondrial fitness in activated T cells. (**A**) Mitochondrial ultrastructure analyzed by transmission electron microscopy. The numbers of mitochondria (mito) per cell and cristae per mitochondrion were quantified in CD8^+^ cells treated with αVB or IgG. Scale bar: 500 nm. (**B**) The mitochondrial membrane potential (MMP) in TCR-stimulated T cells by Tetramethylrhodamine (TMRM, Invitrogen) staining; *n* = 3. (**C** and **D**) The MMP in TCR-stimulated *Vegfb* or *Flt1* WT and cKO CD8^+^ T cells with VB addition; *n* = 3. (**E**) The MMP in CD8^+^ T cells treated with αVB or IgG plus with or without PG or CL addition; *n* = 3. (**F**) The number of cristae per mitochondrion in CD8^+^ cells treated with αVB or IgG plus with or without CL addition. Scale bar: 500 nm. (**G**) The annexin V^+^ and PI^+^ ratios in CD8^+^ T cells with or without the addition of PA, PE, PC, PI, PG, or CL; *n* = 3. Data are shown as mean ± SEM. *P* values were calculated using 2-tailed unpaired *t* test in **A** and 2-way ANOVA with Bonferroni’s post hoc test in **E**–**G**. ***P < 0.001.

Given that CL is a dominant phospholipid in the mitochondrial inner membrane and binds to ETC complexes to regulate OXPHOS, cristae structure, and apoptosis (44, 45), we reasoned that CL synthesis might be essential for VEGF-B–mediated antiapoptosis in activated T cells. Indeed, neutralizing VEGF-B severely disrupted the architecture of mitochondrial inner membranes and reduced the mitochondrial membrane potential (MMP) in TCR-activated CD8^+^ T cells (Figure 5, A and B). Based on changes in mitochondrial ultrastructure, we performed a comprehensive analysis of mitochondrial function in T cells with *Vegfb* deletion. Through MitoTracker green and red staining, we confirmed that deletion of *Vegfb* led to a reduction in the MMP of CD4^+^ or CD8^+^ T cells without affecting their total mitochondrial content (Supplemental Figure 8A). Furthermore, we found that as the MMP decreased, deletion of *Vegfb* also resulted in reduced mitochondrial calcium influx, reduced levels of oxidative phosphorylation, and reduced mitochondrial ROS production in T cells, while their glycolytic activity was not affected (Supplemental Figure 8, B–E). Consistent with the results of ex vivo experiments, we observed suppressed expression of fatty acid synthesis genes, decreased cardiolipin levels, and reduced MMP in *Vegfb*-cKO CD8^+^ T cells in the spleens of mice with LM infection (Supplemental Figure 8, F–K). Interestingly, the addition of VEGF-B rescued the MMP in activated *Vegfb*-cKO but not in *Flt1-*cKO CD8^+^ or CD4^+^ T cells ex vivo (Figure 5, C and D, and Supplemental Figure 8L). More than that, the addition of CL recovered the MMP and mitochondrial cristae numbers in VEGF-B–neutralized CD8^+^ or CD4^+^ T cells (Figure 5, E and F, and Supplemental Figure 8, M and N). As CL is a phospholipid synthesized from PG or PA, the addition of PG compensated the CL content and also recovered the MMP in VEGF-B–neutralized CD8^+^ T cells (Figure 5E and Supplemental Figure 8O). Moreover, the addition of PA, PG, or CL but no other phospholipids markedly decreased the apoptosis of *Vegfb* cKO CD8^+^ or CD4^+^ T cells (Figure 5G and Supplemental Figure 8P). Therefore, these results suggest that autocrine VEGF-B signaling–dependent CL homeostasis functions as a protective factor against mitochondrial damage in activated T cells.

### Autocrine VEGF-B signaling activates the GABPα/SENP2/PPARγ axis to control phospholipid synthesis in activated T cells.

**Figure 6:**
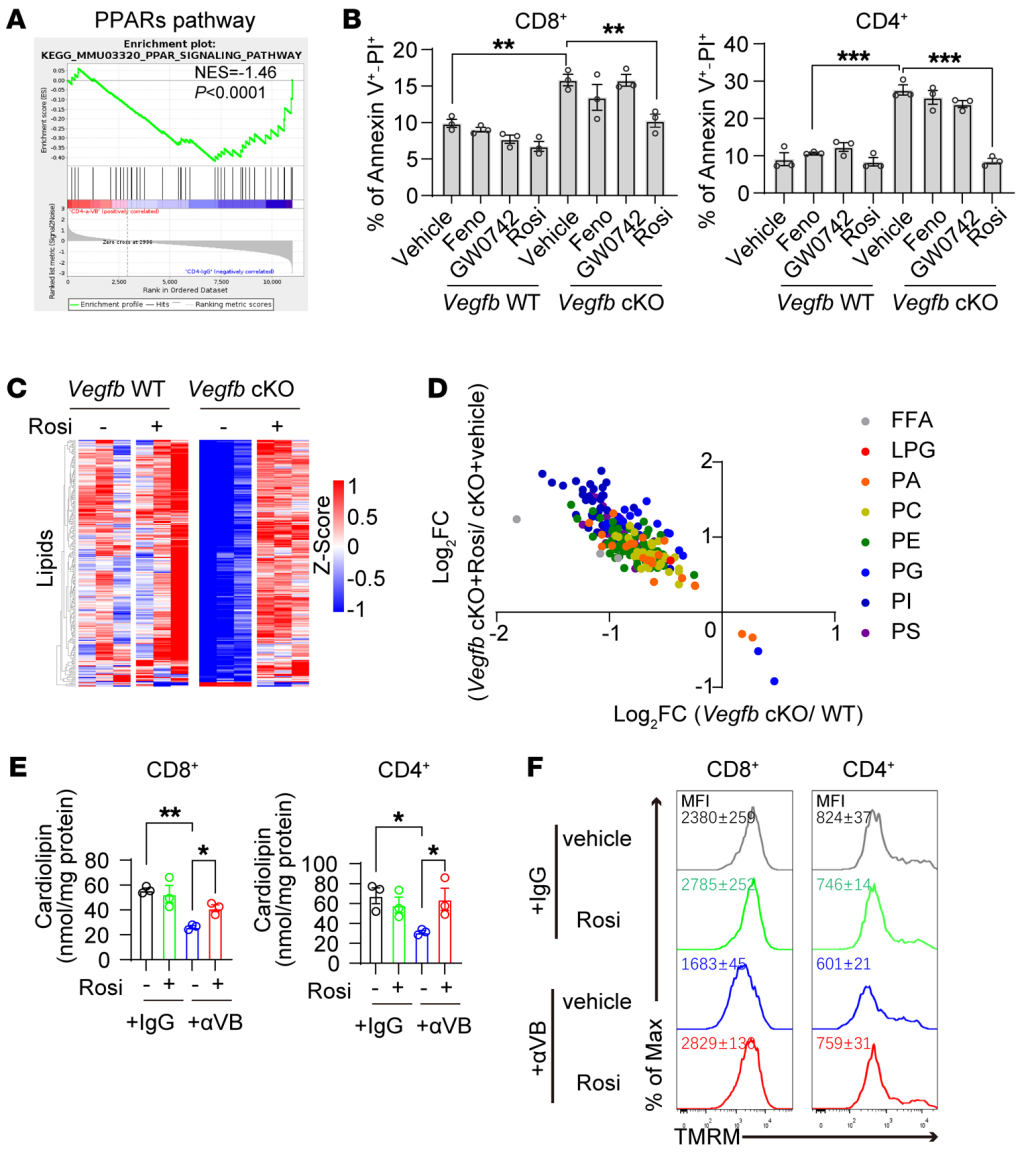
Autocrine VEGF-B maintains lipid synthesis via PPARγ signaling in activated T cells. (**A**) PPAR pathway enrichment in GSEA analysis of RNA-Seq data from αVB-treated CD4^+^ T cells. (**B**) The annexin V^+^ and PI^+^ ratios of T cells with PPAR agonists treatment: fenofibrate (Feno), GW0742, or rosiglitazone (Rosi); *n* = 3. (**C** and **D**) The lipid analysis in CD4^+^ T cells treated with rosiglitazone; *n* = 3. (**E**) Cardiolipin quantification in T cells treated with αVB or IgG plus rosiglitazone; *n* = 3. (**F**) TMRM staining in CD8^+^ (*n* = 4) or CD4^+^ (*n* = 3) T cells treated with αVB or IgG, plus rosiglitazone. Data are shown as mean ± SEM. *P* values were calculated using 2-way ANOVA with Holm-Šídák’s post hoc test in **B** and **E**. *P < 0.05, **P < 0.01, ***P < 0.001.

Since gene profiling demonstrated that PPAR pathway–related genes were downregulated in activated T cells upon VEGF-B neutralization (Figure 6A), we postulated that autocrine VEGF-B might regulate PPAR-controlled phospholipid synthesis (46, 47). As demonstrated, PPARγ agonist rosiglitazone but not the agonists of PPARα or PPARβ/δ reduced apoptosis in activated *Vegfb*-cKO CD8^+^ or CD4^+^ T cells (Figure 6B), suggesting that PPARγ acted as the downstream of autocrine VEGF-B signaling in T cells. Moreover, rosiglitazone treatment increased the content of lipids and CL in *Vegfb*-cKO or VEGF-B–neutralized CD4^+^ or CD8^+^ T cells (Figure 6, C–E). Rosiglitazone treatment also restored the MMP in VEGF-B–neutralized CD8^+^ or CD4^+^ T cells (Figure 6F). In contrast, treatment with the PPARγ antagonist T0070907 reduced the MMP as well as induced apoptosis, which was similar to VEGF-B neutralization treatment in TCR-activated T cells (Supplemental Figure 9, A and B). These results indicate that PPARγ acts downstream of autocrine VEGF-B signaling to facilitate phospholipid synthesis for maintaining mitochondrial fitness in activated T cells.

**Figure 7:**
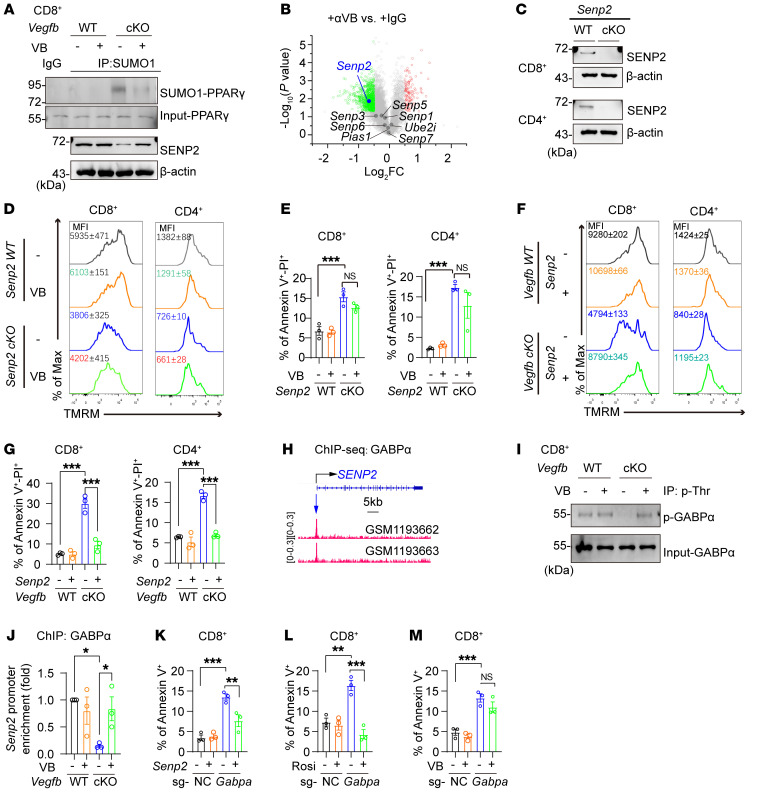
Autocrine VEGF-B maintains lipid synthesis via the GABPα/SENP2/PPARγ axis in activated T cells. (**A**) SUMOylated-PPARγ and SENP2 protein were analyzed in CD8^+^ T cells after addition of VEGF-B; *n* = 3. (**B**) Senp2 expression in RNA-Seq data of CD4^+^ T cells treated with αVB or IgG. (**C**) SENP2 protein levels in *Senp2* WT and cKO T cells; *n* = 3. (**D** and **E**) TMRM and annexin V^+^/PI^+^ staining in *Senp2* WT or cKO T cells after addition of VEGF-B; *n* = 3. (**F** and **G**) TMRM and annexin V^+^/PI^+^ staining in *Vegfb* WT or cKO T cells transduced with *Senp2*; *n* = 3. (**H**) The binding peaks of GABPα on the *SENP2* promoter regions of Jurkat cells from the ChIP-Seq data set (GSE49091). (**I**) The phosphorylation of GABPα in CD8^+^ T cells after addition of VEGF-B. (**J**) ChIP analysis on the binding of GABPα on the *Senp2* promoter region of CD8^+^ T cells; *n* = 3. (**K**) Population of annexin V^+^ cells in sg-NC or sg-*Gabpa* CD8^+^ T cells transduced with *Senp2*; *n* = 3. (**L** and **M**) The population of annexin V^+^ cells in sg-NC or sg-*Gabpa* CD8^+^ T cells with (**L**) rosiglitazone or with (**M**) VEGF-B treatment; *n* = 3. Data are shown as mean ± SEM. *P* values were calculated using 2-way ANOVA with Holm-Šídák’s post hoc test in **E**, **G**, and **J**–**M**. *P < 0.05, **P < 0.01, ***P < 0.001.

Despite the unchanged total and phosphorylated PPARγ protein levels (Supplemental Figure 9C), SUMOylated PPARγ was shown to be accumulated in *Vegfb*-cKO CD8^+^ or CD4^+^ T cells (Figure 7A and Supplemental Figure 9D). Importantly, addition of VEGF-B reduced PPARγ SUMOylation in *Vegfb*-cKO T cells (Figure 7A and Supplemental Figure 9D). Gene profiling showed that the neutralizing of VEGF-B downregulated *Senp2* (sentrin/SUMO-specific protease 2) expression in activated T cells (Figure 7B). The addition of VEGF-B restored SENP2 expression in *Vegfb*-cKO but not in *Flt1*-cKO T cells (Figure 7A, Supplemental Figure 9D, and Supplemental Figure 10, A and B), suggesting that SENP2 was induced by autocrine VEGF-B signaling in T cells. Since SUMOylation prohibits PPARγ activation (46, 48), autocrine VEGF-B–induced SENP2 would activate PPARγ by de-SUMOylation in T cells. To investigate the role of SENP2 in autocrine VEGF-B signaling–activated lipid synthesis in T cells, we further generated *Senp2* conditional knockout (*Senp2-*cKO, *Senp2*^fl/fl^ × *Cd4*^Cre^) mice (Figure 7C). Consistent with *Vegfb* or *Flt1* deficiency, *Senp2* cKO reduced CL content and MMP as well as increased apoptosis in activated CD8^+^ or CD4^+^ T cells (Figure 7, D and E, and Supplemental Figure 10C). The addition of VEGF-B did not recover the CL level, MMP, or survival in *Senp2*-cKO T cells (Figure 7, D and E, and Supplemental Figure 10C). In contrast, overexpression of *Senp2* was capable of rescuing CL content, MMP, and survival in activated *Vegfb*-cKO T cells (Figure 7, F and G, and Supplemental Figure 10D). These data together indicate that autocrine VEGF-B–induced SENP2 expression is essential for the activation of PPARγ by de-SUMOylation in activated T cells.

We further analyzed how VEGF-B induced SENP2 expression in activated T cells. We checked ChIP-Seq data from the Signaling Pathways Project, which illustrated that GA-binding protein α (GABPα) was the transcription factor for *Senp2* expression in T cells (Figure 7H and Supplemental Figure 11, A and B) (49, 50). ERK1/2, a downstream kinase of VEGF-B/VEGFR1 signaling, is reported to activate GABPα by phosphorylation (51–53). We then determined whether autocrine VEGF-B activated GABPα by phosphorylation, leading to *Senp2* transcription. Indeed, *Vegfb*-cKO CD8^+^ or CD4^+^ T cells showed less GABPα phosphorylation than WT cells did (Figure 7I and Supplemental Figure 11C). In contrast, the addition of VEGF-B restored GABPα phosphorylation in *Vegfb*-cKO T cells (Figure 7I and Supplemental Figure 11C). The ChIP assay demonstrated that the binding of GABPα on the *Senp2* promoter was reduced in *Vegfb*- or *Flt1*-cKO T cells (Figure 7J and Supplemental Figure 11D). The addition of VEGF-B restored such binding in *Vegfb*-cKO but not in *Flt1*-cKO T cells (Figure 7J and Supplemental Figure 11D). Importantly, *Gabpa* knockout in CD8^+^ or CD4^+^ T cells reduced the CL content and increased apoptosis upon TCR activation (Figure 7, K–M, and Supplemental Figure 11, E–J). Consistently, either *Senp2* expression or rosiglitazone treatment, but not VEGF-B addition, restored the CL content and survival in *Gabpa*-deficient CD8^+^ or CD4^+^ T cells (Figure 7, K–M, and Supplemental Figure 11, E–J). These data suggest that GABPα-controlled SENP2 expression is essential for autocrine VEGF-B–mediated lipid synthesis and survival in activated T cells.

### Autocrine VEGF-B signaling is essential for the antitumor immunity of CD8^+^ T cells.

**Figure 8:**
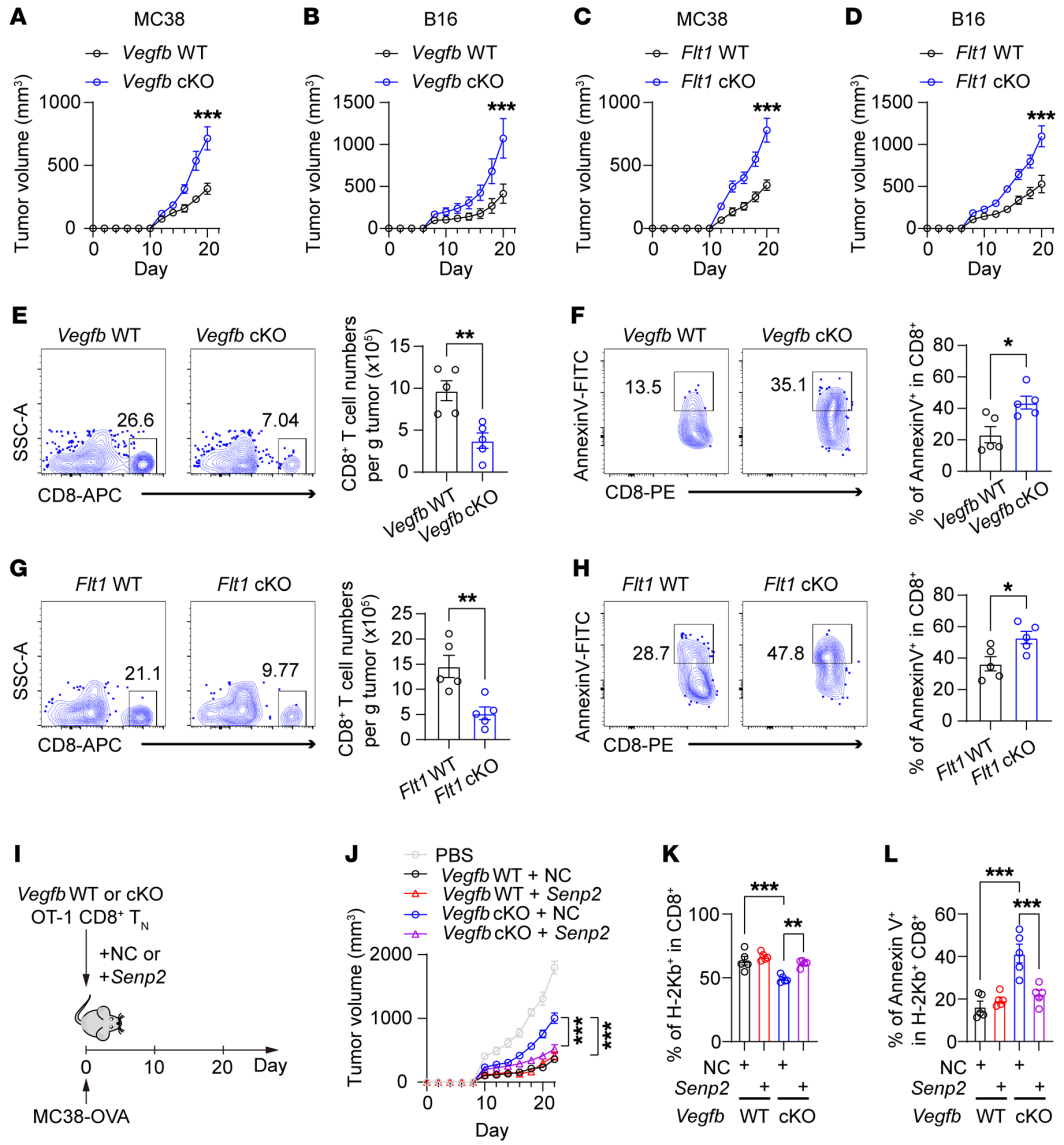
Autocrine VEGF-B signaling is essential for the antitumor immunity of CD8^+^ T cells. (**A**–**D**) The tumor growth curve of MC38 cells or B16 cells on *Vegfb* WT or -cKO or *Flt1* WT or -cKO mice by subcutaneous injection; *n* = 5. (**E** and **F**) Flow cytometry (FCM) analysis for the ratio of tumor-infiltrating CD8^+^ T cells in MC38 tumors on *Vegfb* WT or -cKO mice. (**E**) Cell numbers and (**F**) annexin V^+^ ratio of tumor-infiltrating CD8^+^ T cells were calculated; *n* = 5. (**G** and **H**) FCM analysis for the ratio of tumor-infiltrating CD8^+^ T cells in MC38 tumors on *Flt1* WT or -cKO mice. (**G**) Cell numbers and (**H**) annexin V^+^ ratio of tumor-infiltrating CD8^+^ T cells were calculated; *n* = 5. (**I**) CD8^+^ Tn cells from *Vegfb* WT or -cKO OT-1 cells were activated, followed by *Senp2* overexpression, and then these OT-1 T cells were transferred (i.v.) to recipient mice inoculated with MC38-OVA cells. (**J**) The tumor growth curves were recorded; *n* = 5. (**K**) The H-2Kb^+^ ratio in tumor-infiltrating CD8^+^ T cells and (**L**) the annexin V^+^ ratio of H-2Kb^+^ CD8^+^ T cells were calculated; *n* = 5. Data are shown as mean ± SEM. *P* values were calculated using 2-way ANOVA with Bonferroni’s post hoc test in **A**–**D** and **J**–**L** and 2-tailed unpaired *t* test in **E**–**H**. **P < 0.01, ***P < 0.001.

Since the survival of cytotoxic CD8^+^ T cells was required for antitumor immunity, we examined the effect of autocrine VEGF-B signaling on CD8^+^ T cell–mediated antitumor ability. First, we demonstrated that *Vegfb* or *Flt1* knockout in T cells promoted the growth of the transplantable murine adenocarcinoma MC38 or melanoma B16 tumors (Figure 8, A–D, and Supplemental Figure 12, A–F). Moreover, the number of tumor-infiltrating CD8^+^ T cells in *Vegfb*-cKO mice or *Flt1*-cKO mice decreased with a higher apoptosis level (Figure 8, E–H, and Supplemental Figure 12G). Additionally, the ratio of IFN-γ^+^, TNF-α^+^, or GZMB^+^ in tumor-infiltrating CD8^+^ T cells was decreased in MC38 tumors on *Vegfb*-cKO mice (Supplemental Figure 12, H–J), whereas the cytokine production in these cytokine^+^ cells was not affected. Furthermore, by measuring the ratio and mean fluorescent intensity (MFI) of PD-1, TIM-3, and LAG-3 in tumor-infiltrating CD8^+^ T cells, we found that *Vegfb* deletion did not affect the exhaustion state of CD8^+^ T cells (Supplemental Figure 12K). Consistent with the reduction of tumor-infiltrating CD8^+^ T cells, we found that the number of CD4^+^ cells in MC38 tumors in *Vegfb*-cKO or *Flt1*-cKO mice was decreased, and the ratio of IFN-γ^+^ Th1 and IL-17A^+^ Th17 cells as well as foxp3^+^ Tregs in CD4^+^ T cells were also reduced (Supplemental Figure 12, L–O). In addition, our results showed that *Vegfb* knockout in T cells did not affect the abundance of other immune cells in the MC-38 tumor, including the number of B lymphocytes, natural killer cells, macrophages, myeloid-derived suppressor cells, dendritic cells, and eosinophils (Supplemental Figure 12, P and Q).

We further examined the effect of autocrine VEGF-B signaling on the antitumor effect of antigen-specific CD8^+^ T cells. Antigen-specific CD8^+^ Tn cells from *Vegfb* WT or -cKO OT-1 mice were activated by OVA_257–264_ peptide followed by lentivirus-mediated *Senp2* overexpression, and then these OT-1 T cells were adaptively transferred to recipient mice with MC38-OVA tumor cells inoculated (Figure 8I). The results showed that tumor growth in recipient mice transplanted with *Vegfb*-cKO OT-1 cells was significantly faster than that in recipient mice transplanted with WT OT-1 cells (Figure 8J). In addition, the recipient mice transplanted with *Senp2*-overexpressed *Vegfb*-cKO OT-1 cells had slower tumor growth compared with the negative control mice (Figure 8J). Consistently, we found that *Vegfb* deletion led to a decrease in the proportion of antigen-specific T cells (H-2Kb^+^) infiltrated in tumors with increased levels of apoptosis and decreased levels of CL and MMP, while *Senp2* overexpression restored T cell apoptosis caused by *Vegfb* deletion. (Figure 8, K and L, and Supplemental Figure 13, A–C). Additionally, since PGC1-α serves as the coactivator of PPARγ, we investigated whether overexpressing the *Ppargc1a* gene in *Vegfb*-knockout OT-1 T cells could restore the CD8^+^ T cell deficiency in tumors (Supplemental Figure 13, D and E). We observed that the enhanced expression of the *Ppargc1a* gene had the potential to restore the weakened antitumor ability of CD8^+^ T cells (Supplemental Figure 13F). Additionally, it also reversed the decrease in the population of antigen-specific CD8^+^ T cells, the increase in apoptosis, and the reduction of CL and MMP within these cells caused by the *Vegfb* knockout (Supplemental Figure 13, G–J). Therefore, these results suggest that autocrine VEGF-B signaling is essential for maintaining the antitumor immunity of CD8^+^ T cells.

To investigate the potential significance of VEGF-B in the immunotherapy of human cancer, we analyzed the relationship between the expression level of *VEGFB* and tumor progression in human tumor samples using TCGA data. The results showed that high expression of *VEGFB* was positively correlated with survival probabilities in specific tumors, such as pancreatic ductal adenocarcinoma, ovarian cancer, sarcoma, esophageal squamous cell carcinoma, and others (Supplemental Figure 14A). However, to our surprise, we found that the expression level of *VEGFB* was positively correlated with activated CD8^+^ cell infiltration in most human tumors (Supplemental Figure 14B). It indicates that VEGF-B is a potential driving factor for activated CD8^+^ T cell accumulation in human tumors.

Furthermore, we demonstrated that, consistent with the phenomenon in mice, TCR stimulation induced human CD4^+^ or CD8^+^ T cells to express VEGF-B and its receptor VEGFR-1 (Supplemental Figure 15A), while knockout of *VEGFB* or *FLT1* led to a significant increase in apoptosis levels in human T cells as well as a decrease in CL and MMP (Supplemental Figure 15, B–E). It is also implied that autocrine VEGF-B signaling is involved in regulating lipid homeostasis and promoting cell viability in human T cells, potentially enhancing T cell–mediated antitumor responses.

### Autocrine VEGF-B signaling blocking alleviates T cell–mediated autoimmune diseases.

**Figure 9:**
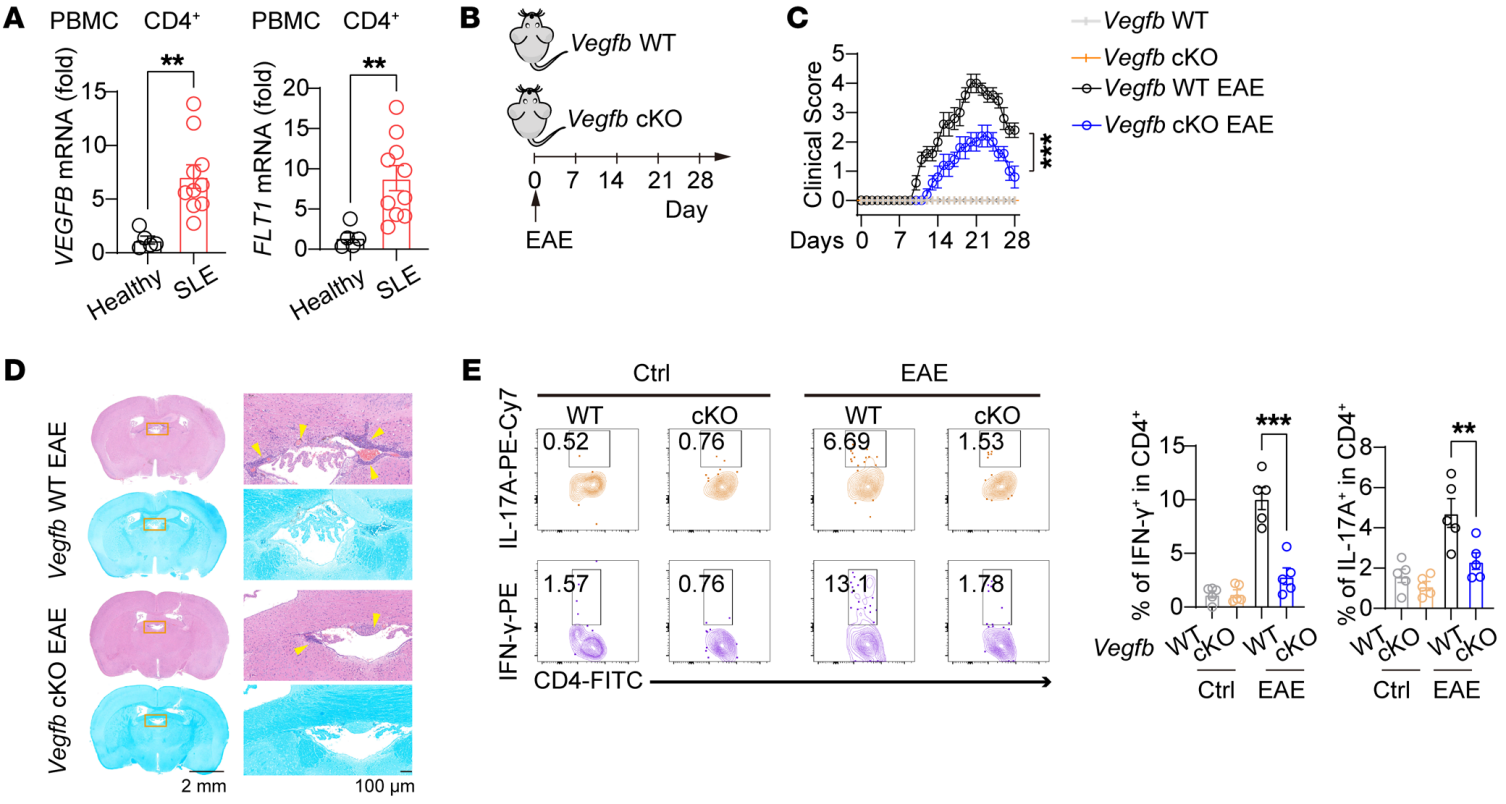
VEGF-B deficiency in T cells alleviates experimental autoimmune encephalomyelitis in mice. (**A**) The relative mRNA levels of *VEGFB* and *FLT1* in CD4^+^ T cells from the PBMCs of patients with SLE (*n* = 10) or healthy donors (*n* = 5). (**B**) The EAE model was generated in *Vegfb* WT or -cKO mice. (**C**) The EAE clinical scores were measured at the indicated days after immunization; *n* = 5. (**D**) Representative H&E and Luxol fast blue (LFB) staining of brain sections from *Vegfb* WT and -cKO EAE mice. The yellow arrows indicate inflammatory infiltration. Scale bar: 2 mm (left); 100 μm (right). (**E**) The percentage of IFN-γ^+^ or IL-17^+^ in CD4^+^ T cells in the brain tissues of mice with or without EAE; *n* = 5. Ctrl, unimmunized control. Data are shown as mean ± SEM. *P* values were calculated using 2-tailed unpaired *t* test in **A** and 2-way ANOVA with Bonferroni’s post hoc test in **C** and **E**. **P < 0.01, ***P < 0.001.

T cells are a player in inflammation-related pathogenesis; we thus determined whether autocrine VEGF-B signaling engaged T cell–mediated autoimmune inflammation. As systemic lupus erythematosus (SLE) is a severe autoimmune inflammatory disease and abnormal activation of CD4^+^ T cells plays predominant roles in the pathogenesis of SLE, we detected the expression of *VEGFB* and *FLT1* in CD4^+^ T cells from patients with SLE. Indeed, CD4^+^ T cells isolated from blood samples of patients with SLE showed an increase in the expression of both *VEGFB* and *FLT1* (Figure 9A and Supplemental Figure 16A). In addition, we examined the database presented by Ota et al. as well as other sources and discovered an elevated level of *VEGFB* or *FLT1* expression within the CD4^+^ or CD8^+^ T cells obtained from individuals with immune-mediated disorders, such as diabetes, asthma, or SLE. (Supplemental Figure 16, B and C) (54). These results suggest that abnormal or excessive activation of T cells in autoimmune disease is associated with high expression of *VEGFB* and its receptor *FLT1*.

**Figure 10:**
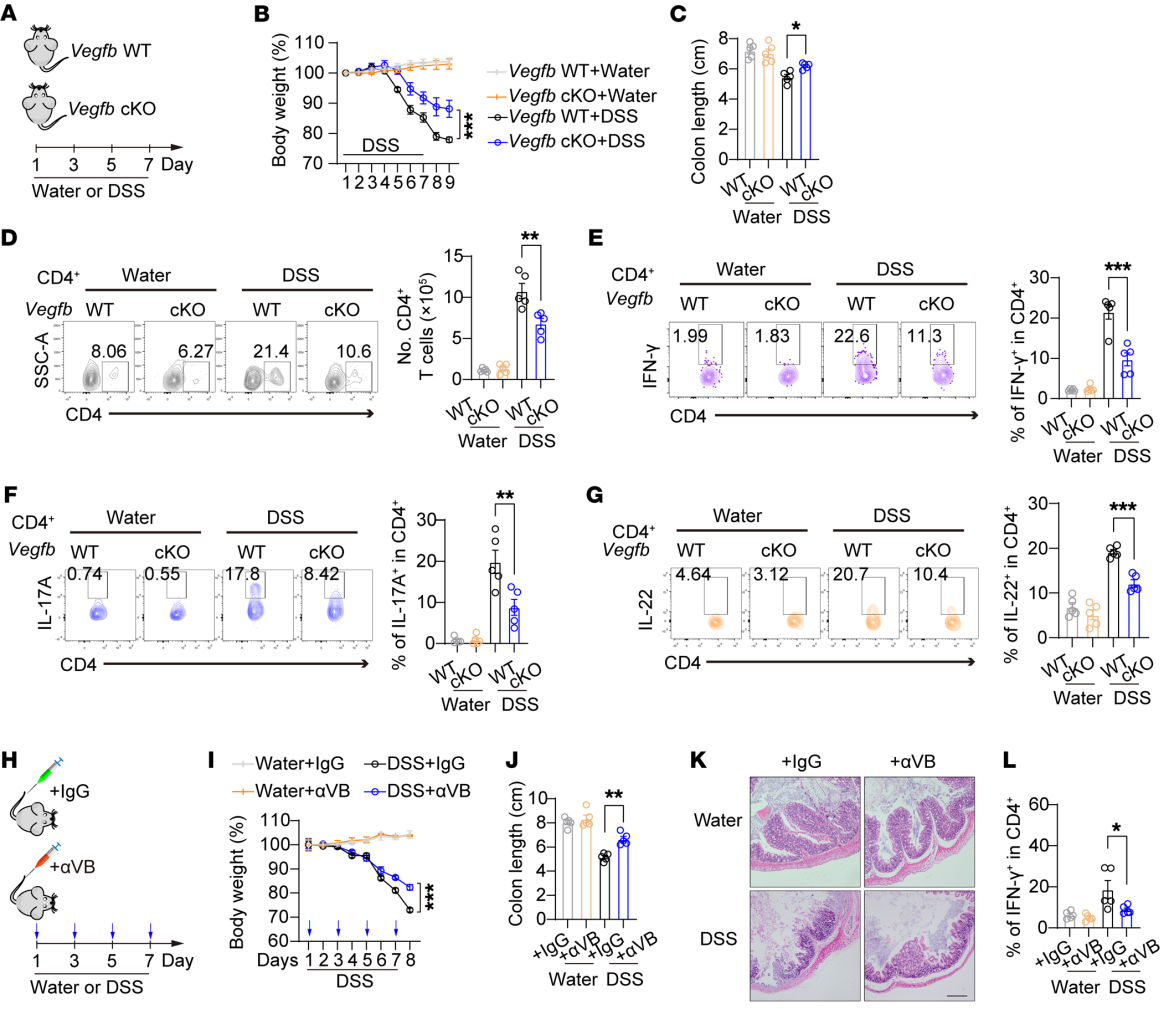
Blocking autocrine VEGF-B signaling prevents DSS-induced colitis in mice. (**A**–**G**) DSS colitis was generated in *Vegfb* WT or -cKO mice. (**B** and **C**) The body weight and the colon length were measured as indicated; *n* = 5. (**D**) The percentage of CD4^+^ T cells in colonic CD45^+^ cells and the total cell number of CD4^+^ T cells in colon tissues of DSS- or water-treated *Vegfb* WT or -cKO mice were calculated; *n* = 5. (**E**–**G**) The percentage of IFN-γ^+^, IL-17A^+^, or IL-22^+^ in CD4^+^ T cells from the colon tissues of DSS- or water-treated *Vegfb* WT or cKO mice; *n* = 5. (**H**–**L**) DSS colitis was induced in mice with i.v. injection of αVB or IgG control (50 μg/kg) every other day; *n* = 5. (**I**) The body weight changes, (**J**) colon lengths, and (**K**) histology of intestinal lesions are shown. Scale bar: 100 μm. (**L**) The population of IFN-γ^+^ in CD4^+^ cells from the colon tissues. Data are shown as mean ± SEM. *P* values were calculated using 2-way ANOVA with Bonferroni’s post hoc test in **B**–**G**, **I**–**J**, and **L**. *P < 0.05, **P < 0.01 and ***P < 0.001.

To explore the role of autocrine VEGF-B signaling in the pathogenesis of T cell–mediated autoimmune diseases, we established a series of mouse models of autoimmune disorders in *Vegfb*-cKO mice. Experimental autoimmune encephalomyelitis (EAE) is an inflammatory demyelinating disease of the CNS with Th1- and/or Th17-driven autoimmunity. We generated an EAE mouse model by immunizing mice with MOG, and we found alleviated CNS inflammation in *Vegfb*-cKO mice as compared with that in WT mice (Figure 9, B–D). IFN-γ^+^ and IL-17A^+^ effector CD4^+^ T cells in brain tissues were markedly reduced in these *Vegfb*-cKO EAE mice (Figure 9E and Supplemental Figure 16D). We also analyzed the DSS-induced colitis mouse model. DSS treatment reduced the body weight and the colon length in both mice as compared with water treatment. However, *Vegfb*-cKO mice showed much milder pathogenic phenotypes than WT control mice after DSS treatment (Figure 10, A–C, and Supplemental Figure 16F). Moreover, the quantities of CD4^+^ T cells and the populations of IFN-γ^+^, IL-17A^+^, or IL-22^+^ in CD4^+^ T cells were lower in the colon tissues of DSS-treated *Vegfb*-cKO mice when compared with DSS-treated WT mice (Figure 10, D–G). Nevertheless, there were no notable variances observed in the MFI of cytokines in IFN-γ^+^, IL-17A^+^, or IL-22^+^ CD4^+^ T cells (Supplemental Figure 16G). Consistent with the ex vivo experimental findings, the absence of *Vegfb* led to a significant decrease in CL and MMP in CD4^+^ T cells in the intestines of the DSS-treated mice (Supplemental Figure 16, H and I). These observations lead us to conclude that autocrine VEGF-B signaling is required for T cell–mediated autoimmune diseases. In addition to the inflammatory Th cells, we also analyzed the Tregs in EAE mice and DSS colitis mice. The results showed that the *Vegfb*-cKO group exhibited a reduced quantity of Foxp3^+^ Tregs compared with the WT group, while the percentage of these cells in CD4^+^ T cells remained relatively stable (Supplemental Figure 16, E and J). These results support our hypothesis that autocrine VEGF-B signaling potentially exerts a broad antiapoptotic effect on different CD4^+^ T cell subtypes. We further examined the therapeutic effects of VEGF-B–neutralizing antibodies on DSS-induced colitis. We demonstrated the protective effect of VEGF-B–neutralizing antibody treatment on body weight loss and colon pathogenic score caused by DSS-induced colitis (Figure 10, H–K). Consistently, the population of IFN-γ^+^CD4^+^ T cells from the colon tissues was markedly reduced in VEGF-B antibody–treated mice as compared with that in control mice (Figure 10L). These data indicate that heightened autocrine VEGF-B signaling in T cells contributes to the development of autoimmune diseases and interventions targeting VEGF-B signaling may offer a potential treatment for autoimmune conditions driven by overactive inflammatory T cells.

## Discussion

In summary, our study demonstrates that lipid synthesis in activated T cells is required for autocrine VEGF-B to maintain mitochondrial fitness against apoptosis. Mechanistically, autocrine VEGF-B signaling activated GABPα to induce SENP2 expression and sequentially activated PPARγ by de-SUMOylation, resulting in the lipid synthesis of activated T cells. Reciprocally, disruption of autocrine VEGF-B signaling damaged mitochondrial architecture and disordered ROS homeostasis, which results in attenuation of T cell–mediated immunity. Like modulating autocrine VEGF-B signaling, the addition of CL, PA, or PG also maintained the survival of activated T cells. These data suggest that autocrine VEGF-B mediates survival signaling by preventing apoptosis through controlling lipid synthesis in activated T cells.

T cells robustly increase ROS production as they activate (37, 55). Interestingly, autocrine VEGF-B is induced in activated T cells via an ROS-dependent manner. A low ROS level exists in the early phase of T cell activation, which has been shown to be an essential factor for T cell proliferation (16, 37, 55–57). Here, we report an activity of ROS that decreased the apoptosis of activated T cells by triggering autocrine VEGF-B signaling. In this sense, this ROS-initiated autocrine VEGF-B signaling provides another way for T cell expansion upon TCR stimulation. However, ROS is often accumulated to a high level in the later phase of T cell activation (57, 58). Such a high dosage of ROS is shown to cause mitochondrial damage or even apoptosis in activated T cells (38–40, 56). Although the molecular mechanisms controlling the expression of VEGF-B in response to ROS level changes remain to be investigated in future studies, our current results strongly suggest that a low dosage of ROS triggers autocrine VEGF-B signaling to mediate a protective mechanism against high-level ROS-caused damage or apoptosis during T cell expansion.

Mitochondria play a crucial role in initiating endogenous apoptosis, and CL in the inner mitochondrial membrane (IMM) acts as an anchor for cytochrome *c*, which can initiate mitochondrial apoptosis (39, 44, 45, 17). Cytochrome *c* is released from the mitochondria into the cytoplasm in response to various injury stimuli or impaired CL biosynthesis (45). This release of cytochrome *c* triggers a cascade of signals that ultimately lead to apoptosis (45, 59). In our study, we have demonstrated that the loss of VEGF-B signaling results in reduced levels of CL and the disruption of the IMM in TCR-activated T cells (Figures 4 and 5). Additionally, we have confirmed that the classical apoptotic pathway, involving the release of mitochondrial cytochrome *c* and the cleavage of caspase-3, is activated in TCR-stimulated T cells with *Vegfb* knockout (Supplemental Figure 5L). Therefore, inhibiting VEGF-B signaling may enhance the release of cytochrome *c* from mitochondria and activate the classical caspase-3 apoptotic pathway by decreasing phospholipid synthesis, especially CL levels, and disrupting the structure of IMM.

While VEGF-B is indispensable for endothelial uptake and transport of fatty acids in the heart and skeletal muscle (36, 42, 43), we demonstrated here that VEGF-B controlled phospholipid synthesis in activated T cells. Autocrine VEGF-B stimulates signaling in activated T cells to promote phospholipid synthesis, which is essential for T cell survival during activation. Disruption of autocrine VEGF-B signaling reduces the expression of genes related to phospholipid synthesis and decreases the content of phospholipids such as PA, PG, and CL, resulting in an increase in apoptosis of TCR-activated T cells. These results reveal a role of VEGF-B in the regulation of phospholipid synthesis in activated T cells. Autocrine VEGF-B–controlled lipid metabolic rewiring increases the survival of activated T cells and is required for the T cell–mediated immune response.

Currently, there are specific concerns that must be addressed in future research to effectively utilize autocrine VEGF-B signaling in T cells for immunotherapy. These limitations and challenges encompass the following aspects. Initially, it is important to consider the temporal and spatial specificity of expression for VEGF-B and its receptor in various T cell subtypes within normal, inflammatory, and tumor tissues in humans. Furthermore, it is necessary to prepare a mouse model with humanized VEGF-B signaling and develop an effective humanized VEGF-B–neutralizing antibody in subsequent studies. In addition, evaluating the potential adverse effects of VEGF-B on human endothelial cells and other cell types is required. In order to minimize the effect of VEGFB-B signaling on other tissues in the human body, the application of autocrine VEGF-B signaling in T cell immunotherapy may primarily focus on two aspects. One is to directly enhance VEGF-B signaling in human chimeric antigen receptor (CAR) T cells for tumor immunotherapy, and the other is to administer VEGF-B–neutralizing antibodies locally in the treatment of T cell–related autoimmune diseases. Despite the numerous challenges that still need to be addressed, on the basis of our current animal trials, autocrine VEGF-B signaling of T cells is expected to be a therapeutic target against infection and tumors and a treatment of autoimmune diseases.

## Methods

### Sex as a biological variable.

Both male and female animals and human samples were examined in this study, and similar findings were reported for both sexes.

### Mice.

C57BL/6 mice purchased from The Jackson Laboratory were described in our previous study (10). MHC class I–restricted OVA-specific TCR OT-1–transgenic mice were obtained from the Shanghai Institute of Immunology, Shanghai Jiao Tong University School of Medicine. The *Vegfb*^fl/fl^ mouse (C57BL/6) was created by using CRISPR/Cas9 technology to insert loxP sites into exon 2–6 of the *Vegfb* gene. The *Flt1*^fl/fl^ mouse (C57BL/6) was created by using CRISPR/Cas9 technology to insert loxP sites into exon 4 of *Flt1* gene. T cell–specific *Vegfb-*cKO (*Vegfb*^fl/fl^ × *Cd4*^Cre^) or *Flt1-*cKO (*Flt1*^fl/fl^ × *Cd4*^Cre^) mice were generated by crossing a *Vegfb*^fl/fl^ mouse or a *Flt1*^fl/fl^ mouse with a *Cd4*^Cre^ mouse (The Jackson Laboratory), respectively. The T cell–specific *Senp2-*cKO mouse was generated by a *Senp2*^fl/fl^ mouse crossed with a *Cd4*^Cre^ mouse. The *Senp2*^fl/fl^ mouse was created by homologous recombination in a previous study (60). All mice were bred and maintained under specific pathogen–free conditions.

### Human blood samples.

Whole blood samples were donated by patients with SLE and healthy volunteers to the Department of Rheumatology, Shanghai Jiao Tong University School of Medicine, which is affiliated with Renji Hospital. Ten donors with SLE were analyzed, and the clinical features, including age and sex, of all donors in this study are detailed in Supplemental Table 1. PBMCs were isolated by a Ficoll-Paque (GE Healthcare) density gradient centrifugation. CD4^+^ T cells in PBMCs were purified with EasySep Human CD4 T Cell Iso Kits (17952, Stemcell). See Supplemental Methods for details on the T cell treatment in vitro and mitochondrial experiments. The catalog numbers and sources of cytokines and antibodies are shown in Supplemental Table 3.

### Listeria monocytogenes infection.

A recombinant strain of LM-OVA was obtained from Hao Shen’s laboratory (Department of Microbiology, School of Medicine, University of Pennsylvania, Philadelphia, Pennsylvania, USA) (61). LM-OVA were grown in Brain Heart Infusion Medium until the early log phase, and their growth was assessed with a photometer at OD_600_. For OT-1 CD8^+^ T cell activation in vivo, the recipient mice were adoptive transferred with naive CD8^+^ OT-1 T cells (1.0 × 10^4^ cells) and i.v. infected with LM-OVA (5.0 × 10^3^ CFU). OVA-specific CD8^+^ T cells (tetramer H-2Kb^+^) in the spleens of infected mice were assessed after infection. The spleen and liver of the recipient were homogenized for *Listeria monocytogenes* CFU counting with gradient dilution and cultured at 37°C for 18 hours on plates without antibiotics.

### EAE induction.

Six- to 8-week-old *Vegfb* WT and cKO female mice were used in the EAE model. EAE was induced by subcutaneous immunization with MOG_35–55_ peptides (200 μg/mouse, catalog T510219, Sangon Biotech) emulsified in complete Freund’s adjuvant (catalog F5881, Sigma-Aldrich), followed by administration of pertussis toxin (200 ng/mouse, PTX, List Biological Laboratories Inc.) on days 0 and 2 as described above. Clinical signs of EAE were assessed according to the following score: 0, no signs of disease; 1, loss of tone in the tail; 2, hind limb paresis; 3, hind limb paralysis; 4, tetraplegia; 5, moribund. H&E and Luxol fast blue staining in the brain sections were used to detect the inflammatory infiltration and demyelination of *Vegfb* WT and -cKO EAE mice.

### DSS-induced acute colitis.

Six- to 8-week-old *Vegfb* WT and cKO female mice were used in the acute colitis model. Acute colitis was induced by feeding mice with 2.5% (w/v) dextran sulfate sodium (DSS) (16011080, MP Biomedicals) in drinking water. Mice were given DSS in drinking water for 7 days. Subsequently, DSS was replaced by normal drinking water for 2 days. The induction of colitis was determined by measuring weight loss, colon length, and histological analysis.

### Tumor studies.

For the tumor models, 6- to 8-week-old male mice were injected subcutaneously with 2 × 10^5^ tumor cells and then monitored every other day for tumor growth. For antitumor immunity assays, the OVA_257–264_ activated *Vegfb* WT or -cKO CD8^+^ OT-1 T cells (H-2Kb^+^) with *Senp2* gene overexpressed (1 × 10^6^) were transferred i.v. into congenic recipient mice after tumor implanted. For the analysis of tumor-infiltrating CD8^+^ T cells, MC38 or B16 tumors were digested using collagenase IV (100 U/mL) and deoxyribonuclease I (DNase I; 50 U/mL) in complete RPMI 1640 medium at 37°C for 30–60 minutes. The single-cell suspensions of tumor tissues were then stained and analyzed according to subsequent flow cytometry staining methods.

### Transmission electron microscopy.

T cells were fixed in 2.5% glutaraldehyde for ultrathin sectioning, and the cut sections were imaged using a HITACHI H-7650 or PHILIPS CM-120 transmission electron microscope. RADIUS 2.0 software (EMSIS GmbH) was used for transmission electron microscope data collection at the Core Facility of Basic Medical Sciences, Shanghai Jiao Tong University School of Medicine. ImageJ (NIH, 1.52a) and Image Pro-Plus (version 6.0) were used to analyze and process the electron microscopy images.

### Cardiolipin quantification.

The CL level was measured by fluorometric assay kits (K944, BioVision) according to the kit instructions. The CL mass was stained by using the green fluorescent probe NAO (λEx/λEm = 495/522 nm, MX4301, Maokangbio) and analyzed by flow cytometry instrumentation.

### Quantitative real-time PCR.

RNAs were isolated with TRIzol reagent (DP424, TIANGEN), and the cDNAs were generated by a reverse transcription kit according to the manufacturer’s instructions (RR036, Takara). Transcript expression was determined by quantitative real-time PCR with FastStart Universal SYBR Green Master (04913914001, Roche). All quantitative real-time PCR reactions were carried out using the Light Cycler 480 Instrument or Light Cycler 96 Instrument (Roche). *Actb* (β-Actin) was used as a control for quantification. Primers are listed in Supplemental Table 2.

### Bulk RNA-Seq of T cells.

CD4^+^ Tn cells were activated by anti-CD3 (5 μg/mL) and anti-CD28 (2 μg/mL) plus IL-2 (100 U/mL) with the treatment of anti–VEGF-B (1 μg/mL; AF-590, R&D Systems) or normal goat IgG control (1 μg/mL; AB-108-C, R&D Systems) for 3 days. Then, 5 × 10^5^ cells were pelleted to extract total RNA by TRIzol reagent. The KAPA Stranded RNA-Seq Library Prep Kit (Illumina) was used for cDNA construction. The TruSeq SR Cluster Kit v3-cBot-HS (GD-401-3001, Illumina) and Illumina HiSeq 4000 were used for sequencing. Sequenced reads were aligned to the mouse genome (GRCm38/mm10) with hisat2 (2.1.0) and StringTie (1.3.3). Transcript expression levels were analyzed by Ballgown (2.10.0) and CPAT (1.2.4). Raw counts were calculated, and differentially expressed genes were identified with R (3.5.0) and Python (2.7). The KEGG database and GSEA (2.2.4) were used for subsequent cellular and molecular pathway analysis.

### ELISA.

VEGF-B protein was analyzed by ELISA using the Mouse VEGF-B ELISA Kit (KOA0852, Rockland Immunochemicals Inc.) according to the manufacturer’s instructions.

### Statistics.

Comparisons for 2 groups were calculated using unpaired 2-tailed Student’s *t* tests; comparisons for more than 2 groups were calculated using 1-way or 2-way ANOVA, followed by the Holm-Šídák, Bonferroni’s, or 2-stage Benjamini, Krieger, and Yekutieli false discovery rate procedures for multiple-comparison tests, as indicated in figure legends. Representative results of at least 3 independent experiments are shown. The statistical tests and sample size “*n*” are indicated in each figure. The SEM and *P* values were determined using GraphPad Prism (V9.0.0). *P* values of less than 0.05 were considered statistically significant.

### Study approval.

All animal experiments were performed in accordance with the Guide for the Care and Use of Laboratory Animals (National Academies Press, 2011) and were approved by the Experimental Animal Ethical Committee at Shanghai Jiao Tong University School of Medicine. The collection and testing of human blood samples (patients with SLE and healthy volunteers) were approved by the Ethics Committee of Renji Hospital, Shanghai Jiao Tong University (KY2022-023-B). Written informed consent was received prior to the use of the human samples.

### Data availability.

Raw RNA-seq data were deposited into the NCBI Sequence Archive (accession GSE264040). ChIP-Seq data used here are from the NCBI as well (accession GSE49091). All the data needed to evaluate the conclusions in the paper are present in the paper or the supplemental materials. Data from experiments and analyses used to generate the figures can be found in the Supporting Data Values file.

## Author contributions

JH, TW, and JC designed and conceived the research. JH, YC, HD, JAZ, and TW developed experimental methods, performed most of the experiments, and analyzed data. XY, JAZ, ZX, YZ, and QF provided technical support. JH, TW, and JC wrote the manuscript and supervised the work.

## Supplementary Material

Supplemental data

Unedited blot and gel images

Supporting data values

## Version 1 08/15/2024

Electronic publication
