# Supplementary material for: Autocrine VEGF-B signaling maintains lipid synthesis and mitochondrial fitness to support T cell immune responses
Source: J Clin Invest. 2024 Aug 15;134(16):e176586. doi: 10.1172/JCI176586 (PMC11324299; doi:10.1172/JCI176586)
Supplement: Supplemental data [file jci-134-176586-s198.pdf]

Supplementary Materials for

Autocrine VEGF-B Signaling Maintains Lipid Synthesis and Mitochondrial Fitness to Support T Cell Immune Responses

Supplemental Figures and legends (Supplemental Figure 1 to 16)

Supplemental Methods

Supplemental Tables (Table S1 to S3)

References (1–14)

Supplemental Figures and legends:

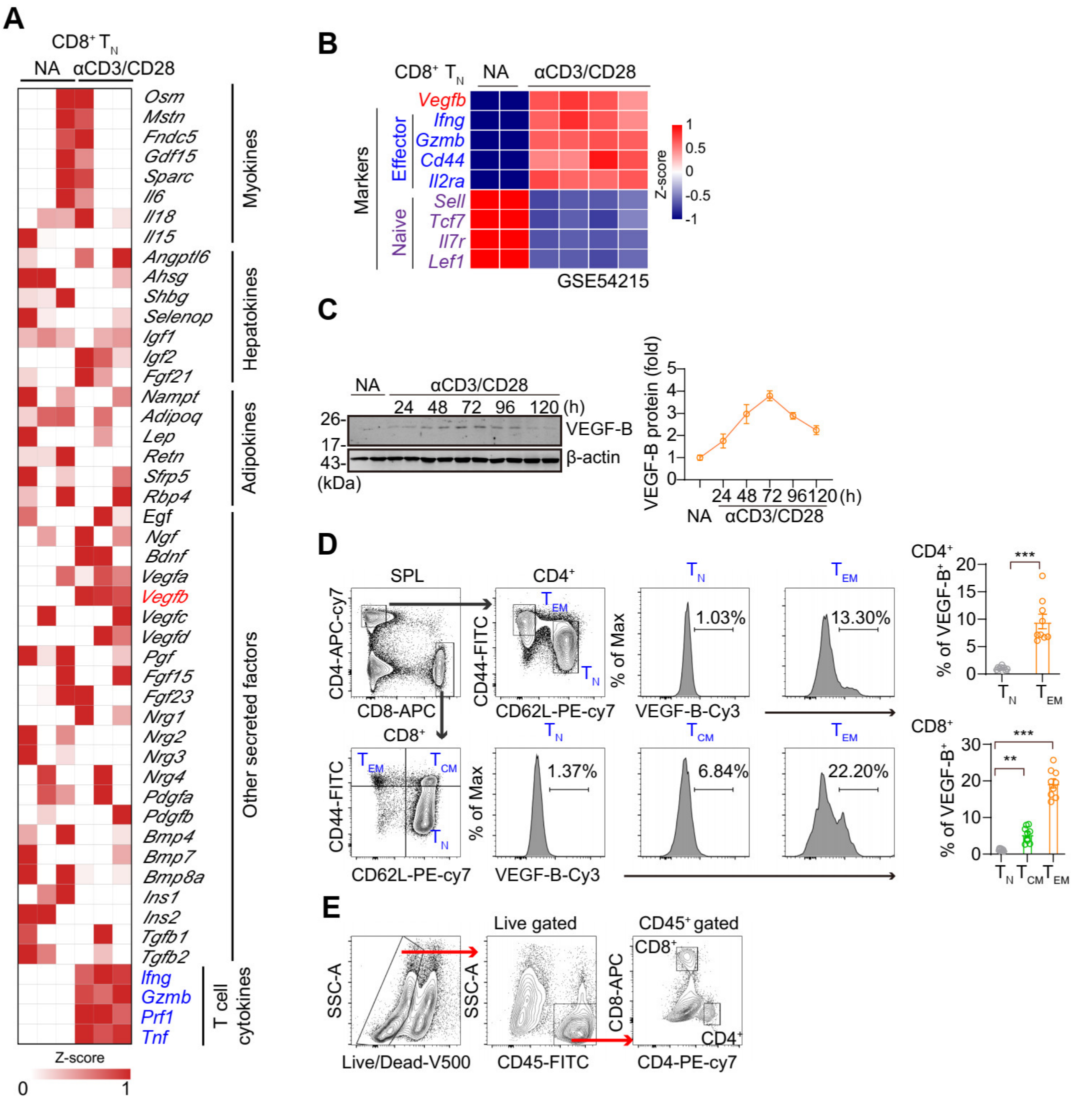

Supplemental Figure 1. The *Vegfb* expression in TCR-activated T cells.
(A) Heatmap for relative mRNA levels of the secreted factors in CD8$^+$ T$_N$ treated with αCD3/CD28 stimulation for 3 days or without treatment (NA), n = 3. The expression of *Prf1*, *Ifng*, *Gzmb*, and *Tnf* was used as a positive indicator for T cell activation. **(B)** Heatmap for the expression of *Vegfb* and marker genes for naïve and effector CD8$^+$ T cells with αCD3/CD28 stimulation for 3 days from the GEO database (GSE54215) (1). **(C)** VEGF-B protein was blotted by anti-VEGF-B antibody in non-activated (NA) or αCD3/CD28 activated CD8$^+$ T$_N$ cells at 24, 48, 72, 96, and 120 hours, n = 6. **(D)** Flow cytometric analysis of the population of VEGF-B positive (VEGF-B$^+$) cells in CD4$^+$ and CD8$^+$ naïve (T$_N$, CD44$^-$ CD62L$^+$), central memory (T$_{CM}$, CD44$^+$ CD62L$^+$), or effector memory (T$_{EM}$, CD44$^+$ CD62L$^-$) T cells isolated from the spleen of normal C57BL/6 mice, n = 9. **(E)** Flow cytometric gating strategy for CD4$^+$ and CD8$^+$ T cells in the colon of acute DSS-induced colitis mice. Data are shown as mean ± SEM. P values were calculated using two-tailed unpaired t-test for CD4$^+$ T cells, and one-way ANOVA with Bonferroni's post-hoc test for CD8$^+$ T cells in **(D)**. $^{**}P < 0.01$ and $^{***}P <$ 0.001.

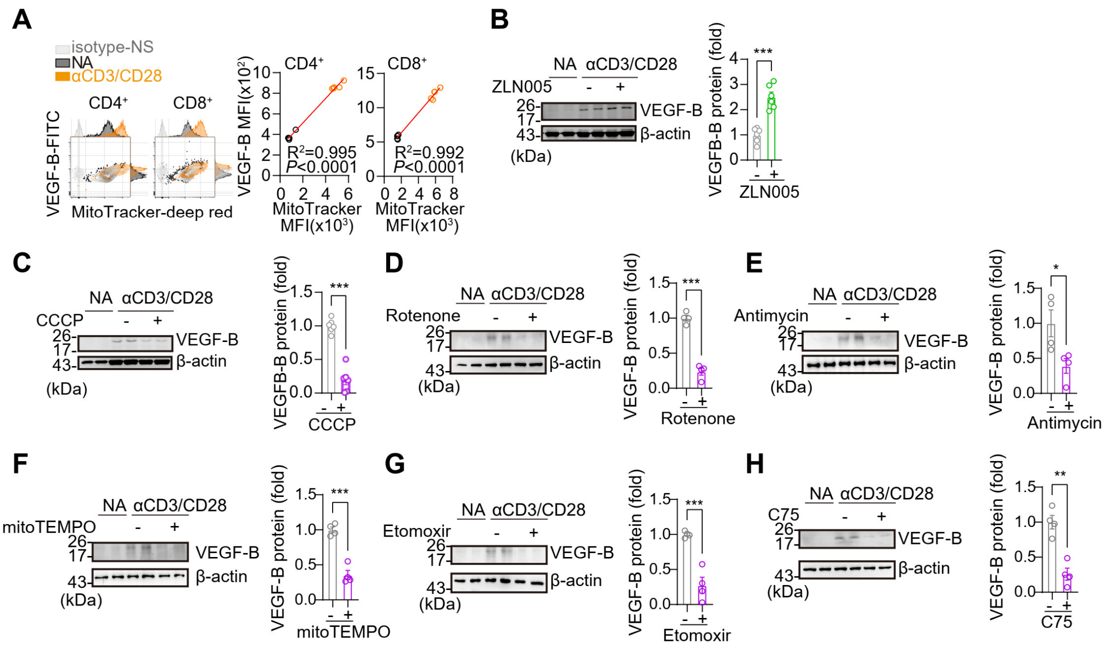

Supplemental Figure 2. VEGF-B expression correlates with mitochondrial activity during T cell activation.

(A) The co-staining of VEGF-B protein and mitochondrial mass (Mitotracker deep red) in naïve and activated $CD4^+$ or $CD8^+$ T cells was detected by flow cytometry, and the Pearson correlations of VEGF-B and MitoTracker MFI were calculated (R squared and two-tailed *P* value), *n* = 5. (**B**) VEGF-B protein was blotted by anti-VEGF-B antibody in αCD3/CD28-activated $CD8^+$ T cells with or without PGC-1α activator ZLN005 treatment, *n* = 6. (**C-H**) VEGF-B protein was blotted by anti-VEGF-B antibody in αCD3/CD28 activated $CD8^+$ T cells with the treatment of CCCP (*n* = 6), Rotenone (*n* = 4), Antimycin (*n* = 4), mitoTEMPO (*n* = 4), Etomoxir (*n* = 4), or C75 (*n* = 4). Data are shown as mean ± SEM. *P* values were calculated using two-tailed unpaired t-test in **(B)** to **(H)**. *$P < 0.05$, **$P < 0.01$ and ***$P < 0.001$.

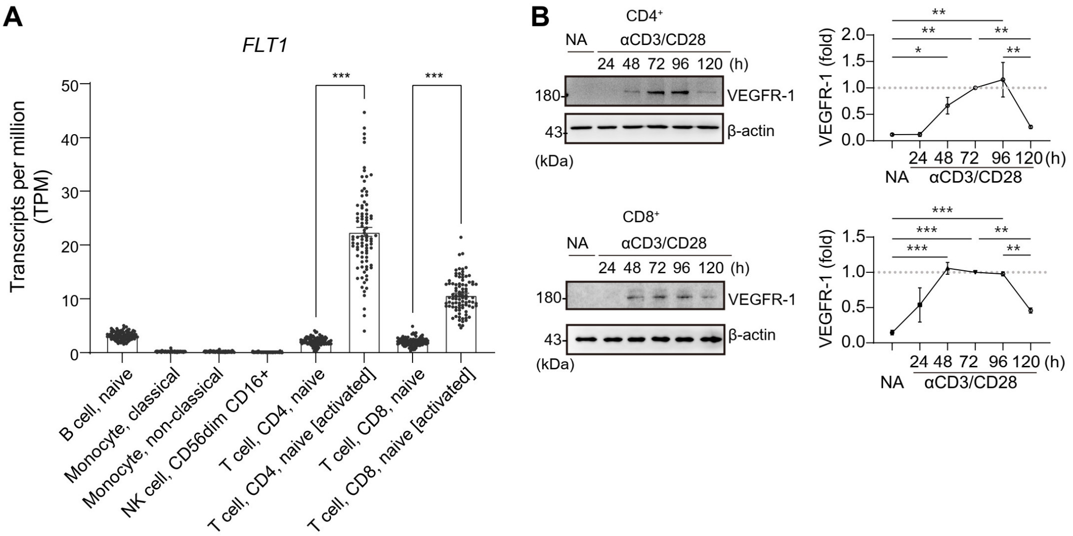

Supplemental Figure 3. VEGFR-1 expression in TCR-activated T cells.

(A) *FLT1* gene expression in human immune cells from the DICE (Database of Immune Cell Expression, Expression quantitative trait loci (eQTLs) and Epigenomics) project (2, 3). **(B)** VEGFR-1 protein was blotted by anti-VEGF-B antibody in non-activated (NA) or αCD3/CD28 activated $CD8^+$ or $CD4^+$ T_N cells at 24, 48, 72, 96, and 120 hours, and the relative levels of VEGFR-1 protein fold were normalized to samples at 72 hours after αCD3/CD28 activation, $n = 3$. P values were calculated using two-way ANOVA with Benjamini, Krieger, and Yekutieli post-hoc test in (**A** and **B**). $^*P < 0.05$, $^{**}P < 0.01$ and $^{***}P < 0.001$.

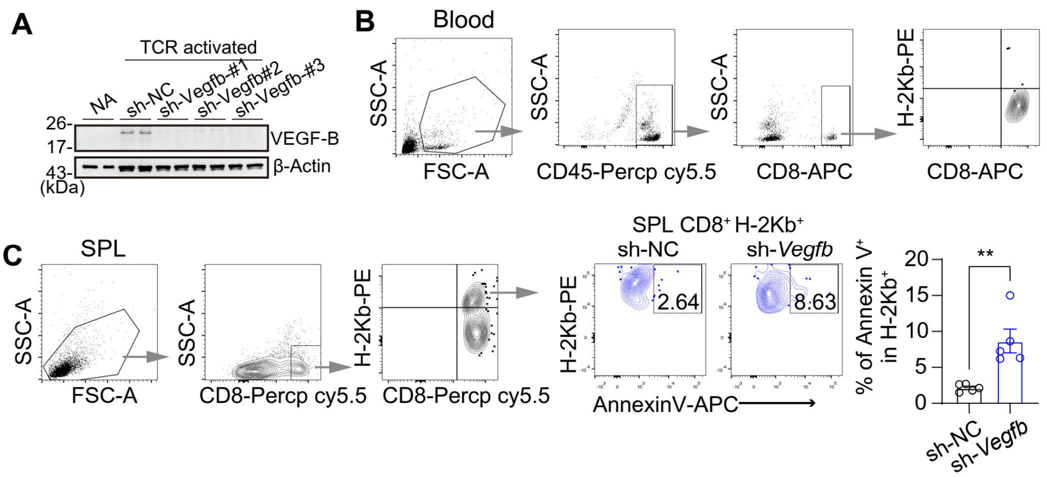

Supplemental Figure 4. The knockdown of *Vegfb* through lentivirus in activated $CD8^+$ T cells.

(A) VEGF-B protein was blotted by anti-VEGF-B antibody in non-activated or αCD3/CD28 activated $CD8^+$ T cells infected by sh-RNA lentivirus with negative control (sh-NC) or VEGF-B knockdown (sh-*Vegfb*-#1, sh-*Vegfb*-#2, sh-*Vegfb*-#3), $n = 3$. **(B)** Flow cytometric gating for the percentage of tetramer H-2Kb$^+$ in $CD8^+$ T cells from the blood of recipient mice at the indicated days during LM-OVA infection. **(C)** Flow cytometric gating for the percentage of H-2Kb$^+$ in $CD8^+$ T cells and the Annexin V^+ ratio in H-2Kb$^+$ $CD8^+$ T cells from the spleen of recipient mice with LM-OVA infection. The Annexin V^+ ratio in H-2Kb$^+$ $CD8^+$ T cells in the spleen of LM-OVA infected recipients was calculated, $n = 5$. Data are shown as mean ± SEM. P values were calculated using two-tailed unpaired t-test in **(C)**, $^{**}P < 0.01$.

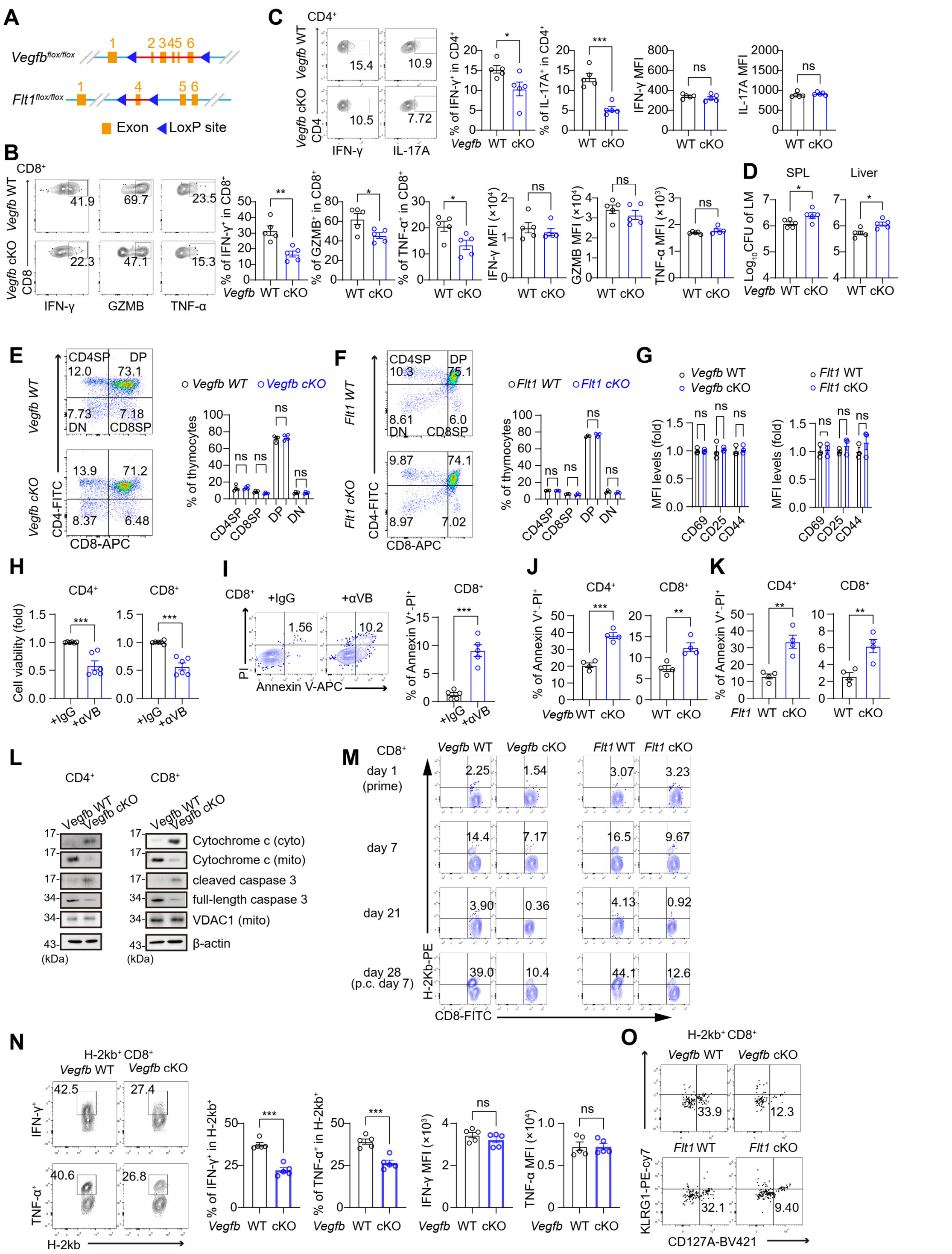

Supplemental Figure 5. Deficiency of *Vegfb* or *Flt1* facilitated the apoptosis of activated T cells.

(A) Construction strategies for *Vegfb*$^{flox/flox}$ mice and *Flt1*$^{flox/flox}$ mice. **(B-D)** *Vegfb* WT and cKO mice were infected with Listeria monocytogenes (LM) for 7 days. The percentages of IFN-γ$^+$, GZMB$^+$, or TNF-α$^+$ effector cells in CD8$^+$ T cells from the spleen of the infected recipient were detected, and the MFI of IFN-γ in IFN-γ$^+$ CD8$^+$ T cells, GZMB in GZMB$^+$ CD8$^+$ T cells, and TNF-α in TNF-α$^+$ CD8$^+$ T cells were calculated, *n* = 5 **(B)**. The percentages of IFN-γ$^+$ or IL-17A$^+$ effector cells in CD4$^+$ T cells were detected, and the MFI of IFN-γ in IFN-γ$^+$ CD4$^+$ T cells and IL-17A in IL-

17A$^+$ CD4$^+$ T cells were calculated, n = 5 **(C)**. The CFU of LM in the SPL and the liver of the infected recipient were calculated, n = 5 **(D)**. **(E-F)** Flow cytometric analysis of the percentages of CD4/CD8 double negative (DN), CD4/CD8 double positive (DP), and CD4 or CD8 single positive (SP) cells in the thymus of *Vegfb* WT and cKO (n = 4) or *Flt1* WT and cKO mice (n = 3). **(G)** The relative MFI levels of CD69, CD25, and CD44 in *Vegfb* WT and cKO, or *Flt1* WT and cKO CD8$^+$ T_N cells with TCR activation for 3 days, n = 3. **(H)** The cell viability of TCR-stimulated CD4$^+$ or CD8$^+$ T cells treated by IgG or anti-VEGF-B (αVB) antibody, n = 6. **(I)** The Annexin V$^+$ and PI$^+$ ratio in CD8$^+$ T cells treated with IgG or αVB, n = 5. **(J-K)** Flow cytometric analysis of the population of Annexin V$^+$ and PI$^+$ in TCR-stimulated CD4$^+$ or CD8$^+$ T cells isolated from *Vegfb* WT and cKO, n = 4 **(J)**, or *Flt1* WT and *Flt1* cKO mice, n = 4 **(K)**. **(L)** The protein levels of Cytochrome C in the cytosol (cyto) or mitochondrial (mito) and the protein levels of the cleaved and full-length caspase-3 in *Vegfb* WT and cKO CD4$^+$ or CD8$^+$ T cells. VDAC1 was used as an internal control for total mitochondrial protein. A representative experiment of three independent replicates is shown. **(M)** Flow cytometric analysis of the percentage of tetramer H-2Kb$^+$ in CD8$^+$ T cells from the blood of recipient mice at the indicated days during infection, n = 5. **(N)** *Vegfb* WT and cKO CD8$^+$ OT-1 T cells were adoptively transferred to recipient mice, followed by infection with LM-OVA for 7 days. The percentage of IFN-γ$^+$ or TNF-α$^+$ cells in H-2Kb$^+$ CD8$^+$ T cells from the spleen of the recipient. The IFN-γ MFI in IFN-γ$^+$ H-2Kb$^+$ CD8$^+$ T cells and the TNF-α MFI in TNF-α$^+$ H-2Kb$^+$ CD8$^+$ T cells were calculated, n = 5. **(O)** Flow cytometric analysis of the percentage of memory CD8$^+$ T cells (KLRG1$^-$ CD127A$^+$) in H-2Kb$^+$ CD8$^+$ T cells from the spleen from recipient mice on day 7 of prime infection, n = 5. Data are shown as mean ± SEM. P values were calculated using two-tailed unpaired t-test in **(B)** to **(K)** and **(N)**. ns: not significant, *P < 0.05, **P < 0.01 and ***P < 0.001.

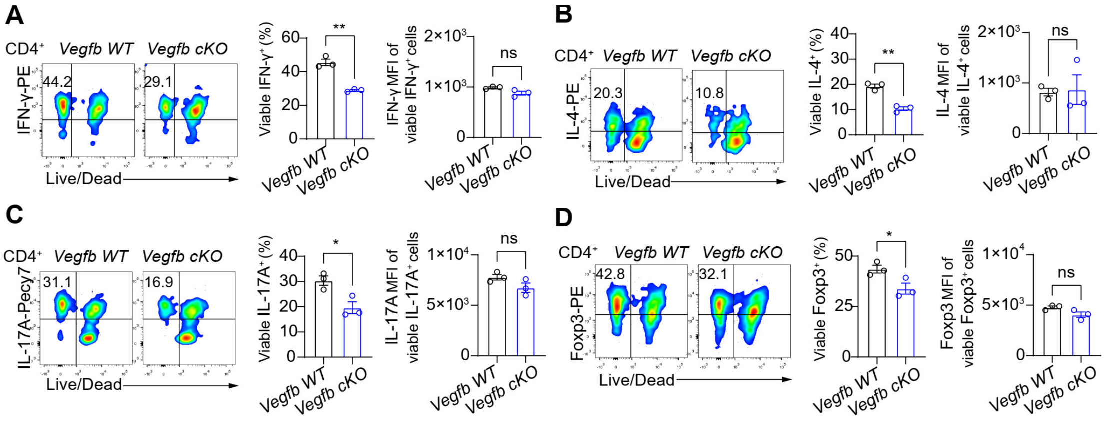

Supplemental Figure 6. The effect of *Vegfb* deficiency on the differentiation of CD4$^+$ T helper cells.

(A-D) CD4$^+$ naïve T cells from *Vegfb* WT or cKO mice were activated by αCD3/CD28 + IL-2 for 3 days, and then cultured under polarization conditions for 3 days to induce Th1, Th2, Th17, or Treg cells ex vivo. The percentages of viable IFN-γ$^+$ Th1 cells, IL-4$^+$ Th2 cells, IL-17A$^+$ Th17 cells, and Foxp3$^+$ Treg cells in CD4$^+$ T cells were detected, and the MFI of IFN-γ in viable Th1 cells, IL-4 in viable Th2 cells, IL-17A in viable

Th17 cells, and Foxp3 in viable Treg cells were calculated, $n = 3$. Data are shown as mean ± SEM. P values were calculated using two-tailed unpaired t-test in **(A)** to **(D)**. ns: not significant, *$P < 0.05$ and **$P < 0.01$.

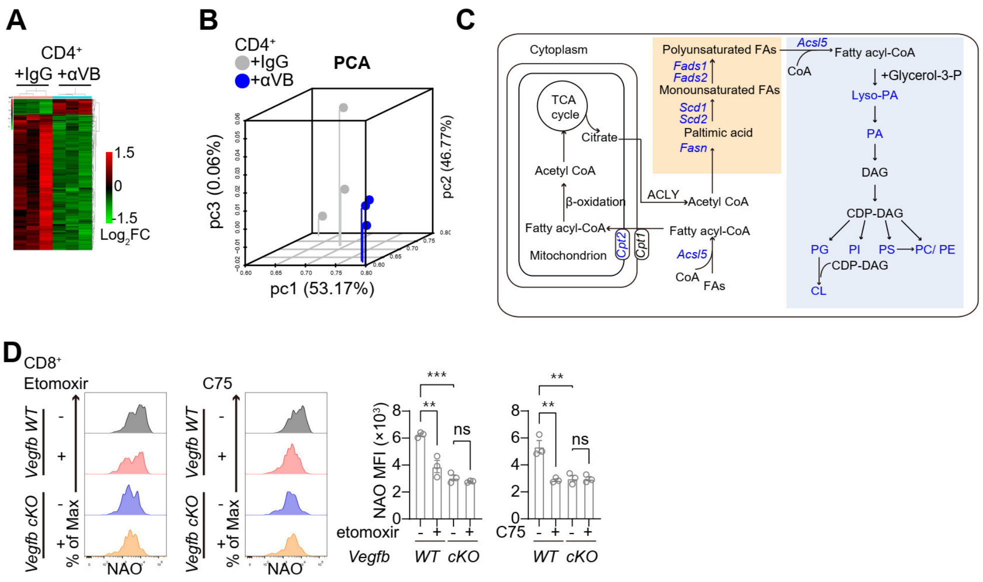

Supplemental Figure 7. VEGF-B regulates the lipid metabolism in activated CD4$^+$ T cells.

(A-C) RNA-seq data from TCR-stimulated CD4$^+$ T cells treated with VEGF-B neutralization antibody (αVB, 1 μg/mL) or IgG, $n = 3$. The heat map of differentially expressed genes **(A)** and PCA analysis **(B)** were shown. **(C)** Schematic diagram of the down-regulated fatty acid metabolism genes and lipids (blue font) in the fatty acid metabolism pathway. **(D)** The cardiolipin levels with NAO staining in *Vegfb* WT and cKO CD8$^+$ T cells with etomoxir or C75 treatment were measured, $n = 3$. Data are shown as mean ± SEM. P values were calculated using two-way ANOVA with Bonferroni's post-hoc test in **(D)**. ns: not significant, **$P < 0.01$ and ***$P < 0.001$.

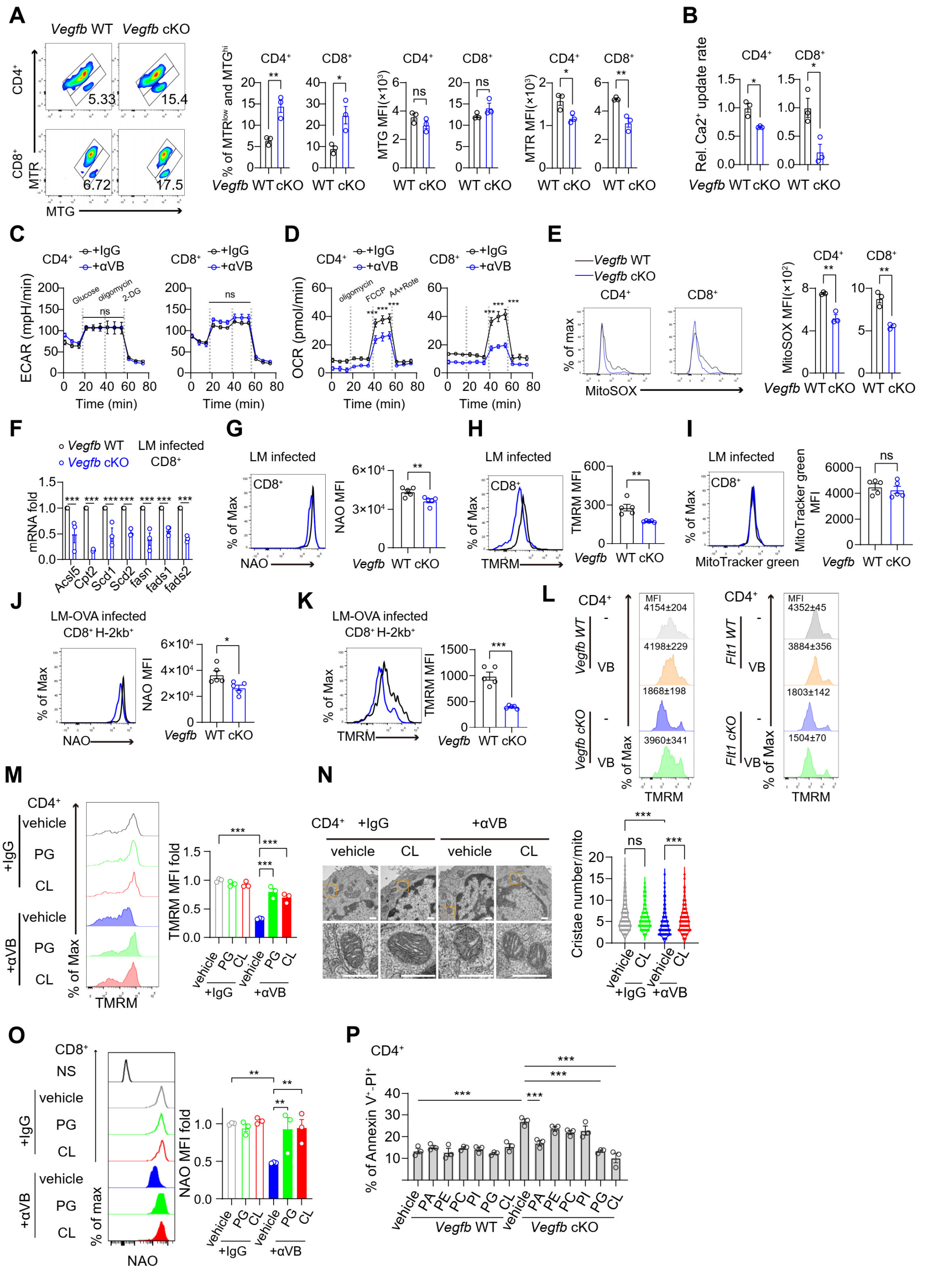

Supplemental Figure 8. VEGF-B is required to maintain mitochondrial function in activated T cells.

(**A**) The total mitochondrial mass and mitochondrial membrane potential (MMP) of TCR-stimulated *Vegfb* WT and cKO CD4$^+$ or CD8$^+$ cells were detected by MitoTracker Green (MTG, MMP-insensitive dye) and MitoTracker Red (MTR, MMP-sensitive dye) staining. The percentages of MTR^{low} and MTG^{high}, the MFI of MTG, and the MFI of MTR were calculated, n = 3. **(B)** The relative mitochondrial Ca2$^+$ uptake rate of TCR-stimulated *Vegfb* WT and cKO CD4$^+$ or CD8$^+$ T cells was quantified by the mitochondrial Ca2$^+$ uptake assay, n = 3. **(C-D)** The metabolic flux assay by the Seahorse

bioanalyzer was used to measure the ECAR (mpH/min) and the OCR (pmol/min) of TCR-stimulated $CD4^+$ or $CD8^+$ T cells with IgG or VEGF-B neutralization antibody (αVB) treatment, *n* = 3. **(E)** Mitochondrial ROS levels of TCR-stimulated *Vegfb* WT and cKO $CD4^+$ or $CD8^+$ cells were detected by MitoSOX staining, *n* = 3. **(F-I)** *Vegfb* WT and cKO mice were infected with Listeria monocytogenes (LM) for 7 days. The mRNA expressions of fatty acid metabolism genes in purified $CD8^+$ T cells from the spleen of infected mice were detected by qPCR, *n* = 3 **(F)**. The cardiolipin levels with NAO staining **(G)**, MMP with TMRM staining **(H)**, and the total mitochondrial mass with MitoTracker Green staining **(I)** in $CD8^+$ T cells from the spleen of infected mice were detected by flow cytometric analysis, *n* = 5. **(J-K)** *Vegfb* WT and cKO $CD8^+$ OT-1 T cells were adoptively transferred to recipient mice, followed by infection with LM-OVA for 7 days. The MFI of NAO and TMRM of the H-$2Kb^+$ $CD8^+$ T cells from the spleen of the infected recipient were measured, *n* = 5. **(L)** The MMP in TCR-stimulated *Vegfb* or *Flt1* WT and cKO $CD4^+$ T cells with or without the addition of VEGF-B (VB) as indicated were analyzed by TMRM staining, *n* = 3. **(M)** The MMP in TCR-stimulated $CD4^+$ T cells treated with VEGF-B neutralization antibody or IgG plus with or without the addition of PG or CL as indicated were analyzed by TMRM staining, *n* = 3. **(N)** Images of mitochondrial ultrastructure analyzed by transmission electron microscopy. The number of cristae per mitochondrion were quantified in the images from TCR-stimulated $CD4^+$ cells treated with VEGF-B neutralization antibody or IgG plus with or without the addition of CL as indicated (+IgG + vehicle group: mito number = 208; +IgG + CL group: mito number = 213; + αVB + vehicle group: mito number = 258; + αVB + CL group: mito number = 214). **(O)** Flow cytometric analysis of the cardiolipin levels with NAO staining in VEGF-B neutralization antibody or IgG treated $CD8^+$ T cells with or without the addition of PG or CL, *n* = 3. **(P)** Flow cytometric analysis of the percentages of Annexin V^+ and PI^+ in TCR-stimulated *Vegfb* WT or cKO $CD4^+$ T cells with or without the addition of PA, PE, PC, PI, PG, or CL, *n* = 3. Data are shown as mean ± SEM. *P* values were calculated using two-tailed unpaired t-test in **(A)**, **(B)**, **(E)** to **(K)**, and two-way ANOVA with Bonferroni's post-hoc test in **(C)**, **(D)**, and **(L)** to **(P)**. ns: not significant, **P* < 0.05, ***P* < 0.01 and ****P* < 0.001.

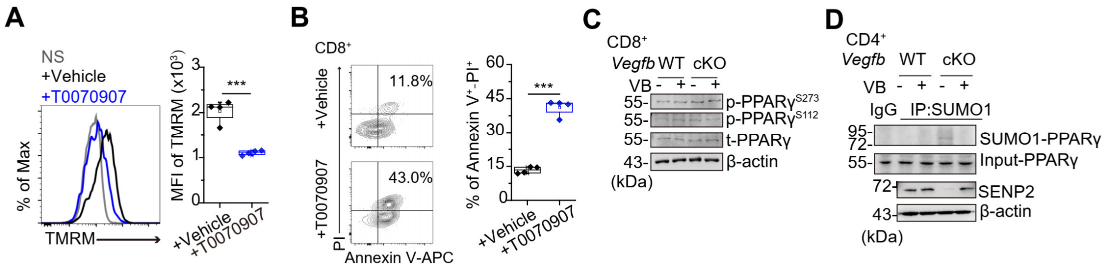

Supplemental Figure 9. PPARγ inhibition destroyed the mitochondria of activated T cells.

(A-B) The TMRM MFI and apoptotic ratios (Annexin V^+ and PI^+) of activated $CD8^+$ T cells with or without T0070907 treatment were detected, *n* = 4. **(C)** The phosphorylation levels of PPARγ (S273 and S112) were blotted in *Vegfb* WT or cKO $CD8^+$ T cells with VEGF-B addition. A representative experiment of three independent replicates is shown. **(D)** SUMOylated-PPARγ was analyzed by IP with anti-SUMO1 antibody and blotting with anti-PPARγ antibody, and SENP2 expression was blotted with anti-SENP2 antibody in TCR-stimulated *Vegfb* WT or cKO $CD4^+$ T cells with the addition of VEGF-

B, A representative experiment of three independent replicates is shown. Box plots are used to show the distributions of numeric data values in **(A)** and **(B)**. *P* values were calculated using two-tailed unpaired t-test in **(A)** and **(B)**, ***$P < 0.001$.

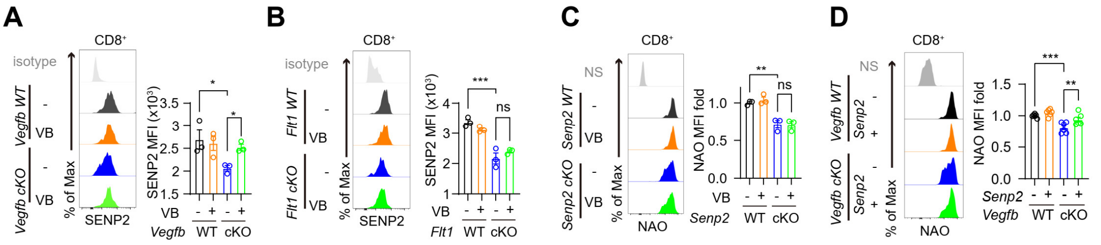

Supplemental Figure 10. VEGF-B regulates *Senp2* expression to support the production of cardiolipin in activated T cells.

(**A**) Flow cytometric analysis of SENP2 MFI in *Vegfb* WT and cKO CD8$^+$ T cells with or without the addition of VEGF-B as indicated, $n = 3$. (**B**) Flow cytometric analysis of SENP2 MFI in *Flt1* WT and cKO CD8$^+$ T cells with or without the addition of VEGF-B as indicated, $n = 3$. (**C**) Flow cytometric analysis of cardiolipin levels by NAO staining in *Senp2* WT and cKO CD8$^+$ T cells with or without the addition of VEGF-B, $n = 3$. (**D**) Flow cytometric analysis of cardiolipin levels by NAO staining in *Vegfb* WT and cKO CD8$^+$ T cells transduced with or without *Senp2* as indicated, $n = 6$. Data are shown as mean ± SEM. *P* values were calculated using two-way ANOVA with Holm-Šídák's post-hoc test in **(A)** to **(D)**. ns: not significant, *$P < 0.05$, **$P < 0.01$ and ***$P < 0.001$.

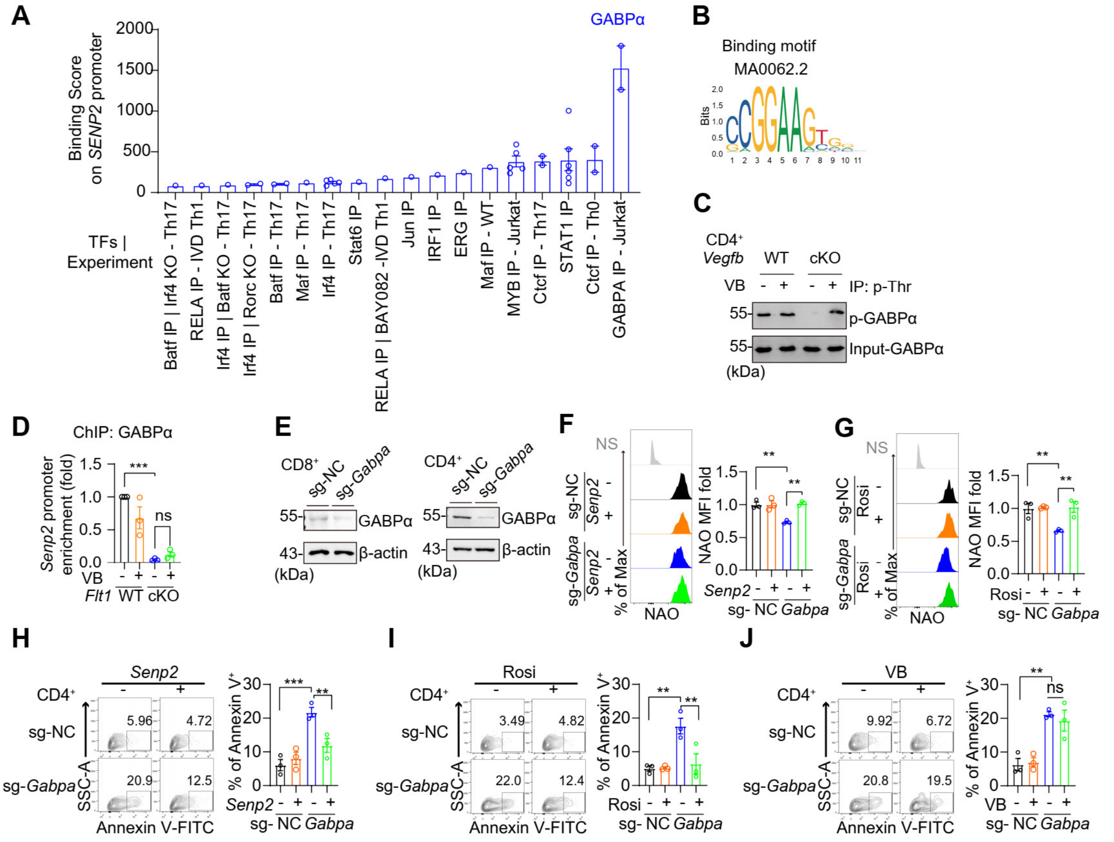

Supplemental Figure 11. VEGF-B maintains the cardiolipin levels in activated T cells through the GABPα-SENP2-PPARγ pathway.

(A) ChIP-seq data from Signaling Pathways Project (SPP) Ominer web tool show that transcription factor (TF) GABPα was binding to the promoter region of the human *SENP2* gene in Jurkat T cells (4, 5). The binding score of TFs on the promoter regions of the human *SENP2* or the mouse *Senp2* gene in T cells from SPP **(A)**. **(B)** The binding motif of GABPα on the promoter region of mouse *Senp2* gene was analyzed by JASPAR (http://jaspar.genereg.net) (6). **(C)** The phosphorylation of GABPα was analyzed by IP with anti-p-Thr antibody and blotting with anti-GABAα antibody in TCR-stimulated *Vegfb* WT or cKO CD4^{+} T cells with or without the addition of VEGF-B (VB) as indicated. A representative experiment of three independent replicates is shown. (**D**) ChIP analysis of the binding of GABPα on the promoter region of *Senp2* in activated *Flt1* WT and cKO T cells with VEGF-B addition, $n = 3$. **(E)** GABPα protein was blotted in CD8^{+} or CD4^{+} T cells with Lenti-CRISPR/cas9-mediated *Gabpa* knockout, sg-NC (negative control), A representative experiment of three independent replicates is shown. **(F-G)** NAO staining was used to detect the levels of cardiolipin in *Gabpa* knockout CD8^{+} T cells that were transduced with or without *Senp2* **(F)**, or treated with or without rosiglitazone (Rosi) **(G)**, $n = 3$. **(H)** The percentages of Annexin V^{+} in *Gabpa* knockout CD4^{+} T cells transduced with or without *Senp2* as indicated, $n = 3$. **(I-J)** The percentages of Annexin V^{+} in *Gabpa* knockout CD4^{+} T cells with or without rosiglitazone treatment **(I)**, or the addition of VEGF-B **(J)** as indicated, $n = 3$. Data are shown as mean ± SEM. *P* values were calculated using two-way ANOVA with Holm-Šídák's post-hoc test in **(D)** and **(F)** to **(J)**. ns: not significant, $^{**}P < 0.01$ and $^{***}P < 0.001$.

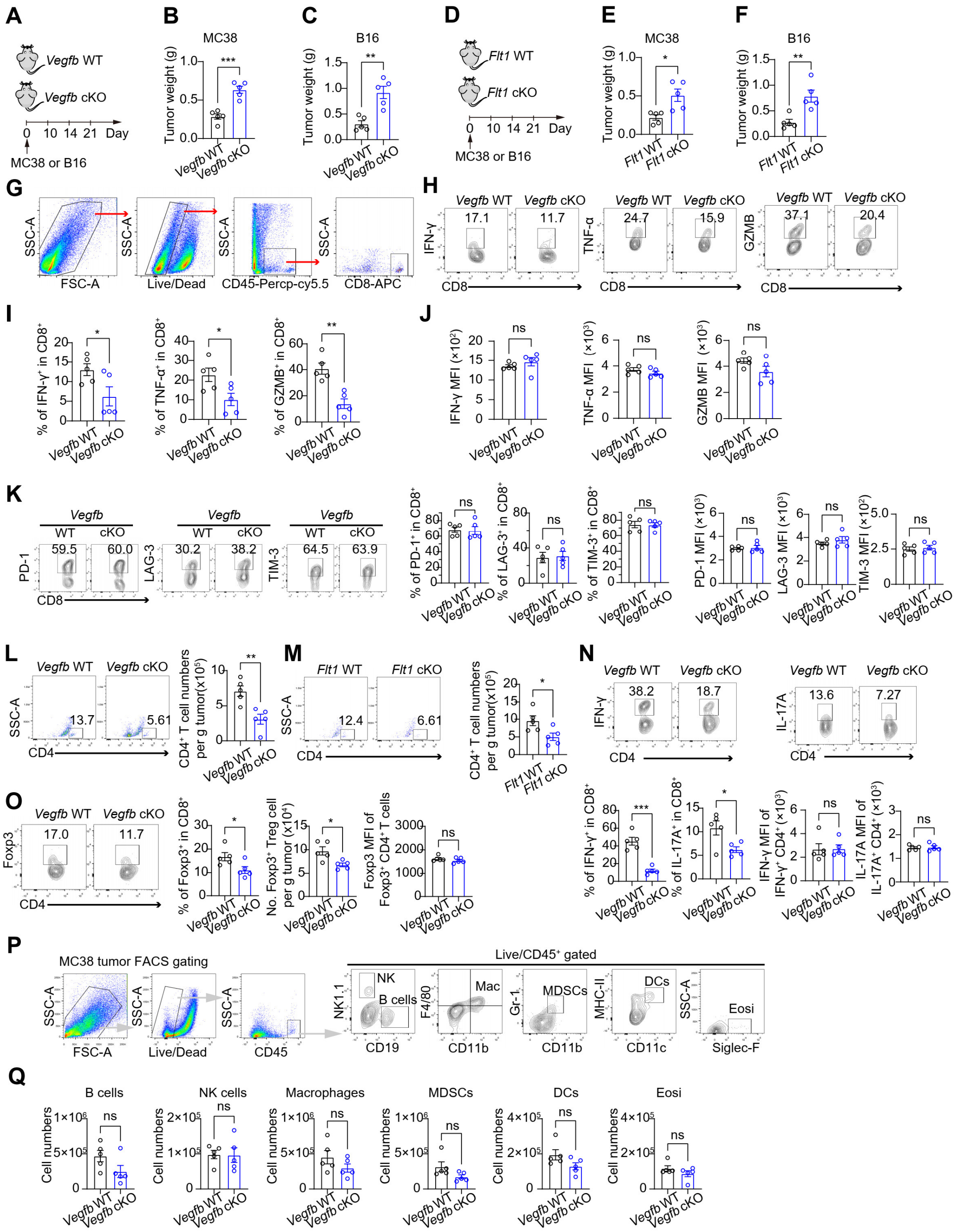

Supplemental Figure 12. Autocrine VEGF-B signaling is crucial for CD8^{+} T cell anti-tumor immunity.

(A-C) The adenocarcinoma MC38 tumor cells or melanoma B16 tumor cells were implanted in *Vegfb* WT or cKO mice by subcutaneous injection. Tumors are weighed 20 days after transplantation of MC38 **(B)** or B16 **(C)**, $n = 5$, respectively. **(D-F)** The adenocarcinoma MC38 tumor cells or melanoma B16 tumor cells were implanted in *Flt1* WT or cKO mice by subcutaneous injection. Tumors are weighed 20 days after transplantation of MC38 **(E)** or B16 **(F)**, $n = 5$, respectively. **(G)** Flow cytometric gating for the analysis of tumor-infiltrating CD8^{+} T cells in the MC38 tumor. **(H-J)** The ratio of IFN-γ^{+}, TNF-α^{+}, or GZMB^{+} in tumor-infiltrating CD8^{+} T cells in MC38 tumors on

Vegfb WT or cKO mice was determined using flow cytometric analysis, $n = 5$ **(H-I)**. The MFI of IFN-γ was measured in IFN-γ positive cells, while the MFI of TNF-α was measured in TNF-α positive cells, and the MFI of GZMB was measured in GZMB positive cells, $n = 5$ **(J)**. **(K)** The ratio of PD-1^+, LAG-3^+, or TIM-3^+ in tumor-infiltrating $CD8^+$ T cells in MC38 tumors on *Vegfb* WT or cKO mice was determined using flow cytometric analysis, and the MFI of PD-1 in PD-1^+ $CD8^+$ T cells, LAG-3 in LAG-3^+ $CD8^+$ T cells, and TIM-3 in TIM-3^+ $CD8^+$ T were measured, $n = 5$ **(K)**. **(L-M)** The tumor-infiltrating number of $CD4^+$ T cells in MC38 tumors in *Vegfb* WT or cKO mice **(L)**, as well as *Flt1* WT or Flt1 cKO mice **(M)**, was detected using FCM, $n = 5$. **(N)** The ratio of IFN-γ^+ and IL-$17A^+$ in tumor-infiltrating $CD4^+$ T cells in MC38 tumors on *Vegfb* WT or cKO mice was determined using flow cytometric analysis, and the MFI of IFN-γ in IFN-γ^+ $CD4^+$ T cells and IL-17A in IL-17A $^+$ $CD4^+$ T were measured, $n = 5$. **(O)** The ratio of $Foxp3^+$ Treg cells in tumor-infiltrating $CD4^+$ T cells in MC38 tumors on *Vegfb* WT or cKO mice was determined, and the MFI of Foxp3 in $Foxp3^+$ $CD4^+$ T cells was measured, $n = 5$. **(P-Q)** Flow cytometric gating for the analysis of tumor-infiltrating $CD19^+$ B cells, F4/80^+ $CD11b^+$ macrophages (Mac), Gr-1^+ $CD11b^+$ myeloid-derived suppressor cells (MDSCs), MHC-II^+ $CD11c^+$ dendritic cells (DCs), and Siglec-F^+ eosinophils (Eosi) in MC38 tumors **(P)**, and the number of these types of immune cells in MC38 tumors was calculated **(Q)**, $n = 5$. Data are shown as mean ± SEM. *P* values were calculated using two-tailed unpaired t-test in **(B)**, **(C)**, **(E)**, **(F)**, and **(I)** to **(Q)**. ns: not significant, $^*P < 0.05$, $^{**}P < 0.01$ and $^{***}P < 0.001$.

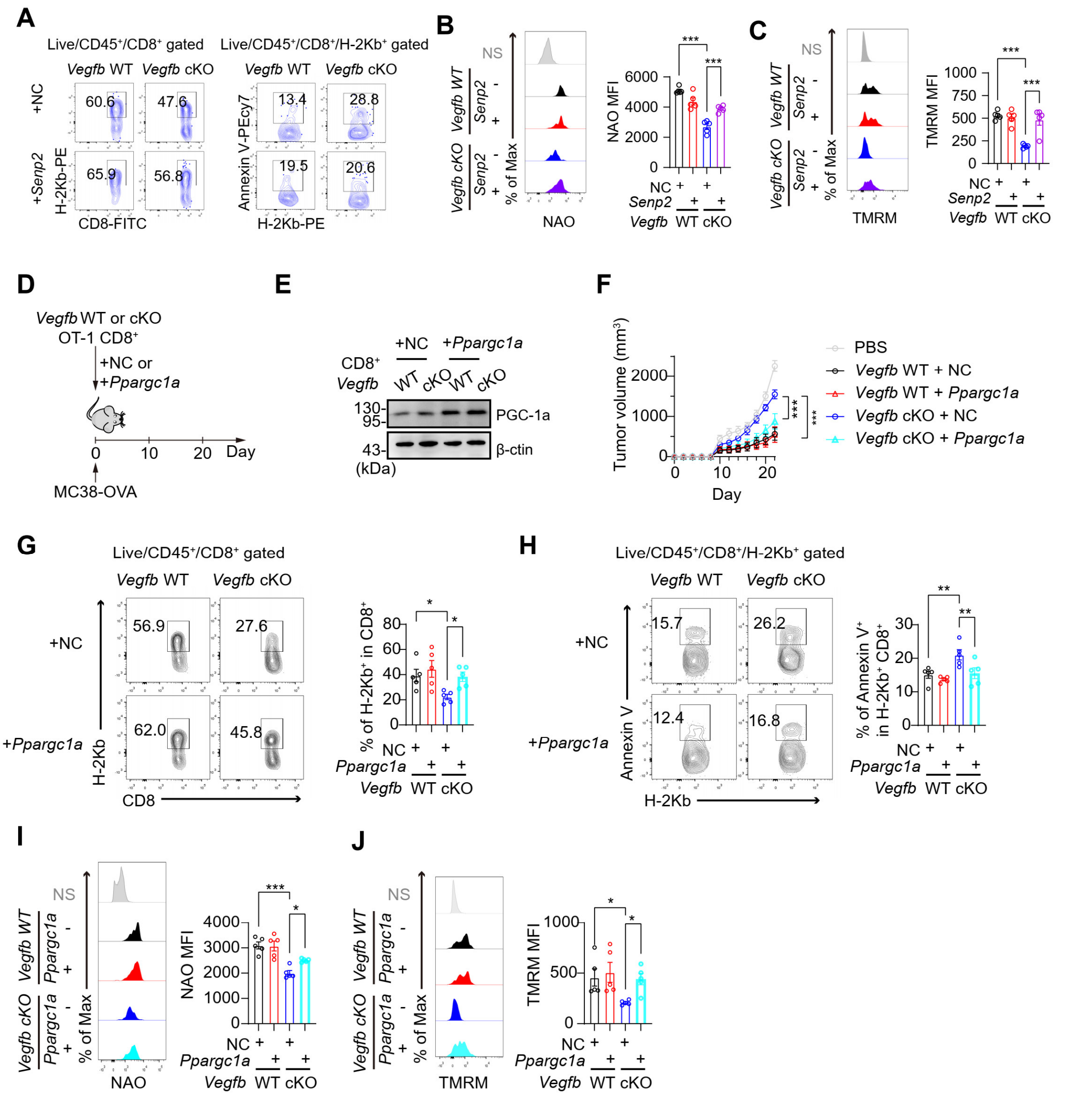

Supplemental Figure 13. VEGF-B signaling is essential for the lipid synthesis and anti-tumor function of CD8$^+$ T cells.

(A-C) In MC38-OVA tumors, the H-2Kb$^+$ ratio in tumor-infiltrating CD8$^+$ T cells, as well as the Annexin V$^+$ ratio **(A)**, NAO MFI **(B)**, and TMRM MFI **(C)** in tumor-infiltrating H-2Kb$^+$ CD8$^+$ T cells, were analyzed using flow cytometry, n = 5. **(D-J)** CD8$^+$ T_N cells from *Vegfb* WT or cKO OT-1 mice were activated by the $OVA_{257\text{-}264}$ peptide followed by lentivirus-mediated *Ppargc1a* overexpression, and then these OT-1 T cells were adaptively transferred (i.v.) to MC38-OVA cells inoculated recipient mice **(D)**. The protein levels of PGC-1α in these OT-1 cells were detected before adaptive transfer **(E)**. The tumor growth curves of MC38-OVA were recorded, n = 5 **(F)**. The H-2Kb$^+$ ratio in tumor-infiltrating CD8$^+$ T cells **(G)**, as well as the Annexin V$^+$ ratio **(H)**, NAO MFI **(I)**, and TMRM MFI **(J)** in tumor-infiltrating H-2Kb$^+$ CD8$^+$ T cells, were analyzed using flow cytometry, n = 5. Data are shown as mean ± SEM. P values were calculated using two-way ANOVA with Benjamini, Krieger, and Yekutieli post-hoc test in in **(B)**, **(C)**, and **(F)** to **(J)**. *$P < 0.05$, **$P < 0.01$ and ***$P < 0.001$.

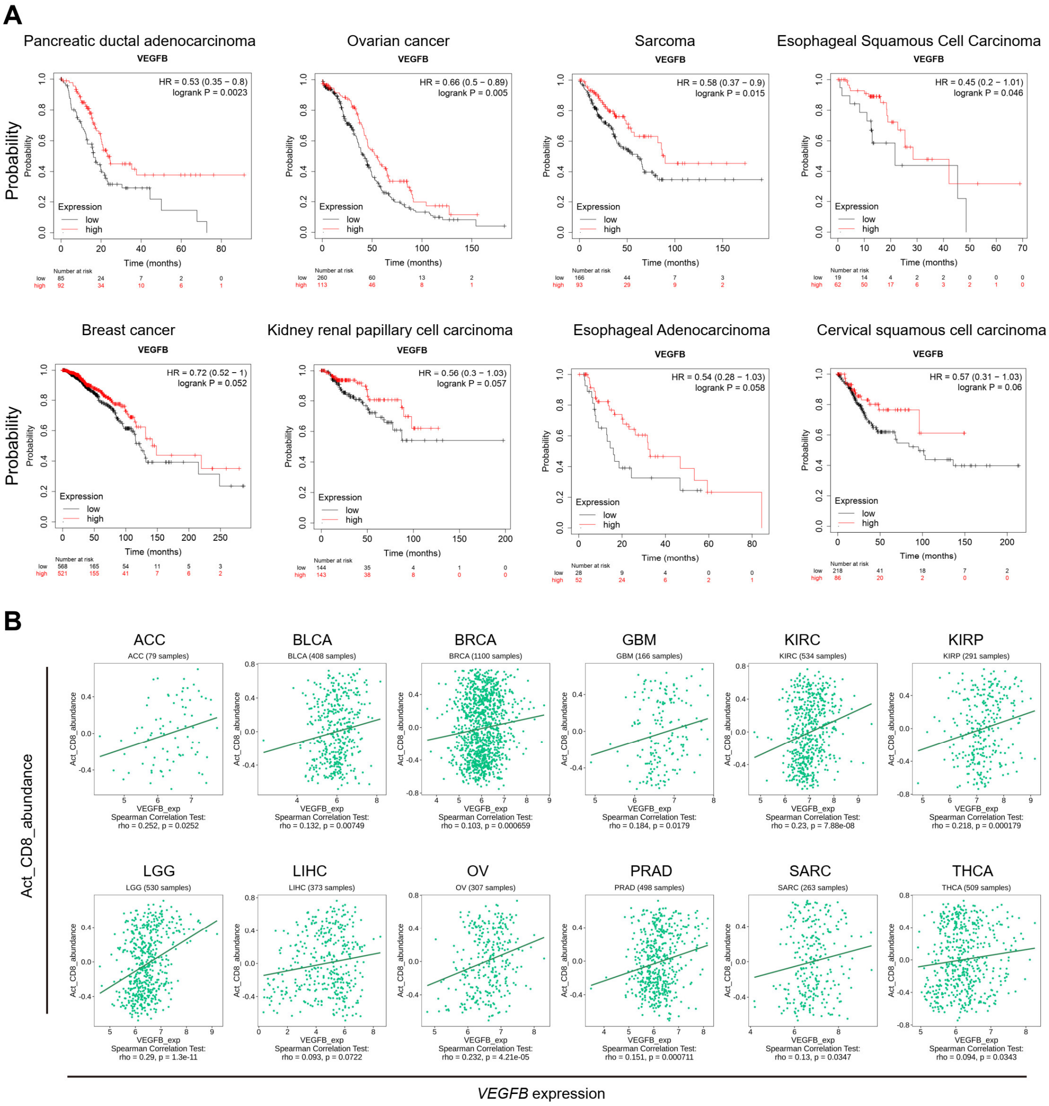

Supplemental Figure 14. The expression of *VEGFB* in human tumor tissues.

(A-B) The survival analysis of *VEGFB* expression in human cancer was conducted using the Kaplan-Meier plotter **(A)**. A Cox proportional hazards regression analysis was performed to examine the correlation between *VEGFB* expression and overall survival. The hazard ratios (HR) and log-rank P values were analyzed **(A)**. **(B)** The correlation of the abundance of tumor-infiltrating activated (Act) CD8^{+} T cells with the expression of *VEGFB* in human tumor tissues was conducted using the TISIDB. Spearman correlations between VEGFB and TILs across human cancers were tested, and the rho value and p value were shown.

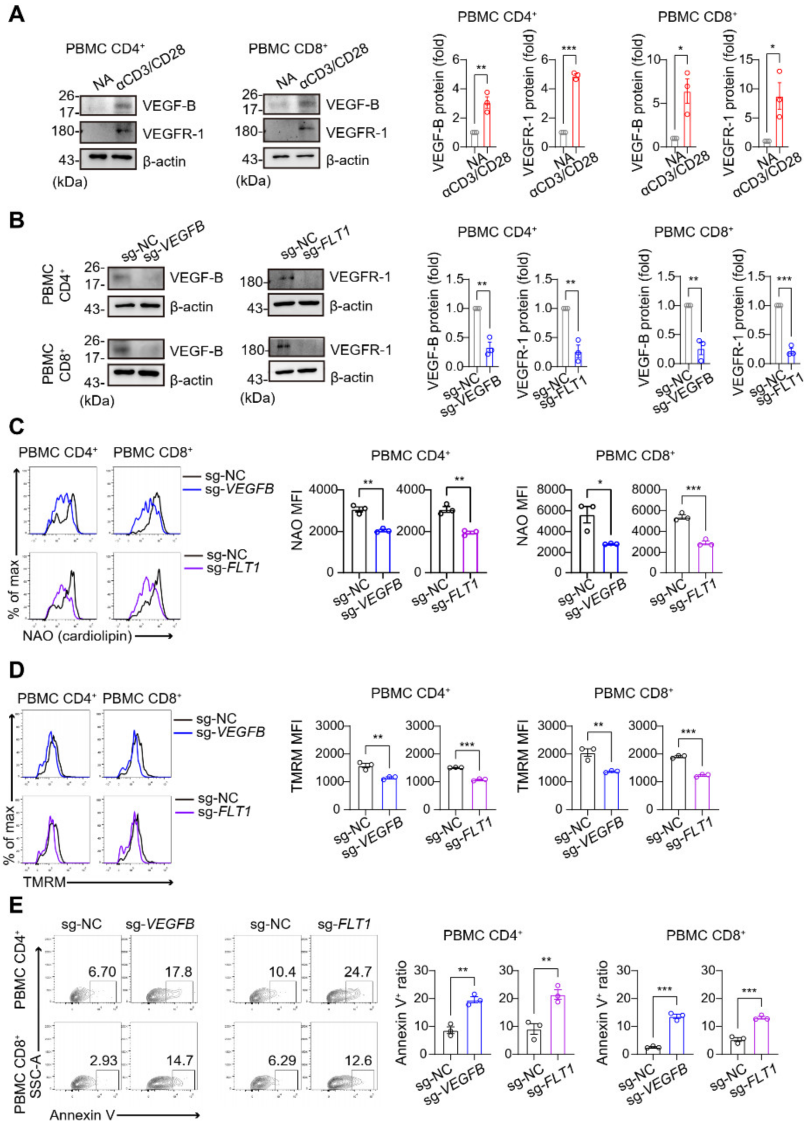

Supplemental Figure 15. The influence of autocrine VEGF-B on human CD4$^+$ and CD8$^+$ T cells.

(A) Healthy human peripheral blood mononuclear cells (PBMC) were used to purify the CD4$^+$ or CD8$^+$ T cells. The protein levels of VEGF-B or VEGFR-1 in human CD4$^+$ or CD8$^+$ T cells with αCD3/CD28 activation were measured, $n = 3$. **(B-E)** The protein levels of VEGF-B or VEGFR-1 in activated human CD4$^+$ or CD8$^+$ T cells with Lenti-CRISPR/cas9-mediated *VEGFB* or *FLT1* knockout were measured **(B)**, sg-NC (negative control), $n = 3$. The cardiolipin levels with NAO staining **(C)**, MMP with TMRM staining **(D)**, and Annexin V$^+$ ratio **(E)** in *VEGFB* or *FLT1* knockout human CD4$^+$ or CD8$^+$ T were detected by flow cytometric analysis, $n = 3$. Data are shown as mean ± SEM. *P* values were calculated using two-tailed unpaired t-test in **(A)** to **(E)**. **P* < 0.05, ***P* < 0.01 and ****P* < 0.001.

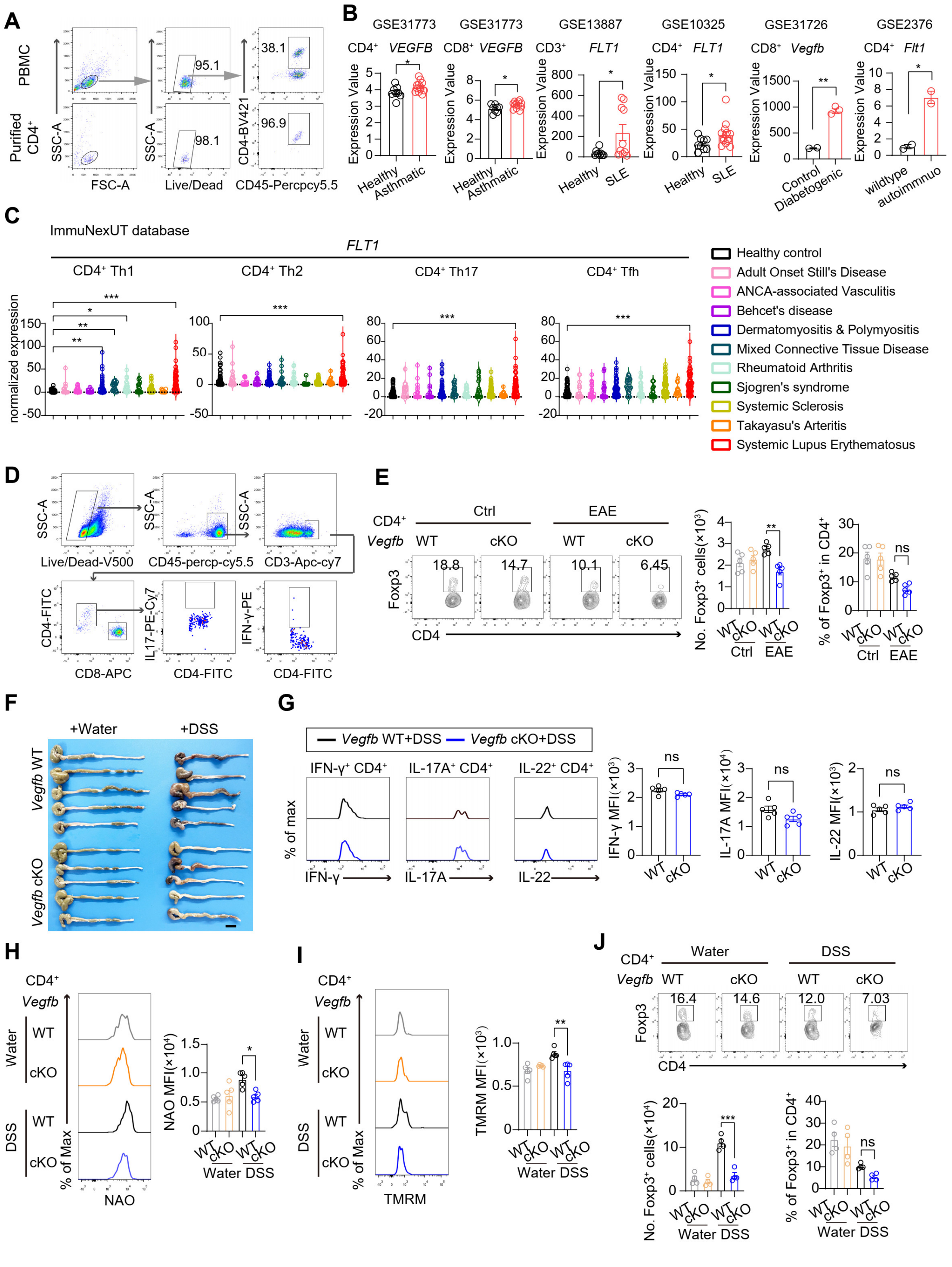

Supplemental Figure 16. Autocrine VEGF-B signaling contributes to T cell-mediated autoimmune disorders.

(A) Flow cytometric gating strategy for human CD4^{+} T cells in PBMC or purified CD4^{+} T cells from human PBMC. **(B)** The expression value of *VEGFB* or *FLT1* in T cells with autoimmune disorders from the GEO database. The expression value of *VEGFB* in the circulating CD4^{+} or CD8^{+} T cells from asthmatic patients (severe and non-severe asthmatics, *n* = 12) or healthy donors (*n* = 8) (GSE31773). The expression value of *FLT1* in CD3^{+} T cells from SLE patients (*n* = 10) or healthy donors (*n* = 9) (GSE13887). The expression value of *Flt1* in CD4^{+} T cells from SLE patients (*n* = 14) or healthy

donors (n = 9) (GSE10325). The expression value of *Vegfb* in diabetogenic G9C8 clone (n = 3) or transgenic control $CD8^+$ T cells (n = 2) (GSE31726). The expression value of *Flt1* in $CD4^+$ T cells from an autoimmune mouse model with a Sanroque mutant induced by ENU (n = 2) or wildtype mouse (n = 2) (GSE2376). **(C)** The expression value of *FLT1* in $CD4^+$ T helper cells from immune-mediated disease patients or healthy donors (ImmuNexUT database) (7). **(D)** Flow cytometric gating strategy for the ratios of IFN-γ^+ or IL-$17A^+$ in $CD4^+$, and IFN-γ^+ in $CD8^+$ in the brain tissues of EAE mice. **(E)** $Foxp3^+$ Treg cell abundance in the brains of EAE mice was identified through flow cytometry. The ratio of $Foxp3^+$ within brain $CD4^+$ T cells, as well as the cell numbers of (No.) $Foxp3^+$ Treg cells in the brains of EAE mice, were quantified. n = 5. **(F)** DSS colitis was generated in *Vegfb* WT or cKO mice (n = 5); the scale bar represents 1 cm. **(G)** Flow cytometry was utilized to analyze the MFI of IFN-γ in IFN-γ^+, IL-17A in IL-$17A^+$, and IL-22 in IL-22^+ colon $CD4^+$ T cells from DSS-colitis mice, n = 5. **(H-I)** The MFI of NAO **(H)** and TMRM **(I)** in the colon $CD4^+$ T cells from DSS-colitis mice were measured, n = 5. **(J)** The ratio of $Foxp3^+$ within colon $CD4^+$ T cells, as well as the cell numbers of $Foxp3^+$ Treg cells in the colon of DSS mice, were quantified. n = 4. Data are shown as mean ± SEM. *P* values were calculated using two-tailed unpaired t-test in **(B)** and **(G)**, and two-way ANOVA with Bonferroni's post-hoc test in **(C)**, **(E)**, and **(H)** to **(J)**. ns: not significant, $^*P < 0.05$, $^{**}P < 0.01$ and $^{***}P < 0.001$.

Supplemental Methods

Cell culture.

HEK-293T (ATCC CRL-11268), MC38, ovalbumin (OVA) expressing MC38 (MC38-OVA), and B16-F10 cells cultured in Dulbecco's modified Eagle's medium (DMEM, 4500 mg/l glucose, 4 mM L-glutamine) containing 10% fetal bovine serum (FBS), 100 units/ml penicillin, and 100 μg/ml streptomycin. MC38, MC38-OVA, and B16-F10 cells were sourced from the lab of Ralph R. Weichselbaum. All of the cell lines are mycoplasma free.

T cell culture.

$CD4^+$ and $CD8^+$ naïve T cells were isolated from the spleen of mice. $CD4^+$ and $CD8^+$ naïve T cells ($CD4^+$ or $CD8^+$ and $CD44^-$ $CD62L^+$) were purified by EasySep Mouse Naive $CD4^+$ T or $CD8^+$ Cell Isolation Kits (STEMCELL) or sorted using a MoFlo Astrios (BeckMan). For T cell activation ex vivo, naïve T cells were stimulated with anti-CD3 (5 μg/mL) + anti-CD28 (2 μg/mL) + IL-2 (100 U/mL) for 3 days in T cell media (RPMI 1640 + 10% FBS + 100 U/mL penicillin/streptomycin + 2 mM L-glutamine + 55 μM β-mercaptoethanol). For antigen specific T cell activation ex vivo, $CD8^+$ OT-1 naïve T cells were activated with $OVA_{257\text{-}264}$ peptide (1 μg/mL, SIINFEKL, Sigma-Aldrich) and IL-2 (100 U/mL, PeproTech) for 3 days. For memory T cell development ex vivo, naive T cells were activated with $OVA_{257–264}$ peptide (1 μg/mL) or anti-CD3 (5 μg/ml)/anti-CD28 (2 μg/ml) with IL-2 (100 U/ml, PeproTech) for 3 days and then cultured in the presence of IL-15 (10 ng/ml, PeproTech) for another 3 days in T cell media.

$CD4^+$ helper T cell differentiation ex vivo.

To induce helper T cell differentiation ex vivo, $CD4^+$ naïve T cells were activated with anti-CD3/CD28 and IL-2 for 3 days. Subsequently, they were cultured with specific polarizing cytokines and treated with anti-VEGF-B (1 μg/mL) or normal goat IgG control (1 μg/mL) for an additional 3 days. The specific combinations of cytokines and antibodies used were as follows: IL-12 (20 ng/mL) + anti-IL-4 (10 ng/mL) for Th1 cells, IL-4 (100 ng/mL) + anti-IFN-γ (10 ng/mL) + anti-IL-12 (10 ng/mL) for Th2 cells, TGF-β1 (5 ng/mL) + anti-IL-4 (10 ng/mL) + anti-IFN-γ (10 ng/mL) for Treg cells, and TGF-β1 (1 ng/mL) + IL-6 (100 ng/mL) + anti-IL-4 (10 ng/mL) + anti-IFN-γ (10 ng/mL) for

Th17 cells. The catalog numbers and sources of cytokines and antibodies are shown in **Supplementary Table 3.**

Chemical compounds, lipids, and neutralization antibody treatment.

T cells were activated by αCD3/CD28 + IL-2 for 48 hours, followed by treatment with following reagents for 12 hours: recombinant mouse VEGF-B (1 ng/mL, #2595-VE-025, R&D), PGC-1α activator ZLN005 (20 μM, #SML0802, Sigma), CCCP (20 μM, #M20036, Invitrogen), N-acetyl-L-cysteine (NAC, 5 μM, #A7250, Sigma-Aldrich), glutathione (GSH, 5 μM, #G4251, Sigma-Aldrich), Rotenone (0.4 μM, #S2348, selleck), Antimycin A (0.04 μM, #sc-202467A, Santa cruz), mitoTEMPO (#T19428, TargetMol), Etomoxir (5 μM, #E1905, Sigma-Aldrich), C75 (40 μM, #T10657, TargetMol), H_2O_2 (0~100 μM). Rosiglitazone (5 μM, #S2556, Selleck), T0070907 (5 μM, #S2871, Selleck), PA (50 μM, CAS# 383907-53-7, #840101P), PE (50 μM, CAS#477241-93-3, #840025P), PC (50 μM, CAS# 383907-52-6, #840053P), PI (50 μM, CAS# 383907-33-3, #840042P), PG (50 μM, CAS#383907-64-0, #841138P), and Cardiolipin (50 μM, CAS#383907-10-6, #840012P). These phospholipids were purchased from Avanti Polar Lipids (Sigma-Aldrich). Anti-VEGF-B (1 μg/mL; #AF-590, R&D Systems) and normal goat IgG control (1 μg/mL; #AB-108-C, R&D Systems) were used for VEGF-B blocking. Anti-VEGFR-1 (1 μg/mL; #AF471, R&D Systems) and normal Goat IgG control (1 μg/mL; #AB-108-C, R&D Systems) were used for VEGFR-1 blocking.

Plasmid and lentiviral transduction.

The *Vegfb* shRNA targeting sequences (sh-*Vegfb*-#1: CCAATGTGAATGCAGACCA; sh-*Vegfb*-#2: GCAATGTGGTCAAACAACT; sh-*Vegfb*-#3: ACAGCCAATGTGAA-TGCAG) were constructed in pGIPZ vectors from the cDNA library platform of Shanghai Jiao Tong University School of Medicine. Mouse *Senp2* cDNA was cloned into pCDH-CMV-MCS-EF1 lentiviral vectors, and Mouse *Ppargc1a* cDNA was cloned into pLVX-IRES-Puro lentiviral vectors. The sgRNA sequence targeting the mouse *Gabpa* gene (AACAAAACAAAAAACCAAAG) was constructed in the Lenti-CRISPRv2-mCherry vector. The lentiviral plasmids were packaged using the standard lentiviral package strategy. The transfection reagent Lipofectamine 3000 (Cat# L3000-008, Invitrogen, CA, USA) was used for lentiviral packaging. Lentiviral particles were used for transduction in naïve or activated $CD8^+$ T cells. For lentiviral transduction in activated T cells, $CD8^+$ OT-1 T cells were activated ex vivo for 48 hours with

αCD3/CD28 or $OVA_{257-264}$ peptide (1 μg/mL), combined with IL-2 (100 U/mL). The naïve or activated T cells were transduced with lentiviral particles plus polybrene (5 μg/mL) and spun at 1,200 r.p.m. for 60 minutes at 30 °C in a Beckman centrifuge. The supernatant was removed after infection and replaced with T cell culture medium containing IL-2 (100 U/mL). The T cells were collected for further experiments 24 hours after transduction.

Human T cell isolation, stimulation, and lentiviral transduction.

Human $CD4^+$ or $CD8^+$ T cells from peripheral blood mononuclear cells (PBMC) of healthy individuals were purified using EasySep Human $CD4^+$ T Cell Isolation Kits (#17952, Stemcell) or EasySep Human $CD8^+$ T Cell Isolation Kits (#17953, Stemcell), respectively. Purified PBMC $CD4^+$ or $CD8^+$ T cells were stimulated with human anti-CD3/anti-CD28 T Cell Activation Beads (cat#422603, Biolegend) with IL-2 (cat#589102, Biolegend) for 3 days. To achieve CRISPR-Cas9-mediated knockout using lentivirus, the activation of human $CD4^+$ or $CD8^+$ T cells was carried out by anti-CD3/anti-CD28 stimulation for 24 hours. Subsequently, the cells were infected with the packaged lentivirus in the presence of 5 mg/mL polybrene. After 48 hours, the transduced T cells were collected for further analysis. The sgRNA sequences targeting the human *VEGFB* gene (GGTACCCGAGCAGTCAGCTG) or *FLT1* gene (GAGTGGTAGCAGTACAATTG) were constructed in Lenti-CRISPRv2 vectors.

Flow cytometry (FCM).

Fluorochrome-labeled antibodies (Biolegend, eBioscience, and BD Pharmingen) were listed in **Table S3**. MitoTracker Red (#M7512, Invitrogen), MitoTracker Green (#M7514, Invitrogen), MitoTracker Deep Red (#M22426, Invitrogen), MitoProbe™ TMRM Kit (#M20036, Invitrogen), MitoSOX (#MX4301, Invitrogen), and Annexin V/PI (#BMS500FI-100, and #88-8007-72, eBioscience) staining were performed according to the manufacturer's instructions. OVA-specific $CD8^+$ T cells were stained with H2-Kb $OVA_{257-264}$ MHC-peptide tetramers (#TS-5001-1C/TS-5001-2C, MBL, Japan). Before membrane staining, cells were pre-incubated with purified anti-CD16/CD32 (#101302, BioLegend) for 30 minutes at 4°C. For the intracellular staining of cytokines and VEGF-B in activated T cells, the cells were re-stimulated with a T cell activation cocktail for the last 2.5 hours. This cocktail includes PMA, ionomycin, and Brefeldin A (cat#423303, Biolegend). The Cytofix/Cytoperm

Fixation/Permeabilization Kit (#554714, BD Biosciences) was used for intracellular cytokine staining. For intracellular staining of VEGF-B in resting (T_N, TC_M, and T_{EM}) or re-stimulated T cells, the fixed and permeabilized cells were stained with antibodies against VEGF-B (AF-590, 1:200, R&D Systems) for 90 minutes at room temperature, followed by staining with a fluorescein-labeled secondary antibody for 60 minutes at room temperature. Dead cells were excluded using the Fixable Viability Dye eFluor 450 (#65-0863-14, eBioscience) or LIVE/DEAD™ Fixable Violet Dead Cell Stain Kit (#L34955/L34976, Thermo Fisher). Cells were collected on the BD FACSverseTM or LSR Fortessa X-20 cell analyzer (BD Biosciences) and analyzed using FlowJo (TreeStar) software. Other materials were listed in **Table S3**.

Confocal fluorescence microscopy.

T cells were stained with fluorescein-labeled antibodies against CD4 or CD8. For VEGF-B staining, T cells were fixed, permeabilized, and blocked with 5% Donkey Serum. Cells were further stained with anti-VEGF-B (AF-590, 1:200, R&D Systems) for 90 minutes at room temperature, followed by staining with a fluorescein-labeled secondary antibody for 60 minutes at room temperature, and followed by nuclear counterstaining with DAPI. Images were acquired using a Leica TCS Sp8 STED confocal microscope at the Core Facility of Basic Medical Sciences, Shanghai Jiao Tong University School of Medicine (Shanghai, China).

Alkaline phosphatase labeled VEGF-B binding assay.

The alkaline phosphatase (AP) labeled protein binding assay is based on the description in previous reports (Jaworski A et. al., Science, 2015, PMID: 26586761). The recombinant mouse VEGF-B protein (#2595-VE-025, R&D) was used to generate alkaline phosphatase labeled VEGF-B (VEGF-B-AP) by Alkaline phosphatase Lightning-Link kits (ab102850). For VEGF-B-AP binding to T cells, purified $CD4^+$ or $CD8^+$ T cells were fixed with fixation buffer (#00-8222-49, eBioscience) for 15 min. After fixation, the endogenous AP activity of T cells was quenched by incubation at 65 °C for 3 hours. Then, the T cells were rinsed twice in AP binding buffer (HBSS with 20 mM HEPES-pH7.0, 0.2% BSA, 5 mM $CaCl_2$, 1 mM $MgCl_2$, and 2 μg/mL Heparin) and incubated with diluted VEGF-B-AP protein (1 ng/mL) in binding buffer for 90 min at 4°C. After incubation, the T cells were rinsed three times with binding buffer. AP

activity was visualized by incubating cells with the NBT/BCIP staining kit (#C3206; Beyotime). Images were acquired using an OLYMPUS microscope (cellSens Entry v1.18).

Cell viability assay.

To analyze the cell viability in TCR-stimulated $CD4^+$ or $CD8^+$ T cells. The CellTiter-Glo Luminescent Cell Viability Assay (#G7572, Promega) was performed according to the manufacturer's instructions.

Mitochondrial Respiration and Glycolytic Rate Measurement.

The Seahorse XFe96 extracellular flux analyzer (Agilent Technologies) was utilized to measure the oxygen consumption rates (OCR) and extracellular acidification rates (ECAR) of T cells. The XF Cell Mito stress test kits (cat#103015-100, Agilent) and the XF Glycolytic stress test kits (cat#103020-100, Agilent) were purchased from Agilent Technologies, Inc. Stimulated T cells were seeded at a density of 2×10^5 per well by Cell-Tak (cat#354240, Corning). OCR measurement was conducted using 1 μM oligomycin, 1.5 μM FCCP, and 100 nM rotenone/1 μM antimycin A. ECAR measurement utilized 10 mM glucose, 1 μM oligomycin, and 50 mM 2-DG. Data collection was conducted using Seahorse Wave (2.6.1.53) software.

Mitochondrial Ca^{2+} uptake assays.

The fluorescence-based assay was used to test the mitochondrial Ca^{2+} uptake rate in T cell as described previously (8, 9). Briefly, 2×10^5 T cells were resuspended in recording buffer (120 mM KCl, 25 mM HEPES, 2 mM KH_2PO_4, 5 mM succinate, 1 mM $MgCl_2$, 5 μM thapsigargin, pH 7.2) with 30 μM digitonin (cat# D141, Sigma Aldrich) and 0.25 μM Calcium green (cat #20500, AAT Bioquest). Fluorescence was monitored with Spark (Tecan) using filter sets (Excitation 485 nm/Emission 535 nm) at 37°C before and after 10 μM Ca^{2+} injection. The relative rate of Ca^{2+} uptake is calculated as the linear fit from the fluorescence.

Mitochondria isolation.

The procedure used for mitochondria isolation was described in our previous study (Wang et al., 2019, Molecular Cell, 75, 823-834) (10, 11). Briefly, cells were incubated in ice-cold isolation buffer TD [135 mM NaCl, 5 mM KCl, 25 mM Tris-HCl (pH 7.5)]

containing complete EDTA-free protease inhibitor mixture (Roche). After centrifuging at 600*g* for 10 min, the pellet was washed with 10 mL ice-cold TD buffer and centrifuged again at 600*g* for 10 min. The pellet was then re-suspended in ice-cold isolation buffer MS containing 210 mM mannitol, 70 mM sucrose, 5 mM Tris-HCl (pH 7.5), 1 mM EGTA, 0.5 mg/mL BSA and a protease inhibitor mixture. The lysate was further homogenized in this buffer with a Glass-Teflon motorized homogenizer for 50 times. The mitochondrial fraction was isolated by differential centrifugation at 1,000*g* for 5 min and 3,500*g* for 15 min. Subsequently, the purified mitochondria were dissolved in MS buffer for -80 °C stock.

Lipidomic analysis.

A freeze-thaw cycle approach was used for lipid extraction from T cells in sample preparation. The lipidomic profiling was performed using the Ultra-Performance Liquid Chromatography Mass Spectrometry (UPLCMS/MS) system (UPLC, Agilent 1290; MS, Applied Biosystems SCIEX 6500+QTRAP) at the Core Facility of Basic Medical Sciences, Shanghai Jiao Tong University School of Medicine. The samples were separated with reverse-phase chromatography (ACQUITY BEH C18 (1.7 µm, 2.1x100 mm), Waters). The experiment was carried out using Multi Reaction Monitoring (MRM) mode, in which each type of lipid was corrected with corresponding lipid isotope standards to ensure the accuracy of the experiment. Mass spectrometric data were processed with Analyst 1.6.3 software (AB Sciex). The data were processed with Sciex OS software, and the analytical data consisted of RT and normalization by ISTDs, baselining towards blank runs to remove background noise.

Free fatty acid and acylcarnitine quantification.

Total free fatty acid in T cells was measured by the Free Fatty Acid Quantification Colorimetric/Fluorometric Kit (Catalog #K612-100, BioVision). The procedure for acylcarnitine analysis was described in a previous study(10, 11). In brief, total T cell lysates were precipitated with acetonitrile containing internal standards; the supernatant was separated by centrifugation and then analyzed by tandem mass spectrometry (TSQ Vantage, Thermo Fisher Scientific) at the Core Facility of Basic Medical Sciences, Shanghai Jiao Tong University School of Medicine. L-carnitine, acylcarnitine, and their internal standards (Sigma-Aldrich) were analyzed in positive-ion multiple reaction monitoring (MRM) mode. The data were processed using LCquan 2.7 software

(Thermofisher Scientific) to generate the chromatographic peak areas of each analyst and their response ratios to internal standards. Then the concentration of acylcarnitines was calculated from the response ratios and the concentration of their internal standards using the isotope dilution method.

Western blot and immunoprecipitation.

Cells were lysed in radioimmunoprecipitation assay buffer (RIPA) and processed for immunoprecipitation and western blot as described previously. The following antibodies were used for western blot: anti-VEGF-B (1:1000, #AF-590, R&D Systems), anti-VEGF-R1 (1:1000, #ab32152, Abcam), anti-β-actin (1:5000, #A5441, Sigma-Aldrich), anti-phospho-PI3K p85 (1:1000, #4228, CST), anti-PI3K p85 (1:1000, #4257, CST), anti-phospho-ERK1/2 (1:1000, #4370s, CST), anti-ERK1/2 (1:1000, #4695s, CST), anti-phospho-PPAR-γ (pSer112) (1:1000, #SAB4503977, Sigma-Aldrich), anti-phospho-PPAR-γ (pSer273) (1:1000, #bs-4888R, Bioss Inc), anti-PPAR-γ (1:1000, #SAB4502262, Sigma-Aldrich), anti-GABPα (H-2) (1:1000, #sc-28311, Santa Cruz). The appropriate conjugated secondary antibodies were stained at room temperature for 1 hour. To detect the phosphorylation of VEGFR-1, a Phospho-Tyrosine (P-Tyr-1000) antibody (1:100, #8954, CST) was used for immunoprecipitation. To detect the phosphorylation of GABPα, a p-Thr (H-2) antibody (#sc-5267, Santa Cruz) was used for immunoprecipitation. To detect the SUMOylation of PPARγ, an anti-SUMO1 antibody was used for immunoprecipitation (Cai et al., 2017) (12). Other antibodies and materials are listed in **Table S3**.

ChIP analysis.

Chromatin immunoprecipitation (ChIP) of T cells was analyzed by the SimpleChIP® Plus Sonication Chromatin IP Kit (CST, #56383) according to the manufacturer's instructions. GABPα antibody (Santa Cruz Biotechnology, #sc-28311) was used for chromatin pulldown. Forward primer: 5′-ACGTATGCGTGCGGAATGAG-3′ and reverse primer: 5′-TTTACCCGGATTGCCGTCAT-3′ were used to quantify *Senp2* promoter regions by qRT-PCR.

In silico analysis.

The *VEGFB* or *FLT1* expression levels in T cells were obtained from the GEO database,

including the following datasets: The expression of *Vegfb* and marker genes for naïve and effector $CD8^+$ T cells with αCD3/CD28 stimulation for 3 days (GSE54215) (1). The expression value of *VEGFB* in the circulating $CD4^+$ or $CD8^+$ T cells from asthmatic patients (severe and non-severe asthmatics) or healthy donors (GSE31773). The expression value of *FLT1* in $CD3^+$ T cells from SLE patients or healthy donors (GSE13887). The expression value of *Flt1* in $CD4^+$ T cells from SLE patients or healthy donors (GSE10325). The expression value of *Vegfb* in diabetogenic G9C8 clone or transgenic control $CD8^+$ T cells (GSE31726). The expression value of *Flt1* in $CD4^+$ T cells from an autoimmune mouse model with a Sanroque mutant induced by ENU or a wild-type mouse (GSE2376). The expression value of *FLT1* in $CD4^+$ T helper cells in immune-mediated disease patients or healthy donors is from the ImmuNexUT database (7).

Utilizing data from TCGA (The Cancer Genome Atlas), the survival analysis of *VEGFB* expression in human cancer was conducted using the Kaplan-Meier plotter for Pan-cancer (www.kmplot.com) (13). A Cox proportional hazards regression analysis was performed to examine the correlation between *VEGFB* expression and overall survival. The hazard ratios (HR) and log-rank P values were analyzed. The correlation of the abundance of tumor-infiltrating activated (Act) $CD8^+$ T cells with the expression levels of *VEGFB* in human tumor tissues was conducted using the TISIDB (a web portal for tumor and immune system interaction, http://cis.hku.hk/TISIDB/) (14). Spearman correlations between *VEGFB* and TILs across human cancers were tested, and the rho value and p value were shown.

Supplemental Tables

Table S1. Clinical features of SLE patients and healthy volunteers

**Serial number**	**Disease**	**Age**	**Gender**
SLE #1	SLE	31	female
SLE #2	SLE	30	female
SLE #3	SLE	26	female
SLE #4	SLE	28	female
SLE #5	SLE	29	female
SLE #6	SLE	32	female
SLE #7	SLE	33	female
SLE #8	SLE	47	female
SLE #9	SLE	27	female
SLE #10	SLE	17	female
Healthy #1	Healthy	27	male
Healthy #2	Healthy	25	male
Healthy #3	Healthy	34	male
Healthy #4	Healthy	29	female
Healthy #5	Healthy	45	female

Table S2. Primers for qRT-PCR.

**Gene-Primer names**	**Primers (5'-3')**
**1. Primers for metabolic regulation factors**	
**1.1 Myokines**	
Osm-F	CCCGGCACAATATCCTCGG
Osm-R	TCTGGTGTTGTAGTGGACCGT
Mstn-F	AGTGGATCTAAATGAGGGCAGT
Mstn-R	GTTTCCAGGCGCAGCTTAC
Fndc5-F	TTGCCATCTCTCAGCAGAAGA
Fndc5-R	GGCCTGCACATGGACGATA
Gdf15-F	CTGGCAATGCCTGAACAACG

Gdf15-R	GGTCGGGACTTGGTTCTGAG
Sparc-F	GTGGAAATGGGAGAATTTGAGGA
Sparc-R	CTCACACACCTTGCCATGTTT
Il6-F	CTGCAAGAGACTTCCATCCAG
Il6-R	AGTGGTATAGACAGGTCTGTTGG
Il18-F	GACTCTTGCGTCAACTTCAAGG
Il18-R	CAGGCTGTCTTTTGTCAACGA
Il15-F	ACATCCATCTCGTGCTACTTGT
Il15-R	GCCTCTGTTTTAGGGAGACCT
**1.2 Hepatokine**	
Angptl6-F	CTGGGCCGTCGTGTAGTAG
Angptl6-R	CAGTCCTCTAGGAGTATCAGCAG
Ahsg-F	ATCCGCTCCACAAGGTACAG
Ahsg-R	GGTCCAAAGCATGGCAAGT
Shbg-F	TCTGCTGTTGCTACTACTGATGC
Shbg-R	GGGCCATTGCTGAGGTACTTA
Selenop-F	ATCAACCAGCTCCTGTGTAAG
Selenop-R	GCAGACCCTGACTTCTCAAATA
Igf1-F	CTGGACCAGAGACCCTTTGC
Igf1-R	GGACGGGGACTTCTGAGTCTT
Igf2-F	GTGCTGCATCGCTGCTTAC
Igf2-R	ACGTCCCTCTCGGACTTGG
Fgf21-F	CTGCTGGGGGTCTACCAAG
Fgf21-R	CTGCGCCTACCACTGTTCC
**1.3 Adipokines**	
Nampt-F	GCAGAAGCCGAGTTCAACATC
Nampt-R	TTTTCACGGCATTCAAAGTAGGA
Adipoq-F	TGTTCCTCTTAATCCTGCCCA
Adipoq-R	CCAACCTGCACAAGTTCCCTT
Lep-F	GAGACCCCTGTGTCGGTTC
Lep-R	CTGCGTGTGTGAAATGTCATTG
Retn-F	AAGAACCTTTCATTTCCCCTCCT
Retn-R	GTCCAGCAATTTAAGCCAATGTT
Sfrp5-F	CACTGCCACAAGTTCCCCC
Sfrp5-R	TCTGTTCCATGAGGCCATCAG
Rbp4-F	AGTCAAGGAGAACTTCGACAAGG
Rbp4-R	CTTGGCTGTGGCGCTCATA
**1.4 Other secreted factors**	
Egf-F	AGCATCTCTCGGATTGACCCA
Egf-R	CCTGTCCCGTTAAGGAAAACTCT
Ngf-F	CCAGTGAAATTAGGCTCCCTG
Ngf-R	CCTTGGCAAAACCTTTATTGGG
Bdnf-F	TCATACTTCGGTTGCATGAAGG

Bdnf-R	AGACCTCTCGAACCTGCCC
Vegfa-F	GCACATAGAGAGAATGAGCTTCC
Vegfa-R	CTCCGCTCTGAACAAGGCT
Vegfb-F	GCCAGACAGGGTTGCCATAC
Vegfb-R	GGAGTGGGATGGATGATGTCAG
Vegfc-F	GAGGTCAAGGCTTTTGAAGGC
Vegfc-R	CTGTCCTGGTATTGAGGGTGG
Vegfd-F	TTGAGCGATCATCCCGGTC
Vegfd-R	GCGTGAGTCCATACTGGCAAG
Pgf-F	TCTGCTGGGAACAACTCAACA
Pgf-R	GTGAGACACCTCATCAGGGTAT
Fgf15-F	ATGGCGAGAAAGTGGAACGG
Fgf15-R	CTGACACAGACTGGGATTGCT
Fgf23-F	ATGCTAGGGACCTGCCTTAGA
Fgf23-R	AGCCAAGCAATGGGGAAGTG
Nrg1-F	ATGGAGATTTATCCCCCAGACA
Nrg1-R	GTTGAGGCACCCTCTGAGAC
Nrg2-F	ACGGATTCTTCGGACAGAGAT
Nrg2-R	CACAGGACACTTTGCTTAGGAT
Nrg3-F	TTACGCTGTAGCGACTGCATC
Nrg3-R	GCCTACCACGATCCATTTAAGC
Nrg4-F	CACGCTGCGAAGAGGTTTTTC
Nrg4-R	CGCGATGGTAAGAGTGAGGA
Pdgfa-F	GAGGAAGCCGAGATACCCC
Pdgfa-R	TGCTGTGGATCTGACTTCGAG
Pdgfb-F	AAGTGTGAGACAATAGTGACCCC
Pdgfb-R	CATGGGTGTGCTTAAACTTTCG
Bmp4-F	TTCCTGGTAACCGAATGCTGA
Bmp4-R	CCTGAATCTCGGCGACTTTTT
Bmp7-F	ACGGACAGGGCTTCTCCTAC
Bmp7-R	ATGGTGGTATCGAGGGTGGAA
Bmp8a-F	ACATGCAGCGTGAAATCCTG
Bmp8a-R	GCGTGGTATAGGTCCAACATGA
Ins1-F	CACTTCCTACCCCTGCTGG
Ins1-R	ACCACAAAGATGCTGTTTGACA
Ins2-F	GCTTCTTCTACACACCCATGTC
Ins2-R	AGCACTGATCTACAATGCCAC
Tgfb1-F	CTCCCGTGGCTTCTAGTGC
Tgfb1-R	GCCTTAGTTTGGACAGGATCTG
Tgfb2-F	TCGACATGGATCAGTTTATGCG
Tgfb2-R	CCCTGGTACTGTTGTAGATGGA
**1.5 Cytotoxic T cell cytokines (positive control)**	
Ifng-F	ATGAACGCTACACACTGCATC

Ifng-R	CCATCCTTTTGCCAGTTCCTC
Gzmb-F	CCACTCTCGACCCTACATGG
Gzmb-R	GGCCCCCAAAGTGACATTTATT
Prf1-F	AGCACAAGTTCGTGCCAGG
Prf1-R	GCGTCTCTCATTAGGGAGTTTTT
Tnf-F	CCCTCACACTCAGATCATCTTCT
Tnf-R	GCTACGACGTGGGCTACAG
**2. Primers for VEGF-B regulated genes for lipid metabolism in T cells**	
Acsl5-F	TCCTGACGTTTGGAACGGC
Acsl5-R	CTCCCTCAATCCCCACAGAC
Cpt2-F	CAGCACAGCATCGTACCCA
Cpt2-R	TCCCAATGCCGTTCTCAAAAT
Fads1-F	AGCACATGCCATACAACCATC
Fads1-R	TTTCCGCTGAACCACAAAATAGA
Fads2-F	AAGGGAGGTAACCAGGGAGAG
Fads2-R	CCGCTGGGACCATTTGGTAA
Fasn-F	GGAGGTGGTGATAGCCGGTAT
Fasn-R	TGGGTAATCCATAGAGCCCAG
Scd1-F	TTCTTGCGATACACTCTGGTGC
Scd1-R	CGGGATTGAATGTTCTTGTCGT
Scd2-F	GCATTTGGGAGCCTTGTACG
Scd2-R	AGCCGTGCCTTGTATGTTCTG
**3. Primers for internal control in mouse sample**	
Actb-F	CTGAGAGGGAAATCGTGCGTGAC
Actb-R	GCTCGTTGCCAATAGTGATGACCTG
**4. Primers for human samples**	
VEGFB-F	GAGATGTCCCTGGAAGAACACA
VEGFB-R	GAGTGGGATGGGTGATGTCAG
FLT1-F	TTTGCCTGAAATGGTGAGTAAGG
FLT1-R	TGGTTTGCTTGAGCTGTGTTC
ACTB-F	CCTGGCACCCAGCACAAT
ACTB-R	GGGCCGGACTCGTCATAC

Table-S3. Antibodies and Materials.

**Name**	**Catalog No.#**	**Manufacturer**
anti-mouse CD3e	553057	BD Bioscience
anti-mouse CD28	553294	BD Bioscience
Purified anti-mouse CD16/32	101302	Biolegend
APC/Cy7 anti-mouse CD3e	557596	BD Bioscience
PerCP/Cy5.5 anti-mouse CD3e	100327	Biolegend
FITC anti-mouse CD3e	110031-82	eBioscience

APC anti-mouse CD4	100412	Biolegend
FITC anti-mouse CD4	553729	BD Biosciences
APC anti-mouse CD8a	100712	Biolegend
Alexa Fluor 594 anti-mouse CD8a	100758	Biolegend
PE anti-mouse CD8a	100707	Biolegend
FITC anti-mouse CD8a	100705	Biolegend
FITC anti-mouse CD62L	104406	Biolegend
PE/Cy7 anti-mouse CD62L	104418	Biolegend
APC/Cy7 anti-mouse CD44	103028	Biolegend
FITC anti-mouse CD44	561859	BD Bioscience
Brilliant Violet 421 anti-mouse/human CD44	103040	Biolegend
PE anti-mouse CD25	553075	BD Bioscience
APC/Cy7 anti-mouse CD127(IL-7Ra)	135040	Biolegend
PE/Cy7 anti-mouse/human KLRG1	138416	Biolegend
PerCP-Cy5.5 anti-Mouse CD45	550994	BD Bioscience
FITC anti-mouse CD45.2	109806	Biolegend
PerCP/Cy5.5 anti-mouse CD45.2	109828	Biolegend
PE anti-mouse IFN-γ	505808	Biolegend
APC anti-mouse IFN-γ	505810	Biolegend
Brilliant Violet anti-mouse IFN-γ	505830	Biolegend
PE anti-human/mouse Granzyme B	372208	Biolegend
T-Select H-2Kb OVA Tetramer -PE	TS-5001-1C	MBL
T-Select H-2Kb OVA Tetramer -APC	TS-5001-2C	MBL
PE anti-mouse IL-4	504104	Biolegend
PE/Cy7 anti-IL-17A	25-7177-80	eBioscience
PE anti-mouse IL-22 Antibody	516404	Biolegend
PE anti-mouse/rat/human FOXP3 Antibody	320008	Biolegend
PE/Cyanine7 anti-mouse CD366 (Tim-3)	119715	Biolegend
PE anti-mouse CD223 LAG-3	125207	Biolegend
PE anti-mouse CD279 (PD-1)	109103	Biolegend
PE anti-mouse/human CD11b Antibody	101207	Biolegend
Brilliant Violet 510 anti-mouse F4/80	123135	Biolegend
PE/Cyanine7 anti-mouse CD11c Antibody	117318	Biolegend
PE anti-mouse I-A/I-E (MHC class II)	107607	Biolegend
Brilliant Violet 510 anti-mouse Ly-6G/Ly-6C (Gr-1) Antibody	108437	Biolegend
AF488 anti-mouse CD19 Antibody	115521	Biolegend
PE anti-mouse NK-1.1 Antibody	108708	Biolegend
PE Rat Anti-Mouse Siglec-F	562068	BD Bioscience
7-AAD Viability Staining Solution	420404	Biolegend
Fixable Viability Dye eFluor 450	65-0863-14	eBioscience
LIVE/DEAD™ Fixable Violet Dead Cell	L34955	Thermo Fisher

Stain Kit, for 405 nm		
LIVE/DEAD™ Fixable Near-IR Dead Cell Stain Kit, for 633 or 635 nm	L34976	Thermo Fisher
EasySep™ Mouse Naive $CD8^{+}$ T Cell Isolation Kit	19858	STEMCELL
EasySep™ Mouse Naive $CD4^{+}$ T Cell Isolation Kit	19765	STEMCELL
EasySep™ Mouse CD8+ T Cell Isolation Kit	19853	STEMCELL
EasySep™ Human CD8+ T Cell Isolation Kit	17953	STEMCELL
EasySep™ Human CD4+ T Cell Isolation Kit	17952	STEMCELL
Human CD3/CD28 T Cell Activation Beads	422603	Biolegend
Recombinant Murine IL-2	212-12	PeproTech
Recombinant Murine IL-15	210-15	PeproTech
Recombinant Murine IL-4	214-14	PeproTech
Recombinant Murine IL-6	216-16	PeproTech
Recombinant Murine IL-12	210-12	PeproTech
Recombinant Mouse TGF-beta1 Protein	7666-MB	R&D System
Purified anti-mouse IL-4 Antibody	504101	Biolegend
Purified anti-mouse IL-12/IL-23 Antibody	505303	Biolegend
Purified anti-mouse IFN-γ Antibody	517905	Biolegend
Ovalbumin (257-264) chicken	S7951	Sigma
Cell Stimulation Cocktail	00-4975-93	eBioscience
Annexin V-FITC Apoptosis Detection Kit	BMS500FI-100	eBioscience
Annexin V-APC Apoptosis Detection Kit	88-8007-72	eBioscience
Cytofix/Cytoperm Fixation/ Permeabilization Kit	554714	BD Bioscience
True-Nuclear Transcription Factor Buffer Set	424401	Biolegend
MitoTracker Red	M7512	Invitrogen
MitoTracker Green	M7514	Invitrogen
MitoTracker Deep Red	M22426	Invitrogen
MitoProbe™ TMRM Kit	M20036	Invitrogen
MitoSOX	M36008	Invitrogen
Nonyl Acridine Orange (NAO)	MX4301	Maokangbio
Seahorse XF Cell Mito Stress Test Kit (OCR)	103015-100	Agilent
Seahorse XF Glycolysis Stress Test Kit (ECAR)	103020-100	Agilent
Mouse VEGFR1/Flt-1 Antibody (Neutralization)	AF471	R&D Systems

p-Thr (H-2) antibody	sc-5267	Santa cruz
Cytochrome c (D18C7) Rabbit mAb |	11940	Cell Signaling Technology
VDAC (D73D12) Rabbit mAb	4661	Cell Signaling Technology
Recombinant Anti-VEGF Receptor 1 antibody [Y103]	ab32152	Abcam
Caspase-3 (D3R6Y) Rabbit mAb	14220	Cell Signaling Technology
Cleaved Caspase-3 (Asp175) (5A1E) Rabbit mAb	9664	Cell Signaling Technology
Phospho-Tyrosine (P-Tyr-1000) MultiMab Rabbit mAb mix	8954	Cell Signaling Technology
Anti-PPAR gamma antibody	ab45036	Abcam
Anti-phospho-PPAR-γ (pSer273) antibody	600-401-J94	Thermo Fisher
Anti-phospho-PPAR-γ (pSer112) antibody	SAB4503977	Sigma aldrich
GABP-α Antibody (H-2)	sc-28311	Santa cruz
PGC1a Antibody (4A8)	sc-517380	Santa cruz

References

1. Kurachi M, et al. The transcription factor BATF operates as an essential differentiation checkpoint in early effector CD8+ T cells. *Nat Immunol.* 2014;15(4):373-83.
2. Schmiedel BJ, et al. Impact of Genetic Polymorphisms on Human Immune Cell Gene Expression. *Cell.* 2018;175(6):1701-15.e16.
3. Chandra V, et al. Promoter-interacting expression quantitative trait loci are enriched for functional genetic variants. *Nature Genetics.* 2021;53(1):110-9.
4. Becnel LB, et al. Discovering relationships between nuclear receptor signaling pathways, genes, and tissues in Transcriptomine. *Science signaling.* 2017;10(476).
5. Ochsner SA, et al. The Signaling Pathways Project, an integrated 'omics knowledgebase for mammalian cellular signaling pathways. *Scientific data.* 2019;6(1):252.
6. Fornes O, et al. JASPAR 2020: update of the open-access database of transcription factor binding profiles. *Nucleic acids research.* 2020;48(D1):D87-d92.
7. Ota M, et al. Dynamic landscape of immune cell-specific gene regulation in immune-mediated diseases. *Cell.* 2021;184(11):3006-21.e17.
8. Xing Y, et al. Dimerization of MICU Proteins Controls Ca(2+) Influx through the Mitochondrial Ca(2+) Uniporter. *Cell reports.* 2019;26(5):1203-12.e4.
9. Fan M, et al. Structure and mechanism of the mitochondrial Ca(2+) uniporter holocomplex. *Nature.* 2020;582(7810):129-33.
10. Wang T, et al. SENP1-Sirt3 Signaling Controls Mitochondrial Protein Acetylation and Metabolism. *Molecular cell.* 2019;75(4):823-34.e5.
11. He J, et al. Glucose limitation activates AMPK coupled SENP1-Sirt3 signalling in mitochondria for T cell memory development. *Nature communications.* 2021;12(1):4371.
12. Cai L, et al. Proteome-wide Mapping of Endogenous SUMOylation Sites in Mouse

Testis*. *Molecular & Cellular Proteomics.* 2017;16(5):717-27.
13. Nagy Á, et al. Pancancer survival analysis of cancer hallmark genes. *Scientific Reports.* 2021;11(1):6047.
14. Ru B, et al. TISIDB: an integrated repository portal for tumor–immune system interactions. *Bioinformatics.* 2019;35(20):4200-2.
