# Supplementary material for: Autocrine VEGF-B signaling maintains lipid synthesis and mitochondrial fitness to support T cell immune responses
Source: J Clin Invest. 2024 Aug 15;134(16):e176586. doi: 10.1172/JCI176586 (PMC11324299; doi:10.1172/JCI176586)

Full unedited gel for Figure

Full unedited gel for Figure 1B

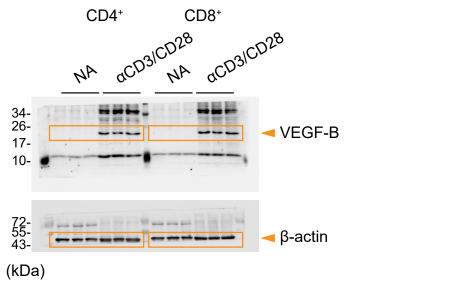

Full unedited gel for Figure 2A

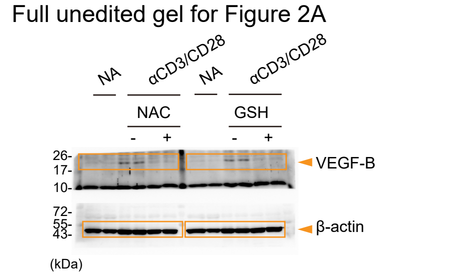

Full unedited gel for Figure 2B

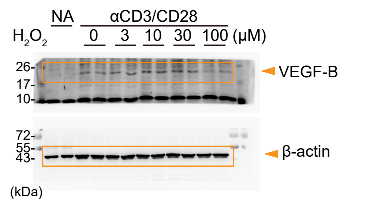

Full unedited gel for Figure 2D

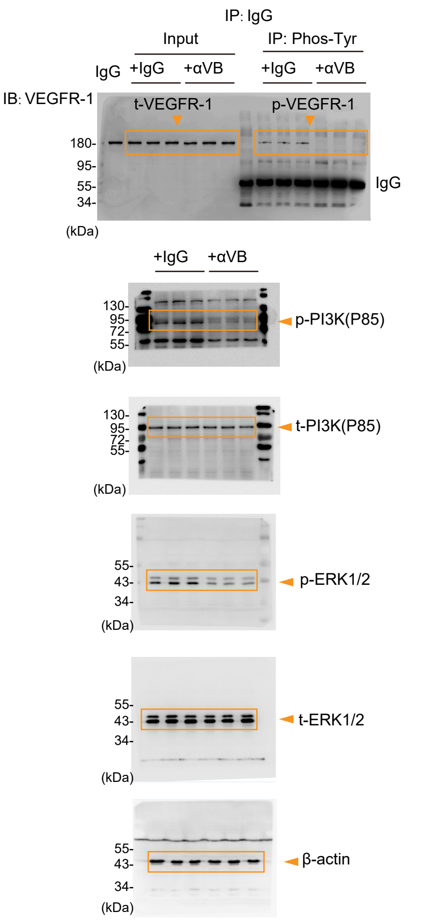

Full unedited gel for Figure 2E

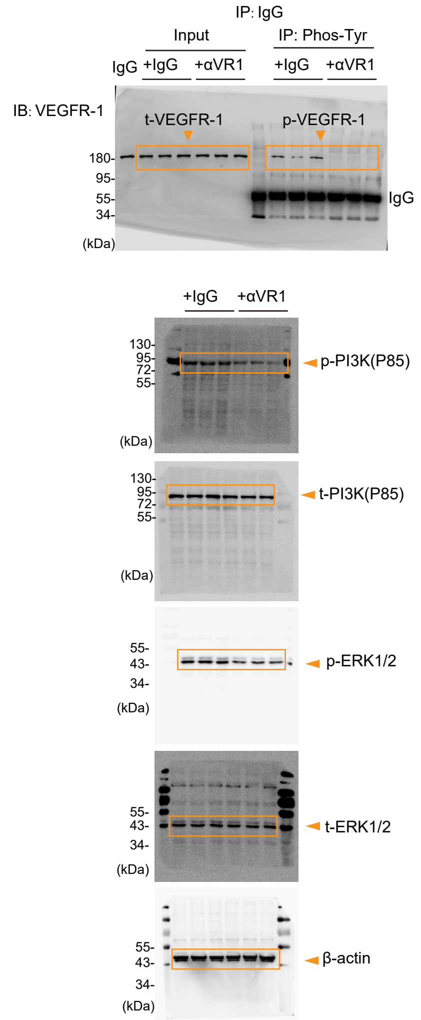

Full unedited gel for Figure 3E

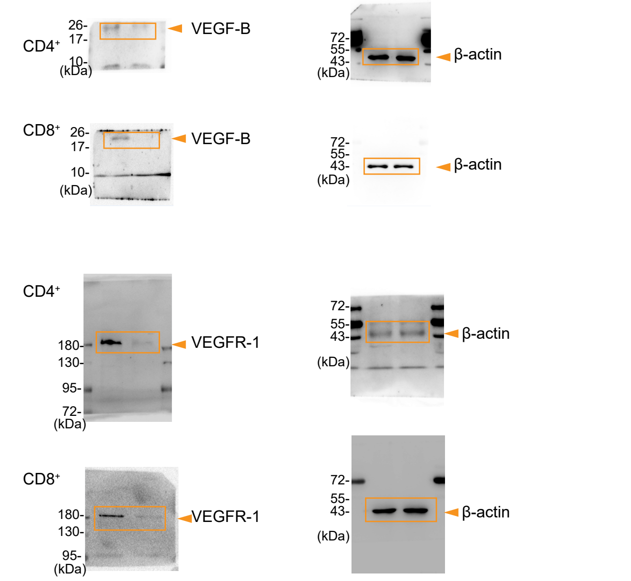

Full unedited gel for Figure 7A

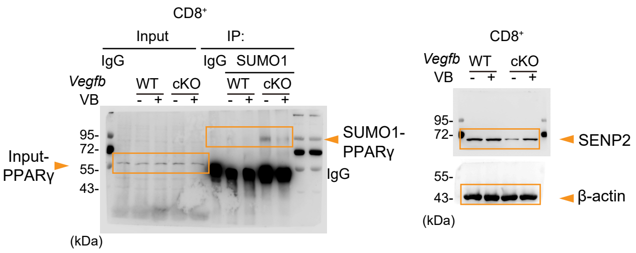

Full unedited gel for Figure 7C

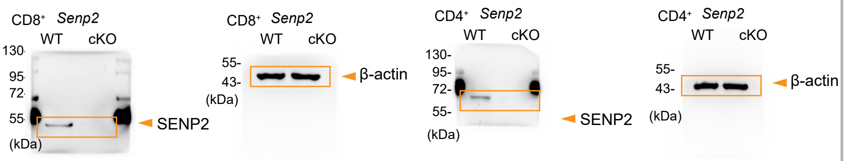

Full unedited gel for Figure 7I

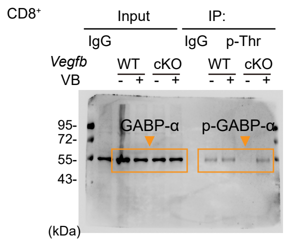

Full unedited gel for Supplemental Figures (Fig.S)

Full unedited gel for Fig. S1C

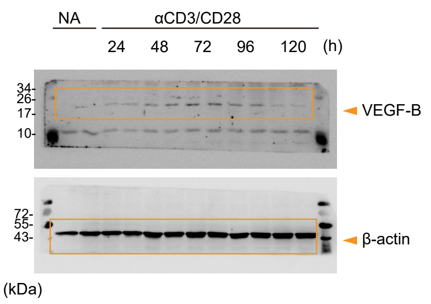

Full unedited gel for Fig.S2B

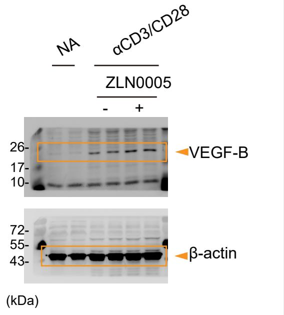

Full unedited gel for Fig.S2C

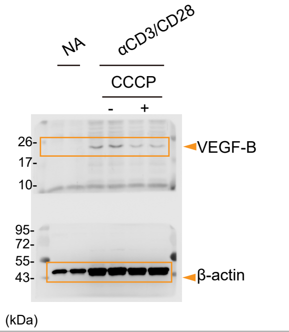

Full unedited gel for Fig.S2D

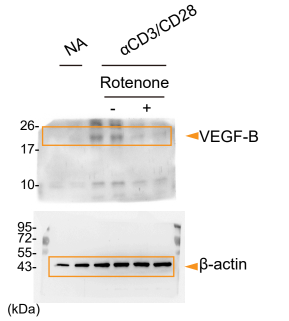

Full unedited gel for Fig.S2E

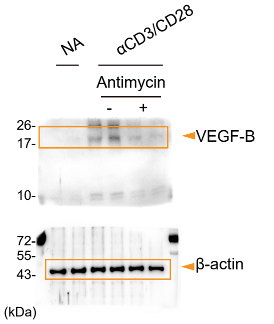

Full unedited gel for Fig.S2F

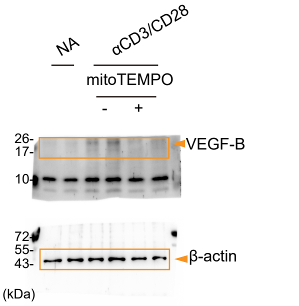

Full unedited gel for Fig.S2G

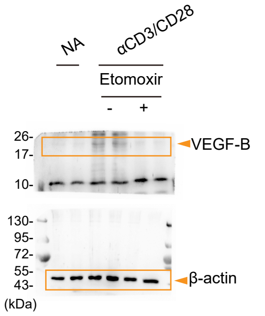

Full unedited gel for Fig.S2H

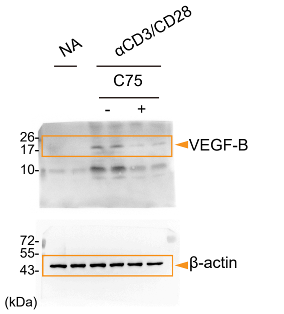

Full unedited gel for Fig.S3B

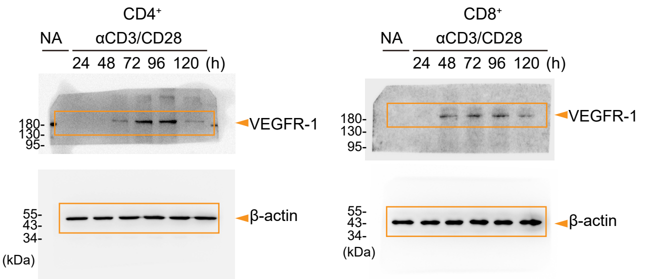

Full unedited gel for Fig.S4A

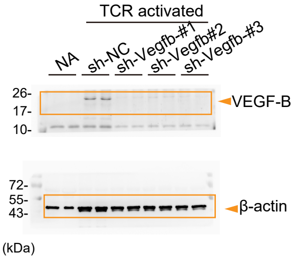

Full unedited gel for Fig.S5L

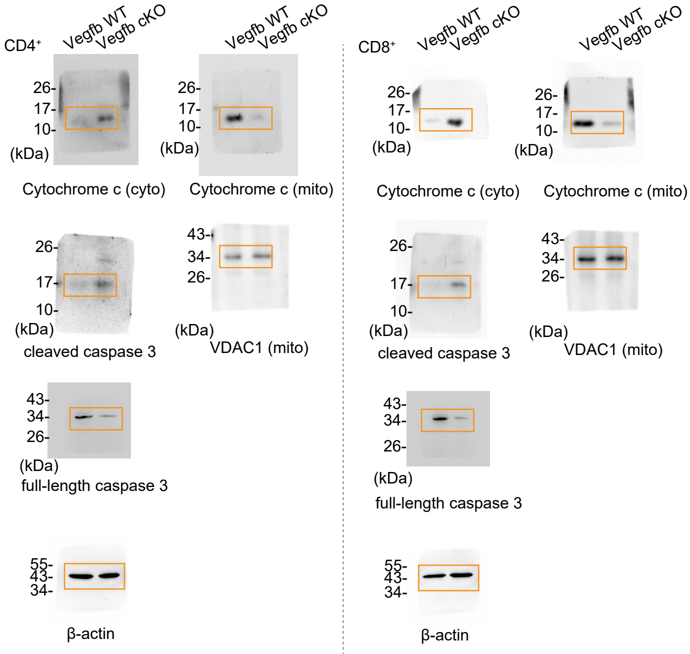

Full unedited gel for Fig.S9C

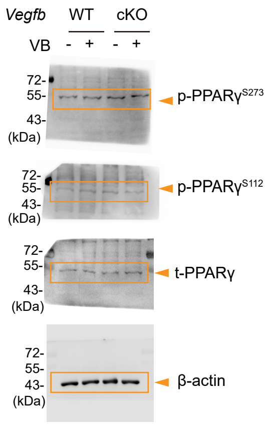

Full unedited gel for Fig.S9D

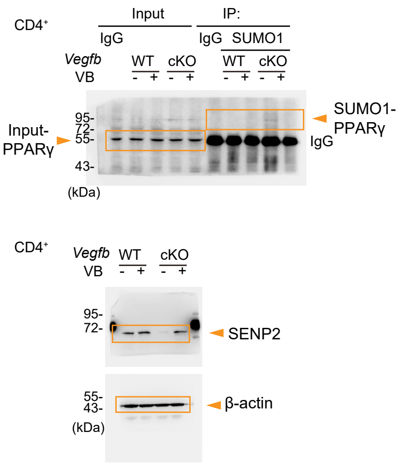

Full unedited gel for Supplemental Figures (Fig.S)

Full unedited gel for Fig.S11C

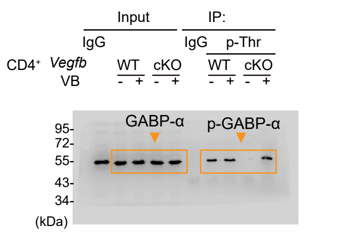

Full unedited gel for Fig.S11E

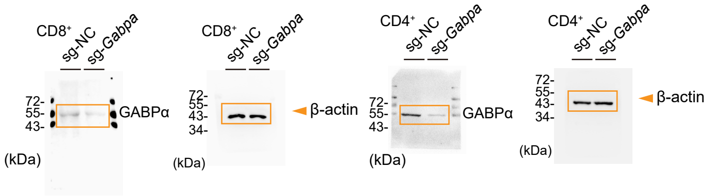

Full unedited gel for Fig.S13E

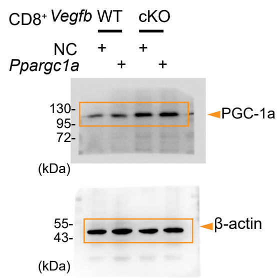

Full unedited gel for Fig.S15A

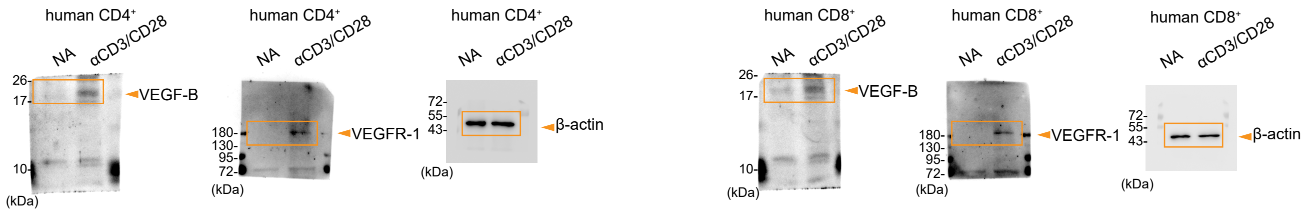

Full unedited gel for Fig.S15B

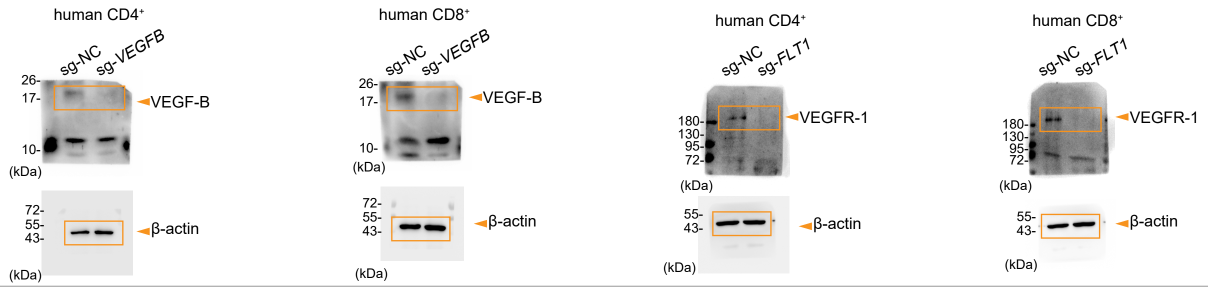

Supplement: Unedited blot and gel images [file jci-134-176586-s199.pdf]
